# A Potential Prognostic Marker PRDM1 in Pancreatic Adenocarcinoma

**DOI:** 10.1155/2022/1934381

**Published:** 2022-05-13

**Authors:** Bo Zhou, Jie Zhang, Hongda Zhu, Shugeng Wu

**Affiliations:** ^1^Department of General Surgery, Ningbo Medical Center Lihuili Hospital, Ningbo University, Ningbo, Zhejiang 315040, China; ^2^Medical School, Ningbo University, Ningbo, Zhejiang, China

## Abstract

Pancreatic adenocarcinoma (PAAD) is a major threat to people's health. PRDM1 is a transcription factor with multiple functions, and its functions have been validated in a variety of tumors; however, there are few studies reported on PRDM1 in PAAD. Using the GEPIA2 database, this research found that PRDM1 expression in PAAD was significantly higher than that in normal pancreatic tissue. The Kaplan-Meier Plotter database showed that high expression of PRDM1 in PAAD has a poor prognosis, suggesting that PRDM1 may be a potential prognostic marker in PAAD. The cBioPortal database shows that the expression of PRDM1 in PAAD is significantly correlated with its methylation degree. Further analysis on the coexpressed genes of PRDM1 in PAAD was performed by using LinkedOmics database to explore potential mechanisms. Based on gene enrichment analysis, PRDM1 was implicated in many pathways involved in tumor progression. In the construction of a PPI network of PRDM1 and its coexpressed gene protein via the STRING database, we found that PRDM1 may be involved in the pathogenesis and development of PAAD. TIMER database suggested that a high level of PRDM1 has a significant positive correlation with macrophages, neutrophils, and DCs. Potential methylation sites of PRDM1 were found through DNMIVD database, and MethSurv database explored eight sites which were significantly related with the prognosis of PAAD. In conclusion, PRDM1 may work as a prognostic marker or even provide a potential therapeutic strategy in PAAD.

## 1. Introduction

Pancreatic adenocarcinoma (PAAD) is a highly malignant tumor with an extremely poor prognosis and high mortality (less than 10% 5-year survival) and will be the third major cause of cancer-related deaths [1, 2]. Because of lacking early symptoms and effective detection methods, most patients(80%–85%) present with locally advanced or distant metastatic disease that cannot be resected [3, 4]. First-line treatment of metastatic pancreatic cancer is currently only effective in combination with chemotherapy with cytotoxic agents [5]. The median overall survival of patients with metastatic disease remains below 12 months [6]. Over the past decade, comprehensive annotation of tumor specimens for characterization has contributed to a better understanding of key genomic changes in PAAD, which includes molecular classification of tumors based on gene expression patterns as well as somatic mutations [7]. Besides, these studies have also revealed specific tumor therapeutic targets [8, 9]. Unfortunately, due to the potential for drug resistance and limitations, targeted therapy is only available for a small number of PAAD. Therefore, we hope to find a new research direction to better understand pancreatic cancer and explore possible treatment strategies.

PRDM1 is a transcription factor whose function is regulating transcriptional programs in both innate and adaptive immune system. Previous studies found that PRDM1 will influence the nonlymphoid organs excreting tissue-resident T cell populations by reducing the specific gene expression [10]; PRDM1 also can encode *β*-interferon gene repressor that specifically binds to the PRDI (positive regulatory domain I element) of the *β*-IFN gene promoter which can promote the differentiation of B lymphocytes into mature Ig-secreting cells [11]. In previous investigations, PRDM1 has shown its prognostic value in many tumors [12]. Li et al. show that PRDM1 expression correlates with the prognosis of HBV infection-related HCC, and high PRDM1 expression in HCC has a worse prognosis [13]. Zhu et al. found that the decreased expression of PRDM1 in lung cancer promoted cancer cell metastasis and correlated with poor prognosis of lung cancer, and PRDM1 functioned as a tumor suppressor in lung cancer [14]. Hu et al. reported that TGF-*β*1 inhibited prostate cancer progression by targeting PRDM1 [15]. However, its function in pancreatic cancer remains to be explored. In this bioinformatics analysis, we try to evaluate the role of PRDM1 in the clinical diagnosis and prognosis of PAAD and its potential value as a biomarker.

## 2. Methods

### 2.1. GEPIA2 Database

GEPIA2 (http://gepia.cancer-pku.cn/index.html) is a novel analytical tool. There are multiple tumor and normal tissue sample data on this platform [16]. In our study, we utilize the GEPIA2 database to analyze the differential gene expression, pathological staging, and related prognostic value of tumor tissue and normal tissue (*p* value was generated by *t*-test, |log2FC| > 2, *p* value cutoff < 0.01).

### 2.2. Kaplan-Meier Plotter Database

The Kaplan-Meier Plotter (http://kmplot.com/analysis/index.php?p=background) consists of data from the survival analysis of 54,675 genes in 10,461 cancer samples [17]. In this study, the Kaplan-Meier Plotter database was used to analyze the connection between PRDM1 and PAAD prognosis.

### 2.3. cBioPortal Database

cBioPortal (http://cbioportal.org) is a database that stores DNA copy number, mRNA expression, noncoding RNA, protein, and clinical information data. It is an open platform to study different cancer genome data using interactive exploration technology [18, 19]. In this study, we analyzed the TCGA pancreatic cancer data using the cBioPortal database to explore the mutation rate and distribution of PRDM1 in pancreatic cancer.

### 2.4. LinkedOmics Database

The LinkedOmics database (http://www.linkedomics.org/login.php) is an open platform including all 32 tumor type datasets in the TCGA database [20]. In this study, we used this database to screen the coexpressed genes of PRDM1, ranked according to the Spearman correlation coefficient, and plotted in the form of a volcano plot performance, and *p* values (*p* < 0.05) were used to screen for positively or negatively correlated genes.

### 2.5. DAVID Database

The DAVID database (DAVID https://david.ncifcrf.gov) is an open platform for gene annotation and visualization. The platform provides researchers with comprehensive gene annotation tools to better help us understand the biological significance of genes and then find beneficial research directions for us. GO and KEGG enrichment analysis helps us integrate extensive bioinformatics databases (including databases of genomes, biological pathways, diseases, drugs, and chemicals) to annotate genes from genomic or transcriptomic data and understand their biological characteristics and features [21]. We utilized the DAVID database to explore the annotation of PRDM1 coexpressed genes. Differences were considered statistically significant with *p* value < 0.05 and FDR < 0.05.

### 2.6. STRING Database

The STRING database (http://www.string-db.org) is an open platform for protein annotation [22]. By integrating multiple resources, the STRING database can be used to construct the PPI network of PRDM1.

### 2.7. TIMER Database

The TIMER database (http://timer.cistrome.org/) contains immune cell infiltration data of various tumors in the TCGA database [23]. Relying on TIMER database, we performed an immune infiltration analysis of PRDM1 to explore the relationship between PRDM1 and tumor immune cells.

### 2.8. TISIDB Database

TISIDB (http://cis.hku.hk/TISIDB/) integrates multiple datasets to form a database of tumor-immune cell interaction through literature mining on PubMed. In addition, the TISIDB database also integrates resources from UniProt, GO, DrugBank, and other databases [24]. The TISIDB database was used to investigate whether PRDM1 was related to immune checkpoints in PAAD.

### 2.9. DNMIVD Database

DNMIVD database (http://119.3.41.228/dnmivd/index/) is a methylation chip based on TCGA and GEO database building of methylation carcinoma analysis database; the database can view a gene methylation in the generic cancer sites [25]. In this study, CpGs loci of PRDM1 will be searched through this database for subsequent analysis.

### 2.10. MethSurv Database

MethSurv database (http://biit.cs.ut.ee/methsurv/) is a use of DNA methylation data network multivariable survival analysis tool [26]. This study will explore the methylation site of PRDM1 in pancreatic cancer and its effect on the survival time of pancreatic cancer patients through this database.

## 3. Results

### 3.1. The Expression Difference of PRDM1 in Pancreatic Cancer and Normal Pancreatic Tissue

**Figure 1:**
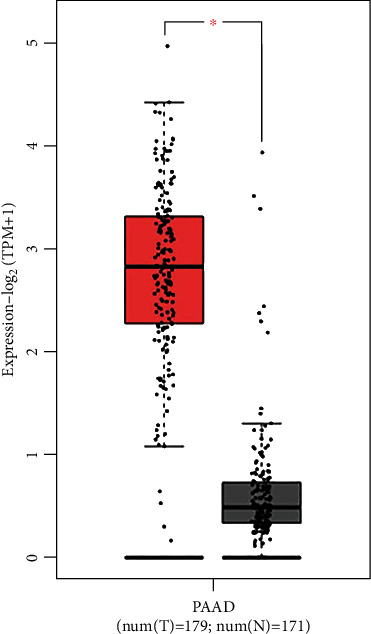
Expression of PRDM1 in PAAD and normal pancreas tissues revealed by GEPIA2 database. ^∗^*p* value < 0.01.

We used the GEPIA2 database to obtain PRDM1 gene expression data of tumor samples and corresponding normal pancreatic tissues, thus detecting differences in PAAD of PRDM1 compared to normal tissues. As shown in Figure 1, the expression of PRDM1 in PAAD was significantly higher than that in normal pancreas tissues.

### 3.2. The Prognostic Value of PRDM1 in PAAD

**Figure 2:**
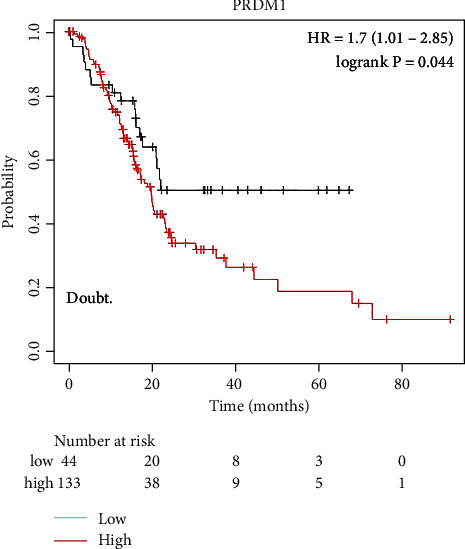
The Kaplan-Meier Plotter database demonstrates the effect of PRDM1 expression levels on survival in PAAD patients.

We further explored the effect and correlation of PRDM1 expression on overall survival (OS) in PAAD patients by using the Kaplan-Meier Plotter (Figure 2). We found that high PRDM1 expression was significantly and negatively correlated with OS in PAAD (*p* = 0.044). The results showed that the higher expression of PRDM1 in PAAD was significantly associated with poorer prognosis, suggesting that PRDM1 may be a potential prognostic marker of PAAD.

### 3.3. PRDM1 Mutation Analysis and Its Correlation with Methylation

**Figure 3:**
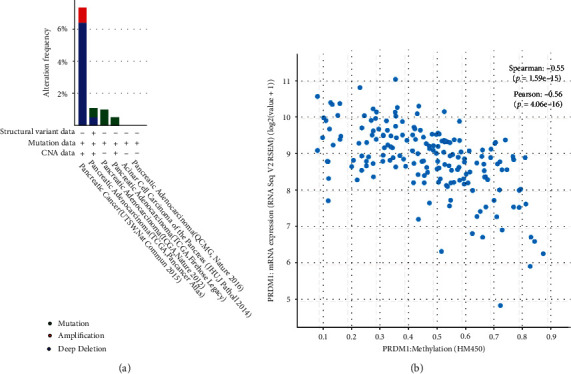
(a) The gene mutation rate of PRDM1 in PAAD is displayed through the cBioPortal database. (b) Correlation between PRDM1 expression and methylation.

It is now widely recognized that genomic mutations are strongly associated with tumorigenesis. So we conducted a comparative research of PRDM1 in order to find out the genomic mutation of PRDM1 in PAAD. We firstly detected the genetic alterations of PRDM1 in PAAD through the cBioPortal database. Mutation profile of PRDM1 gene shows that deep deletion is one of the most important factors of PAAD gene mutation (Figure 3(a)). In addition, PRDM1 expression was significantly correlated with its methylation level (Figure 3(b)) (Spearman: -0.55, *p* = 1.59*e* − 15; Pearson: -0.56, *p* = 4.06*e* − 16).

### 3.4. PRDM1 Coexpression in PAAD

**Figure 4:**
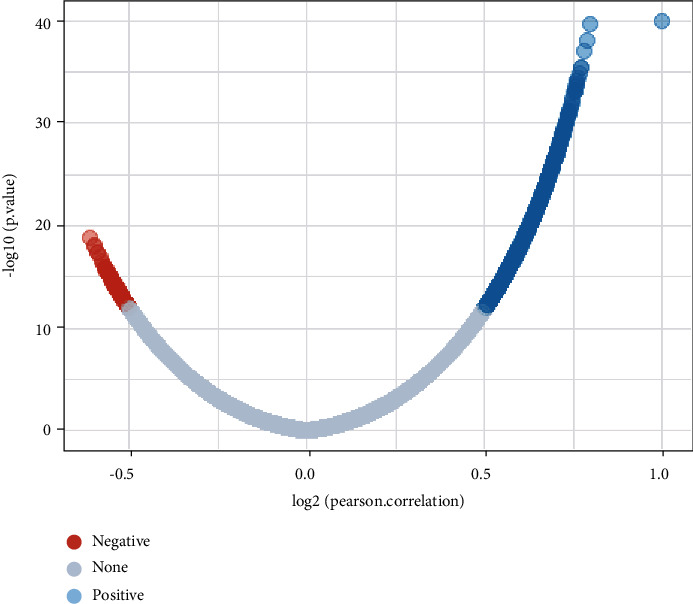
Coexpressed genes of PRDM1 in PAAD (LinkedOmics).

Through the above analysis, we found that PRDM1 has significant prognostic value in PAAD, so another database (LinkedOmics) was applied to detect the coexpressed genes of PRDM1 in PAAD and analyze the potential biological and functional mechanism. The coexpression genes in PAAD were determined by Pearson's correlation test (*p* < 0.05, |Coefficient| > 0.5). The results showed that a total of 1280 genes were significantly associated with PRDM1, of which 1145 genes had positive correlations and 135 genes had negative correlations (Figure 4). In Supplementary Tables S1 and S2, we can see the coexpressed genes positively or negatively associated with PRDM1. Further analysis was performed using coexpressed genes.

### 3.5. Functional Enrichment of the PRDM1-Related Genes

**Figure 5:**
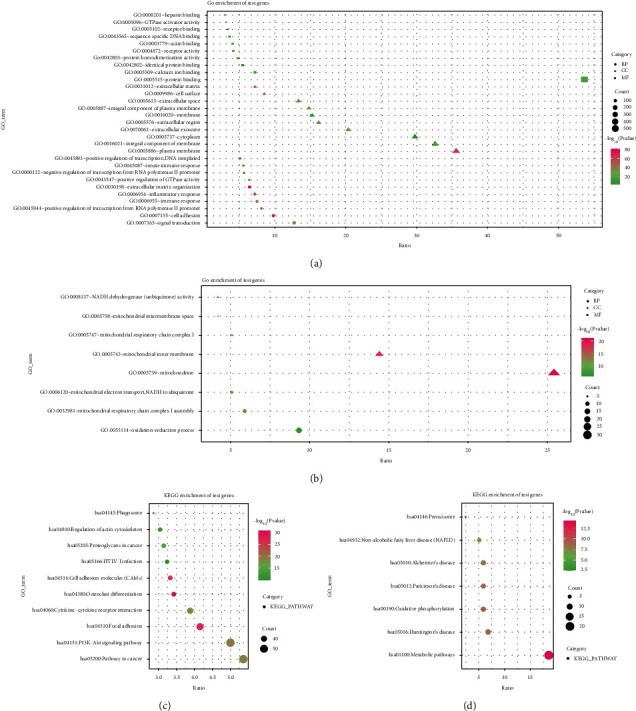
(a) GO analysis of PRDM1 positive coexpressed genes. (b) GO analysis of PRDM1 negative coexpression genes. (c) KEGG analysis of PRDM1 positive coexpression genes. (d) KEGG analysis of PRDM1 negative coexpression genes.

To investigate the molecular mechanism of PRDM1 regulation of PAAD in depth, we performed GO and KEGG analyses of PAAD. Annotation of PRDM1 coexpressed genes was performed using the DAVID database. We choose top 10 GO terms that positive correlation coexpression genes (Figure 5(a)), and top 8 GO term that negative correlation coexpression genes (Figure 5(b)). We also choose top 10 KEGG pathways of positive correlation coexpression genes (Figure 5(c)) and 7 pathways of negative correlation coexpression genes (Figure 5(d)). These positive correlation genes were associated with “signal transduction” (GO~BP), “plasma membrane” (GO~CC), “protein binding” (GO~MF), and “pathways in cancer” (KEGG), and negative correlation genes were associated with “oxidation-reduction process” (GO~BP), “mitochondrion” (GO~CC), “NADH dehydrogenase (ubiquinone) activity” (GO~MF), and “metabolic pathways” (KEGG). All these data were detailed in Supplementary Tables S3, S4, S5, and S6.

### 3.6. PPI Network of PRDM1 Coexpression

**Figure 6:**
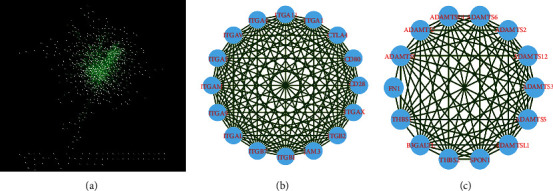
The PPI (protein-protein interaction) network was created by STRING database. (a) PPI network constructed with PRDM1 coexpression genes. (b and c) Using molecular complexity detection (MCODE) method to identify significant modules from PPI network with score ≥ 10. Panel (b) shows the module 1 with an MCODE score of 16. Panel (c) shows the module 2 with an MCODE score of 12.308.

**Figure 7:**
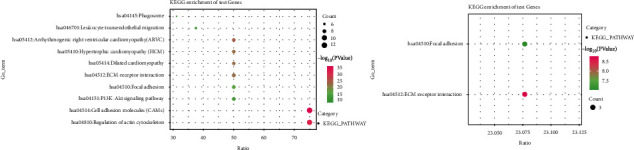
Enrichment pathways for module 1 and module 2 are displayed by KEGG pathway enrichment analysis. *p* < 0.05.

Molecular mechanisms (major physiological and pathological changes) driving cancer progression can be demonstrated by protein-protein interaction (PPI) analysis. We used the STRING database to construct a PPI network of PRDM1 and its coexpressed chaperones. Through the analysis of the STRING database, these proteins were screened out with a comprehensive score ≥ 0.8. In addition, through MCODE tool, there are two significant modules (modules 1 (Figure 6(b)) and 2 (Figure 6(c))) with a score ≥ 10 screened (degree cutoff = 2, node score cutoff = 0.1, and k − core = 2). The results showed that the network of coexpressed genes was composed of 810 nodes and 3187 edges (Figure 6(a)). In addition, the network of module 1 consisted of 16 nodes and 120 edges, and module 2 consists of 14 nodes and 80 edges.  Figure 7 shows the enrichment pathways of module 1 and module 2, the most important pathways in module 1 and module 2 are enriched in the “regulation of actin cytoskeleton” and “cell adhesion molecules (CAMs).”

### 3.7. PRDM1 Influenced the Extent of Immune Infiltration in PAAD

**Figure 8:**
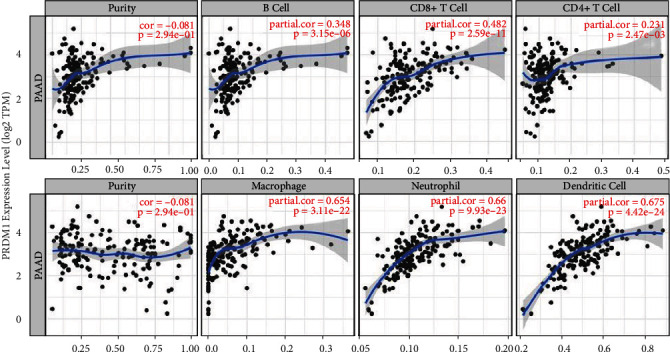
The correlation between PRDM1 expression and immune cells levels in PAAD was analyzed using the TIMER database.

Nowadays, there are various approaches for exploring novel prognostic biomarkers and developing new cancer treatments, among which discovering the interaction between the host immune defense microenvironment and various tumor is a very important one. Based on current research, we know that the overall survival of patients is affected by immune cell infiltration in the tumor microenvironment. Previous studies have found that the expression of PRDM1 has some correlation with immune infiltration, so we analyzed the relationship between the two in PAAD according to the TIMER database. As shown in Figure 8, a high level of PRDM1 has a significant positive correlation with macrophages, neutrophils, and DCs.

### 3.8. The Regulation of Immune Molecules by PRDM1

**Figure 9:**
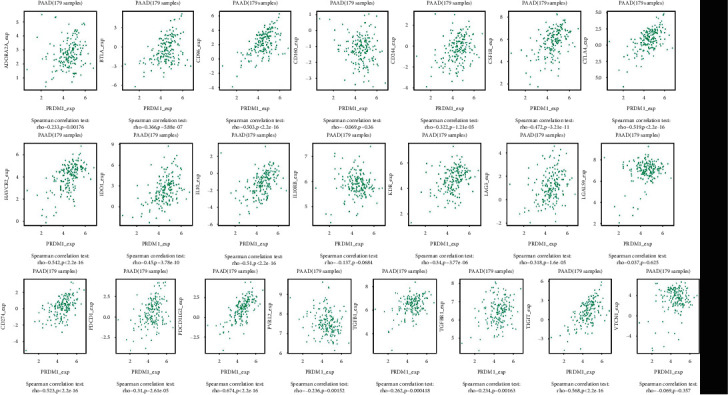
Correlation analyses of the PRDM1 expression with immunoinhibitor genes in PAAD via TISIDB.

Using the TISIDB database, we further explored whether PRDM1 is an important factor associated with immune infiltration. In Figure 9, we can see that the expression of PRDM1 is correlated with several immunoinhibitors. PDCD1LG2 (Spearman correlation test: r = 0.674, *p* < 2.2e − 16) displayed the greatest correlations with PRDM1 expression in PAAD. PDCD1LG2 is a gene that plays a crucial role in the growth and development of T cells. In addition, PDCD1LG2 can also participate in costimulatory signaling and also play a role in the production of IFNG. PDCD1LG2 can interact with PDCD1 to inhibit T cell proliferation, which is mainly achieved by blocking cytokine production and cell cycle. These findings suggest that immune fingerprinting may play an unusual role in PAAD.

### 3.9. PRDM1 CpG Island

Through DNMIVD database, we explored CpG island of PRDM1, and these sites may also be potential methylation sites. By looking for these sites, we will discuss the correlation between PRDM1 methylation sites and prognosis of PAAD in the following analysis. CpG island is shown in Table 1.

### 3.10. The Prognostic Value of CpG Island Methylation of PRDM1 in PAAD

**Figure 10:**
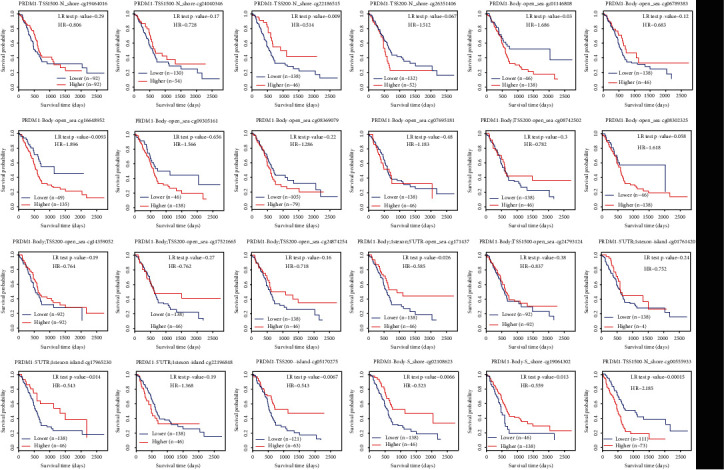
The prognostic value of CpG island methylation of PRDM1 in PAAD.

We analyzed the prognostic value of PRDM1 methylation in PAAD in the MethSurv database, through which we found eight methylation sites with significant survival differences in PAAD (Figure 10). PRDM1-Body-S_Shore-cg02108623, PRDM1-Body-S_Shore-cg19064302, and PRDM1-5′UTR; 1stExon-Island-cg17965230, PRDM1-TSS200-Island-cg05170275, and PRDM1-Body; 1stExon; and 5′UTR-Open_Sea-cg17143179 and PRDM1-TSS200-N_Shore-cg22186515 had a better prognosis for PAAD. However, hypermethylation of PRDM1-Body-Open_Sea-cg16648952 and PRDM1-TSS1500-N_Shore-cg00555933 had worse prognosis for PAAD.

## 4. Discussion

PAAD is a highly malignant tumor; the widely accepted view is that PAAD develops from a precancerous lesion to a varied, complex process. PAAD is a heterogeneous disease characterized by accumulation of alterations in the epigenetic domain and inherited characteristics over the time as PAAD progresses [27]. Because PAAD has a poor prognosis, it is an urgent to find corresponding strategies for the diagnosis and treatment of PAAD.

The PR/SET structural domain shares an isoform of the SET structural domain, the PRDF1-RIZ (PR) homologous structural domain. There are 19 functionally distinct transcription factors that are encoded and regulated by the PRDM gene family. This domain has a number of zinc fingers and has methyltransferase activity. These domains may make the PRDM family have the function of mediating protein-protein, protein-RNA, and protein-DNA interactions [28]. PRDM proteins attach transcription factors to target DNA promoters, and this is done by recognizing specific common sequences or by acting as non-DNA binding cofactors [29]. PRDM showed strong environmental dependence by selecting different target promoters and binding sites. In addition, PRDM is involved in many signal transductions that control cell lifespan and homeostasis. Some evidence suggest that the PRDM gene family plays an important role in the evolution of human malignancies through epigenetic modification, genetic reediting, inflammation, metabolic homeostasis, and other processes [29, 30]. At present, there are many studies on PRDM family trying interpreting its functions, but there are few studies on PRDM1 in PAAD. So our objective was to explore the role of PRDM1 in PAAD from the perspectives of the gene expression level and prognosis, gene pathway, genomics, methylation direction, and immune infiltration.

In the present study, based on the GEPIA2 database, we observed that the mRNA expression of PRDM1 was significantly increased in PAAD, while the expression was low in normal pancreatic tissue (Figure 1). The Kaplan-Meier Plotter database shows that the higher the expression of PRDM1, the worse the prognosis (Figure 2). These results suggest that PRDM1 may serve as a prognostic biomarker in PAAD. And the biological role of PRDM1 in PAAD still deserved to be investigated.

Then, we explored the functions of PRDM1 and its coexpressed genes using LinkedOmics database as well as GO and KEGG analyses to validate the underlying molecular mechanism of PRDM1 in PAAD and elucidate its value (Figure 4). Most of the PRDM1 coexpressed genes were mainly enriched in the protein-binding pathway. In addition to this, the enrichment ratio of integral component of membrane, cytoplasm, and extracellular exosome was more than 20% (Figure 5(a)). These results suggest that PRDM1 coexpressed genes may play a crucial role in the synthesis of cell membrane or organelle membrane proteins and are involved in the synthesis of these substances. And KEGG analysis revealed that PRDM1 may be involved in regulating cell membrane or organelle membrane proteins in cancer (Figure 5(c)). In the negative coexpression genes, GO and KEGG analyses suggest that PRDM1 may alter tumor metabolism (Figures 5(b) and 5(d)). Next, coexpression gene network complex of 810 nodes and 3187 edges was constructed via the Cytoscape software and STRING database (Figure 6(a)). Then, two modules were filtered out from the PPI network complex by Cytoscape MCODE analysis (Figures 6(b) and 6(c)). The results suggest that integrin subunit *α* and ADAMTS (A disintegrin-like and metalloprotease (reprolysin-type) with thrombospondin type1 motif) superfamily were screened out. Integrins are a class of cell adhesion molecules that mediate intercellular and extracellular matrix interactions [31]. ADAMTS proteins are a superfamily of 26 secreted molecules, including two related but distinct families [32]. The main substrate of ADAMTS protease is the extracellular matrix fraction, which is zinc endopeptidase in nature. ADAMTS proteins have many potential associations with other human diseases. These results show that PRDM1 plays a significant role in the progression of PAAD.

Next, we investigated the role of PRDM1 in the immune microenvironment. The present study revealed the connection between PRDM1 and PAAD immune cells on the basis of the level of PRDM1 expression and the immune status of PAAD. The result suggested that PRDM1 significantly associated with the level of immune cell infiltration, especially macrophages, neutrophils, and DCs (Figure 8). The significant correlation between PRDM1 expression and immunosuppressants was also verified, and as shown in Figure 9, the largest correlation was PDCD1LG2. This research may provide a new direction for immunotherapy in PAAD.

In addition, studies over the past decades have shown that epigenetic alterations, including aberrant DNA methylation, altered expression levels of various noncoding RNA, and aberrant histone modifications, always occur early and manifest frequently in cancer [33]. The present study showed that with the increase of PRDM1 expression, its methylation degree also increased (Figure 3(b)). We used DNMIVD database to find CpG islands of PRDM1 (Table 1) and MethSurv database to correlate methylation of these CpG islands with the prognosis of PAAD (Figure 10). Then, we found that the degree of PRDM1 methylation was significantly correlated with the prognosis of PAAD. The relationship between DNA methylation and PRDM1 expression deserves further research.

## 5. Conclusion

In conclusion, we applied bioinformatics analysis to study the role of PRDM1 in PAAD, we found that higher expression of PRDM1 was associated with a poorer prognosis in PAAD patients, and PRDM1was closely related to PAAD immunopathogenesis. However, it should be noted that there are some limitations to this study. For one thing, different databases have their own algorithms and periodic data update, and the potential systematic bias was unavoidable. For another, our data need to be supported by an *in vivo/in vitro* experimental study, such as microarray or proteomic analyses, which we are going to perform. In the future, we believe PRDM1 may work as a potential prognostic marker for PAAD and even offer a novel approach to antitumor in the future.

## Figures and Tables

**Table 1:** The CpG island of PRDM1.

CpG	Group	Relation to island
cg00555933	TSS1500	N_Shore
cg01146808	Body	OpenSea
cg01761420	1stExon; 5′UTR	Island
cg02108623	Body	S_Shore
cg05170275	TSS200	Island
cg06789383	Body	OpenSea
cg07695181	Body	OpenSea
cg08302325	Body	OpenSea
cg08358263	3′UTR	OpenSea
cg08369079	Body	OpenSea
cg08742502	Body; TSS200	OpenSea
cg09305161	Body	OpenSea
cg14359052	Body; TSS200	OpenSea
cg16648952	Body	OpenSea
cg17143179	Body; 1stExon; 5′UTR	OpenSea
cg17521665	Body; TSS200	OpenSea
cg17965230	1stExon; 5′UTR	Island
cg19064302	Body	S_Shore
cg19464016	TSS1500	N_Shore
cg22186515	TSS200	N_Shore
cg22196848	1stExon; 5′UTR	Island
cg24040346	TSS1500	N_Shore
cg24793124	Body; TSS1500	OpenSea
cg24874254	Body; TSS200	OpenSea
cg26351406	TSS200	N_Shore

## Data Availability

The datasets used and analyzed during the current study are available from the corresponding author on reasonable request.

## Supplementary Materials

Supplementary 1 Table S1: positive gene associated with PRDM1 in LinkedOmics database.

Supplementary 2 Table S2: negative gene associated with PRDM1 in LinkedOmics database.

Supplementary 3 Table S3: the coexpressed genes with positive correlation between GO enrichment pathway and PRDM1 via DAVID database.

Supplementary 4 Table S4: the coexpressed genes with negative correlation between GO enrichment pathway and PRDM1 via DAVID database.

Supplementary 5 Table S5: the coexpressed genes with positive correlation between KEGG pathway and PRDM1 via DAVID database.

Supplementary 6 Table S6: the coexpressed genes with negative correlation between KEGG pathway and PRDM1 via DAVID database.
