# Supplementary material for: A Potential Prognostic Marker PRDM1 in Pancreatic Adenocarcinoma
Source: J Oncol. 2022 May 13;2022:1934381. doi: 10.1155/2022/1934381 (PMC9123419; doi:10.1155/2022/1934381)
Supplement: Supplementary 1 — Table S1: positive gene associated with PRDM1 in LinkedOmics database. [file 1934381.f1.docx]

**Table S1.** Positive gene associated with PRDM1 in LinkedOmics database.

| Gene | Pearson-correlation | P-value |
| --- | --- | --- |
| A2M | 0.501797143 | 9.65E-13 |
| ABCA1 | 0.548073372 | 2.41E-15 |
| ABCA6 | 0.512995118 | 2.46E-13 |
| ABI3BP | 0.50776419 | 4.69E-13 |
| ABL2 | 0.505807539 | 5.95E-13 |
| ACAP2 | 0.528314603 | 3.48E-14 |
| ACPL2 | 0.555631643 | 8.27E-16 |
| ACSL4 | 0.522177075 | 7.71E-14 |
| ACSS3 | 0.619583347 | 2.97E-20 |
| ACTA2 | 0.626255908 | 8.90E-21 |
| ACTR2 | 0.550869263 | 1.63E-15 |
| ACVRL1 | 0.518789343 | 1.19E-13 |
| ADAM12 | 0.725962686 | 2.00E-30 |
| ADAM17 | 0.500982211 | 1.06E-12 |
| ADAM19 | 0.691588231 | 1.17E-26 |
| ADAMDEC1 | 0.517137424 | 1.46E-13 |
| ADAMTS12 | 0.717774542 | 1.78E-29 |
| ADAMTS2 | 0.632990995 | 2.56E-21 |
| ADAMTS3 | 0.631445208 | 3.41E-21 |
| ADAMTS4 | 0.676474712 | 3.67E-25 |
| ADAMTS5 | 0.61613619 | 5.48E-20 |
| ADAMTS6 | 0.535960952 | 1.26E-14 |
| ADAMTS7 | 0.552478263 | 1.30E-15 |
| ADAMTSL1 | 0.54211916 | 5.49E-15 |
| ADAMTSL4 | 0.531250308 | 2.37E-14 |
| ADAP2 | 0.555226872 | 8.77E-16 |
| ADARB1 | 0.549232361 | 2.05E-15 |
| AEBP1 | 0.690997801 | 1.35E-26 |
| AFAP1 | 0.524839876 | 5.47E-14 |
| AIM2 | 0.506789192 | 5.28E-13 |
| AKAP12 | 0.538574372 | 8.89E-15 |
| AKAP13 | 0.531902954 | 2.17E-14 |
| AKAP2 | 0.616453673 | 5.18E-20 |
| ALDH1A3 | 0.571275667 | 8.29E-17 |
| ALDH1L2 | 0.512391483 | 2.65E-13 |
| ALOX5AP | 0.533525158 | 1.75E-14 |
| ALPK2 | 0.721483265 | 6.68E-30 |
| AMOTL1 | 0.54557205 | 3.41E-15 |
| AMOTL2 | 0.502863033 | 8.49E-13 |
| ANGPT1 | 0.569667689 | 1.06E-16 |
| ANGPTL2 | 0.621730217 | 2.02E-20 |
| ANO6 | 0.529696572 | 2.90E-14 |
| ANTXR1 | 0.737012415 | 9.25E-32 |
| AOC3 | 0.648510696 | 1.28E-22 |
| APBA2 | 0.547018692 | 2.79E-15 |
| APBB1IP | 0.519346976 | 1.11E-13 |
| APBB2 | 0.564528448 | 2.27E-16 |
| APOBEC3C | 0.562950649 | 2.86E-16 |
| AQP9 | 0.52686294 | 4.21E-14 |
| ARHGAP20 | 0.577709209 | 3.11E-17 |
| ARHGAP25 | 0.615894796 | 5.72E-20 |
| ARHGAP28 | 0.543890721 | 4.30E-15 |
| ARHGAP30 | 0.613661976 | 8.46E-20 |
| ARHGAP31 | 0.640712491 | 5.89E-22 |
| ARHGAP42 | 0.581874196 | 1.63E-17 |
| ARHGAP9 | 0.516354045 | 1.61E-13 |
| ARHGDIB | 0.562732302 | 2.96E-16 |
| ARHGEF6 | 0.636513678 | 1.32E-21 |
| ARID5B | 0.634742244 | 1.84E-21 |
| ARL11 | 0.576608085 | 3.68E-17 |
| ARL13B | 0.507151673 | 5.05E-13 |
| ARL4C | 0.592524844 | 2.98E-18 |
| ARRDC3 | 0.555940251 | 7.92E-16 |
| ARSB | 0.556691339 | 7.11E-16 |
| AR | 0.544324527 | 4.05E-15 |
| ASAM | 0.714249586 | 4.44E-29 |
| ASAP1 | 0.612683485 | 1.00E-19 |
| ASPN | 0.702384703 | 8.80E-28 |
| ATP10A | 0.564875551 | 2.16E-16 |
| ATP11C | 0.657204672 | 2.22E-23 |
| ATP6V0D2 | 0.512916863 | 2.48E-13 |
| ATP8B2 | 0.516814649 | 1.52E-13 |
| ATP8B4 | 0.557538827 | 6.29E-16 |
| ATXN1 | 0.718535981 | 1.46E-29 |
| AVPR1A | 0.551980565 | 1.39E-15 |
| AXL | 0.644210108 | 2.99E-22 |
| B2M | 0.540745233 | 6.62E-15 |
| B3GALTL | 0.522864532 | 7.06E-14 |
| BACH1 | 0.576356332 | 3.83E-17 |
| BACH2 | 0.513099482 | 2.43E-13 |
| BASP1 | 0.698044999 | 2.53E-27 |
| BCL2A1 | 0.614117896 | 7.81E-20 |
| BCL6 | 0.535546632 | 1.34E-14 |
| BEND6 | 0.524268926 | 5.89E-14 |
| BEST1 | 0.531778101 | 2.21E-14 |
| BGN | 0.655951588 | 2.87E-23 |
| BHLHE22 | 0.500777408 | 1.09E-12 |
| BHMT2 | 0.521432941 | 8.48E-14 |
| BICC1 | 0.582632149 | 1.44E-17 |
| BICD1 | 0.532025274 | 2.14E-14 |
| BIN2 | 0.577653198 | 3.13E-17 |
| BMP1 | 0.503986938 | 7.42E-13 |
| BMP2K | 0.581196113 | 1.81E-17 |
| BMP8A | 0.549586025 | 1.95E-15 |
| BMPR2 | 0.575035465 | 4.68E-17 |
| BNC2 | 0.753478072 | 7.02E-34 |
| BNIP2 | 0.638483595 | 9.04E-22 |
| BOC | 0.513294552 | 2.37E-13 |
| BST1 | 0.599641158 | 9.25E-19 |
| BTBD19 | 0.52597102 | 4.72E-14 |
| BTK | 0.605418093 | 3.50E-19 |
| BTN2A2 | 0.576428768 | 3.78E-17 |
| BTN3A1 | 0.553557196 | 1.11E-15 |
| BTN3A3 | 0.523532888 | 6.48E-14 |
| C10orf128 | 0.567232278 | 1.52E-16 |
| C10orf26 | 0.518845297 | 1.18E-13 |
| C10orf55 | 0.502493162 | 8.88E-13 |
| C10orf72 | 0.674772293 | 5.34E-25 |
| C13orf18 | 0.530531278 | 2.60E-14 |
| C14orf118 | 0.506096728 | 5.74E-13 |
| C14orf37 | 0.584131292 | 1.14E-17 |
| C14orf49 | 0.619294452 | 3.13E-20 |
| C17orf87 | 0.535554465 | 1.34E-14 |
| C18orf54 | 0.526218521 | 4.57E-14 |
| C1QC | 0.51133229 | 3.02E-13 |
| C1QTNF3 | 0.552091743 | 1.37E-15 |
| C1QTNF6 | 0.5297961 | 2.87E-14 |
| C1R | 0.684590967 | 5.93E-26 |
| C1S | 0.721134961 | 7.32E-30 |
| C1orf190 | 0.583067046 | 1.35E-17 |
| C1orf38 | 0.593282554 | 2.63E-18 |
| C21orf91 | 0.582784662 | 1.41E-17 |
| C3AR1 | 0.532392453 | 2.03E-14 |
| C3orf58 | 0.541277938 | 6.16E-15 |
| C5AR1 | 0.593147363 | 2.69E-18 |
| C5orf13 | 0.618300286 | 3.73E-20 |
| C5orf23 | 0.50143091 | 1.01E-12 |
| C5orf58 | 0.568563248 | 1.25E-16 |
| C5orf62 | 0.607576299 | 2.42E-19 |
| C6orf204 | 0.523851964 | 6.21E-14 |
| C6orf97 | 0.500132625 | 1.18E-12 |
| C7orf10 | 0.548460135 | 2.28E-15 |
| C7orf58 | 0.616297126 | 5.32E-20 |
| C9orf110 | 0.604876265 | 3.84E-19 |
| CALD1 | 0.720201537 | 9.38E-30 |
| CALHM2 | 0.532938208 | 1.89E-14 |
| CALU | 0.598953277 | 1.04E-18 |
| CAPZA1 | 0.539577898 | 7.76E-15 |
| CASS4 | 0.648525495 | 1.28E-22 |
| CBLB | 0.557553855 | 6.28E-16 |
| CBL | 0.588375881 | 5.81E-18 |
| CCDC102B | 0.536302174 | 1.21E-14 |
| CCDC36 | 0.561699091 | 3.44E-16 |
| CCDC50 | 0.507283977 | 4.97E-13 |
| CCDC80 | 0.705543891 | 4.03E-28 |
| CCDC88A | 0.512168808 | 2.72E-13 |
| CCDC8 | 0.550659134 | 1.68E-15 |
| CCL11 | 0.521508008 | 8.40E-14 |
| CCL13 | 0.50187761 | 9.56E-13 |
| CCL18 | 0.513726175 | 2.24E-13 |
| CCL22 | 0.514383144 | 2.07E-13 |
| CCL4 | 0.506990691 | 5.15E-13 |
| CCL5 | 0.505930392 | 5.86E-13 |
| CCR1 | 0.626905534 | 7.90E-21 |
| CCR2 | 0.504531245 | 6.94E-13 |
| CCR4 | 0.600310353 | 8.27E-19 |
| CCR5 | 0.570513881 | 9.30E-17 |
| CCR8 | 0.631337932 | 3.48E-21 |
| CD109 | 0.582613953 | 1.45E-17 |
| CD163 | 0.523153738 | 6.80E-14 |
| CD180 | 0.539568553 | 7.77E-15 |
| CD200R1 | 0.524591785 | 5.65E-14 |
| CD226 | 0.565497712 | 1.97E-16 |
| CD248 | 0.664631495 | 4.73E-24 |
| CD274 | 0.579995193 | 2.18E-17 |
| CD27 | 0.506246001 | 5.64E-13 |
| CD28 | 0.601372571 | 6.93E-19 |
| CD2 | 0.51694212 | 1.50E-13 |
| CD300A | 0.571330687 | 8.22E-17 |
| CD300C | 0.548272358 | 2.34E-15 |
| CD300E | 0.51362022 | 2.27E-13 |
| CD300LF | 0.521396164 | 8.52E-14 |
| CD33 | 0.539569133 | 7.77E-15 |
| CD37 | 0.561058427 | 3.78E-16 |
| CD38 | 0.584950653 | 1.00E-17 |
| CD3E | 0.535857803 | 1.28E-14 |
| CD3G | 0.56425448 | 2.36E-16 |
| CD40 | 0.517984367 | 1.31E-13 |
| CD4 | 0.637311704 | 1.13E-21 |
| CD53 | 0.641815667 | 4.76E-22 |
| CD68 | 0.549997627 | 1.84E-15 |
| CD69 | 0.634881121 | 1.79E-21 |
| CD80 | 0.711661007 | 8.63E-29 |
| CD83 | 0.500786708 | 1.09E-12 |
| CD84 | 0.625608788 | 1.00E-20 |
| CD86 | 0.664451806 | 4.91E-24 |
| CD93 | 0.585716647 | 8.88E-18 |
| CD96 | 0.59639915 | 1.58E-18 |
| CDH11 | 0.734828119 | 1.72E-31 |
| CDK15 | 0.576212919 | 3.91E-17 |
| CDK17 | 0.524097762 | 6.02E-14 |
| CDYL2 | 0.526861554 | 4.21E-14 |
| CEACAM21 | 0.524716761 | 5.56E-14 |
| CELF2 | 0.503199352 | 8.15E-13 |
| CEP170 | 0.609619169 | 1.71E-19 |
| CERCAM | 0.501678902 | 9.79E-13 |
| CFH | 0.607122686 | 2.62E-19 |
| CGGBP1 | 0.533930727 | 1.66E-14 |
| CH25H | 0.510285257 | 3.44E-13 |
| CHRD | 0.537203384 | 1.07E-14 |
| CHST11 | 0.650020871 | 9.49E-23 |
| CHSY1 | 0.752575685 | 9.26E-34 |
| CHSY3 | 0.735183751 | 1.55E-31 |
| CIITA | 0.575348821 | 4.47E-17 |
| CILP2 | 0.562000508 | 3.29E-16 |
| CILP | 0.578152478 | 2.90E-17 |
| CLDN11 | 0.528630527 | 3.34E-14 |
| CLEC2B | 0.552058689 | 1.38E-15 |
| CLEC4A | 0.560547801 | 4.07E-16 |
| CLEC5A | 0.59201588 | 3.23E-18 |
| CLEC7A | 0.647586497 | 1.54E-22 |
| CLIC2 | 0.58730078 | 6.90E-18 |
| CLIC4 | 0.6801336 | 1.62E-25 |
| CMAH | 0.622408681 | 1.79E-20 |
| CMTM3 | 0.500895375 | 1.08E-12 |
| CNN3 | 0.573720873 | 5.72E-17 |
| CNTNAP1 | 0.538783453 | 8.64E-15 |
| COL10A1 | 0.665956842 | 3.57E-24 |
| COL11A1 | 0.609629384 | 1.70E-19 |
| COL12A1 | 0.702723242 | 8.09E-28 |
| COL13A1 | 0.517315356 | 1.43E-13 |
| COL15A1 | 0.635465179 | 1.60E-21 |
| COL1A1 | 0.697014834 | 3.24E-27 |
| COL1A2 | 0.736078889 | 1.21E-31 |
| COL24A1 | 0.534460571 | 1.55E-14 |
| COL3A1 | 0.760538131 | 7.68E-35 |
| COL4A1 | 0.69062142 | 1.47E-26 |
| COL4A2 | 0.660888056 | 1.04E-23 |
| COL5A1 | 0.70855529 | 1.90E-28 |
| COL5A2 | 0.756937434 | 2.40E-34 |
| COL5A3 | 0.537519501 | 1.03E-14 |
| COL6A1 | 0.58468799 | 1.04E-17 |
| COL6A2 | 0.603836444 | 4.58E-19 |
| COL6A3 | 0.787314671 | 8.23E-39 |
| COL8A1 | 0.707043853 | 2.77E-28 |
| COL8A2 | 0.688836659 | 2.23E-26 |
| COLEC12 | 0.70043782 | 1.42E-27 |
| COPZ2 | 0.509910353 | 3.60E-13 |
| CORIN | 0.560576963 | 4.05E-16 |
| CORO1C | 0.604275387 | 4.25E-19 |
| CPA3 | 0.515125384 | 1.88E-13 |
| CPXM1 | 0.629588106 | 4.82E-21 |
| CPXM2 | 0.608985617 | 1.90E-19 |
| CPZ | 0.640065758 | 6.67E-22 |
| CR1 | 0.574286277 | 5.25E-17 |
| CRISPLD1 | 0.543870817 | 4.31E-15 |
| CRISPLD2 | 0.660616343 | 1.10E-23 |
| CRLF3 | 0.61250055 | 1.04E-19 |
| CRTAM | 0.578465886 | 2.76E-17 |
| CRTC3 | 0.500649632 | 1.11E-12 |
| CSF1R | 0.511729667 | 2.88E-13 |
| CSF1 | 0.537619027 | 1.01E-14 |
| CSF2RA | 0.60910439 | 1.86E-19 |
| CSF2RB | 0.638615165 | 8.81E-22 |
| CSF3R | 0.545266168 | 3.56E-15 |
| CSGALNACT2 | 0.73303431 | 2.85E-31 |
| CSRP2 | 0.5067889 | 5.28E-13 |
| CST7 | 0.63920087 | 7.88E-22 |
| CTDSPL2 | 0.569533648 | 1.08E-16 |
| CTGF | 0.653300361 | 4.91E-23 |
| CTHRC1 | 0.689356933 | 1.98E-26 |
| CTLA4 | 0.592868371 | 2.82E-18 |
| CTSC | 0.511914665 | 2.81E-13 |
| CTSK | 0.746583745 | 5.67E-33 |
| CTSO | 0.500241423 | 1.16E-12 |
| CTTNBP2NL | 0.531844672 | 2.19E-14 |
| CXCL10 | 0.541132116 | 6.28E-15 |
| CXCR4 | 0.559454239 | 4.77E-16 |
| CXCR6 | 0.515075803 | 1.90E-13 |
| CXCR7 | 0.596518427 | 1.55E-18 |
| CXorf21 | 0.557194157 | 6.61E-16 |
| CYBB | 0.618360112 | 3.69E-20 |
| CYBRD1 | 0.678695016 | 2.24E-25 |
| CYP1B1 | 0.605706245 | 3.33E-19 |
| CYR61 | 0.63405565 | 2.09E-21 |
| CYTH3 | 0.582136199 | 1.56E-17 |
| CYTH4 | 0.548946071 | 2.13E-15 |
| CYTIP | 0.656345173 | 2.64E-23 |
| DAAM2 | 0.508934518 | 4.06E-13 |
| DAB2 | 0.655880913 | 2.91E-23 |
| DACT1 | 0.63673504 | 1.26E-21 |
| DACT3 | 0.529135335 | 3.13E-14 |
| DAPP1 | 0.576951807 | 3.49E-17 |
| DCHS1 | 0.609160574 | 1.85E-19 |
| DCN | 0.741388207 | 2.62E-32 |
| DCP2 | 0.558373913 | 5.58E-16 |
| DDR2 | 0.709401218 | 1.53E-28 |
| DENND5A | 0.561302465 | 3.64E-16 |
| DFNA5 | 0.582412248 | 1.49E-17 |
| DGKI | 0.511680088 | 2.89E-13 |
| DIO2 | 0.69811911 | 2.48E-27 |
| DKK3 | 0.52173536 | 8.16E-14 |
| DLC1 | 0.540736468 | 6.63E-15 |
| DNAJB4 | 0.536550551 | 1.17E-14 |
| DNAJC5B | 0.512861096 | 2.50E-13 |
| DOCK10 | 0.517931833 | 1.32E-13 |
| DOCK11 | 0.646323708 | 1.97E-22 |
| DOCK2 | 0.685389725 | 4.94E-26 |
| DOK2 | 0.503769008 | 7.61E-13 |
| DOK3 | 0.530636541 | 2.57E-14 |
| DOK5 | 0.609131249 | 1.86E-19 |
| DPEP2 | 0.516407401 | 1.60E-13 |
| DPYD | 0.514075089 | 2.15E-13 |
| DPYSL3 | 0.591949279 | 3.27E-18 |
| DR1 | 0.522191038 | 7.69E-14 |
| DRAM1 | 0.569850787 | 1.03E-16 |
| DSE | 0.601303099 | 7.01E-19 |
| DUSP1 | 0.538051798 | 9.54E-15 |
| DUSP5 | 0.580734977 | 1.94E-17 |
| DYSF | 0.510109823 | 3.51E-13 |
| E2F3 | 0.542145358 | 5.47E-15 |
| EBF3 | 0.518168206 | 1.28E-13 |
| ECM2 | 0.733056726 | 2.83E-31 |
| EDEM1 | 0.511860637 | 2.83E-13 |
| EDIL3 | 0.520350136 | 9.73E-14 |
| EDNRA | 0.756021283 | 3.19E-34 |
| EFEMP1 | 0.624516609 | 1.22E-20 |
| EFEMP2 | 0.630390478 | 4.15E-21 |
| EFS | 0.608841961 | 1.95E-19 |
| EGFL6 | 0.607012592 | 2.67E-19 |
| EGR2 | 0.65441676 | 3.92E-23 |
| EHD3 | 0.529532487 | 2.97E-14 |
| ELK3 | 0.667635177 | 2.50E-24 |
| ELN | 0.527586376 | 3.83E-14 |
| ELTD1 | 0.501055724 | 1.05E-12 |
| EMILIN1 | 0.633981758 | 2.12E-21 |
| EMP1 | 0.596795884 | 1.48E-18 |
| EMR2 | 0.589183642 | 5.11E-18 |
| ENOX1 | 0.524166249 | 5.97E-14 |
| ENPEP | 0.609618876 | 1.71E-19 |
| ENTPD1 | 0.673475443 | 7.09E-25 |
| ENTPD7 | 0.519535009 | 1.08E-13 |
| EPB41L2 | 0.572581356 | 6.81E-17 |
| EPHA3 | 0.511189265 | 3.07E-13 |
| EPSTI1 | 0.540794056 | 6.58E-15 |
| EPYC | 0.530892121 | 2.48E-14 |
| ERBB2IP | 0.50109792 | 1.05E-12 |
| ERMN | 0.531938663 | 2.16E-14 |
| ETS1 | 0.686802658 | 3.57E-26 |
| EVC2 | 0.545190601 | 3.60E-15 |
| EVC | 0.643268616 | 3.59E-22 |
| EVI2A | 0.689516058 | 1.91E-26 |
| EVI2B | 0.661599786 | 8.94E-24 |
| F13A1 | 0.606386132 | 2.97E-19 |
| F2R | 0.682353293 | 9.85E-26 |
| FAM101B | 0.519491926 | 1.09E-13 |
| FAM102B | 0.551679008 | 1.45E-15 |
| FAM126A | 0.528955614 | 3.20E-14 |
| FAM151B | 0.510113367 | 3.51E-13 |
| FAM180A | 0.657259376 | 2.19E-23 |
| FAM190B | 0.511144631 | 3.09E-13 |
| FAM196B | 0.551294641 | 1.53E-15 |
| FAM198A | 0.530210924 | 2.71E-14 |
| FAM198B | 0.706671286 | 3.04E-28 |
| FAM26E | 0.715950511 | 2.86E-29 |
| FAM38B | 0.589151404 | 5.13E-18 |
| FAM49A | 0.717654683 | 1.83E-29 |
| FAM63B | 0.503192026 | 8.16E-13 |
| FAM65B | 0.509530052 | 3.77E-13 |
| FAP | 0.723344876 | 4.06E-30 |
| FAS | 0.550342447 | 1.75E-15 |
| FAT3 | 0.550255024 | 1.77E-15 |
| FAT4 | 0.604279332 | 4.25E-19 |
| FBLN1 | 0.650293071 | 8.99E-23 |
| FBLN2 | 0.696572161 | 3.60E-27 |
| FBN1 | 0.765960088 | 1.33E-35 |
| FBN2 | 0.564988948 | 2.12E-16 |
| FBXL7 | 0.657682956 | 2.01E-23 |
| FCAR | 0.52651812 | 4.40E-14 |
| FCER1G | 0.581350401 | 1.77E-17 |
| FCGR2A | 0.622179538 | 1.87E-20 |
| FCGR2B | 0.688520972 | 2.40E-26 |
| FCGR2C | 0.718928946 | 1.31E-29 |
| FCGR3A | 0.559483406 | 4.75E-16 |
| FCHSD2 | 0.663634335 | 5.84E-24 |
| FCRL5 | 0.523590734 | 6.43E-14 |
| FERMT2 | 0.637985935 | 9.94E-22 |
| FEZ1 | 0.577959358 | 2.99E-17 |
| FGD2 | 0.523588256 | 6.43E-14 |
| FGF7 | 0.632515619 | 2.79E-21 |
| FGL2 | 0.538576597 | 8.89E-15 |
| FGR | 0.532130701 | 2.11E-14 |
| FIBIN | 0.688766898 | 2.27E-26 |
| FILIP1L | 0.658297784 | 1.77E-23 |
| FILIP1 | 0.513833775 | 2.21E-13 |
| FKBP14 | 0.668625349 | 2.02E-24 |
| FKBP7 | 0.622101598 | 1.89E-20 |
| FLI1 | 0.618583616 | 3.55E-20 |
| FLJ22536 | 0.595707105 | 1.77E-18 |
| FLJ36031 | 0.517701224 | 1.36E-13 |
| FLJ42709 | 0.507248911 | 4.99E-13 |
| FLNA | 0.613260995 | 9.07E-20 |
| FLNC | 0.544889052 | 3.75E-15 |
| FMNL1 | 0.54322604 | 4.71E-15 |
| FMNL3 | 0.718998052 | 1.29E-29 |
| FMO1 | 0.617403949 | 4.38E-20 |
| FN1 | 0.6908973 | 1.38E-26 |
| FNBP1 | 0.567953577 | 1.37E-16 |
| FNDC1 | 0.697442338 | 2.92E-27 |
| FNDC3B | 0.553391251 | 1.14E-15 |
| FOSL2 | 0.644845188 | 2.64E-22 |
| FOXF1 | 0.557327071 | 6.49E-16 |
| FOXF2 | 0.51636078 | 1.61E-13 |
| FOXN2 | 0.513985844 | 2.17E-13 |
| FOXN3 | 0.534621371 | 1.51E-14 |
| FOXO1 | 0.513259863 | 2.38E-13 |
| FOXP3 | 0.648063195 | 1.40E-22 |
| FPR1 | 0.530726842 | 2.54E-14 |
| FPR2 | 0.512942066 | 2.47E-13 |
| FPR3 | 0.679475974 | 1.88E-25 |
| FRMD6 | 0.706771157 | 2.97E-28 |
| FSTL1 | 0.760721235 | 7.24E-35 |
| FUT11 | 0.537669523 | 1.00E-14 |
| FYB | 0.584894201 | 1.01E-17 |
| FYN | 0.525054512 | 5.32E-14 |
| FZD1 | 0.592276246 | 3.10E-18 |
| FZD7 | 0.559916269 | 4.46E-16 |
| GAB3 | 0.545357952 | 3.51E-15 |
| GAL3ST4 | 0.552800521 | 1.24E-15 |
| GALNTL2 | 0.542951305 | 4.90E-15 |
| GAS1 | 0.7035194 | 6.65E-28 |
| GAS7 | 0.603874182 | 4.55E-19 |
| GBP1 | 0.696301771 | 3.84E-27 |
| GBP4 | 0.571566343 | 7.94E-17 |
| GBP5 | 0.658378793 | 1.74E-23 |
| GDF6 | 0.596891436 | 1.46E-18 |
| GEFT | 0.51782318 | 1.34E-13 |
| GEM | 0.713929755 | 4.83E-29 |
| GFPT2 | 0.692482639 | 9.51E-27 |
| GGT5 | 0.584381792 | 1.10E-17 |
| GIMAP4 | 0.507830308 | 4.65E-13 |
| GIT2 | 0.514130508 | 2.13E-13 |
| GJA1 | 0.58728524 | 6.92E-18 |
| GJD3 | 0.612977801 | 9.53E-20 |
| GLI1 | 0.639078015 | 8.07E-22 |
| GLI2 | 0.622466804 | 1.77E-20 |
| GLI3 | 0.699686666 | 1.70E-27 |
| GLIPR1 | 0.735369111 | 1.48E-31 |
| GLIPR2 | 0.597925463 | 1.23E-18 |
| GLIS2 | 0.50176106 | 9.69E-13 |
| GLT8D2 | 0.696768084 | 3.43E-27 |
| GM2A | 0.510771299 | 3.24E-13 |
| GNA13 | 0.581404284 | 1.75E-17 |
| GNB4 | 0.725122998 | 2.51E-30 |
| GNPTAB | 0.582856208 | 1.39E-17 |
| GPNMB | 0.682582859 | 9.36E-26 |
| GPR116 | 0.561671739 | 3.45E-16 |
| GPR124 | 0.603059324 | 5.22E-19 |
| GPR132 | 0.505670184 | 6.05E-13 |
| GPR141 | 0.555421179 | 8.53E-16 |
| GPR161 | 0.602648547 | 5.59E-19 |
| GPR171 | 0.578699013 | 2.67E-17 |
| GPR174 | 0.516527685 | 1.58E-13 |
| GPR176 | 0.678870511 | 2.15E-25 |
| GPR183 | 0.69629303 | 3.85E-27 |
| GPR1 | 0.52442261 | 5.77E-14 |
| GPR65 | 0.545602164 | 3.40E-15 |
| GPR68 | 0.620235359 | 2.64E-20 |
| GPR84 | 0.538591762 | 8.87E-15 |
| GPR85 | 0.514414312 | 2.06E-13 |
| GPX8 | 0.6442534 | 2.96E-22 |
| GREM1 | 0.683501117 | 7.60E-26 |
| GSTM5 | 0.543133128 | 4.78E-15 |
| GTDC1 | 0.561512866 | 3.53E-16 |
| GUCY1A3 | 0.530933871 | 2.47E-14 |
| GVIN1 | 0.590181567 | 4.35E-18 |
| GXYLT2 | 0.680718127 | 1.42E-25 |
| HAPLN3 | 0.501939616 | 9.49E-13 |
| HAS2 | 0.654633467 | 3.75E-23 |
| HAVCR2 | 0.601449082 | 6.84E-19 |
| HCK | 0.546857478 | 2.85E-15 |
| HCLS1 | 0.537752085 | 9.94E-15 |
| HDAC9 | 0.502394475 | 8.98E-13 |
| HECA | 0.603402644 | 4.92E-19 |
| HEG1 | 0.641351805 | 5.21E-22 |
| HELB | 0.525552732 | 4.99E-14 |
| HGF | 0.57895808 | 2.56E-17 |
| HHIPL1 | 0.622943773 | 1.62E-20 |
| HIF1A | 0.555693557 | 8.20E-16 |
| HIP1 | 0.624801956 | 1.16E-20 |
| HIVEP1 | 0.580154283 | 2.13E-17 |
| HIVEP2 | 0.617144774 | 4.58E-20 |
| HK3 | 0.511640987 | 2.91E-13 |
| HLA-DMB | 0.560070939 | 4.36E-16 |
| HLA-DOA | 0.547124624 | 2.75E-15 |
| HLA-DPA1 | 0.519409319 | 1.10E-13 |
| HLA-DQB2 | 0.561146818 | 3.73E-16 |
| HLA-DRA | 0.587484726 | 6.70E-18 |
| HLX | 0.502353116 | 9.03E-13 |
| HMCN1 | 0.703463968 | 6.75E-28 |
| HMOX1 | 0.589153374 | 5.13E-18 |
| HOOK3 | 0.557902076 | 5.97E-16 |
| HOXD8 | 0.573348833 | 6.06E-17 |
| HPS5 | 0.597277834 | 1.37E-18 |
| HRH2 | 0.526550218 | 4.38E-14 |
| HSD11B1 | 0.579180514 | 2.47E-17 |
| HSD17B6 | 0.597767995 | 1.26E-18 |
| HSPG2 | 0.623766441 | 1.40E-20 |
| HTRA1 | 0.529973946 | 2.80E-14 |
| HTRA3 | 0.600963748 | 7.42E-19 |
| HVCN1 | 0.567567413 | 1.45E-16 |
| ICAM1 | 0.593797595 | 2.42E-18 |
| ICOS | 0.658892536 | 1.57E-23 |
| IFI16 | 0.721404251 | 6.82E-30 |
| IFNGR1 | 0.57869727 | 2.67E-17 |
| IGDCC4 | 0.693166035 | 8.09E-27 |
| IGFBP5 | 0.585700895 | 8.90E-18 |
| IGFBP7 | 0.586151328 | 8.29E-18 |
| IGSF6 | 0.569278919 | 1.12E-16 |
| IKBIP | 0.537692033 | 1.00E-14 |
| IKZF1 | 0.553196446 | 1.17E-15 |
| IKZF3 | 0.554892253 | 9.20E-16 |
| IL10RA | 0.599765871 | 9.06E-19 |
| IL16 | 0.6272792 | 7.38E-21 |
| IL1R1 | 0.688216969 | 2.58E-26 |
| IL1RAP | 0.529494422 | 2.98E-14 |
| IL21R | 0.680546669 | 1.48E-25 |
| IL2RA | 0.581762836 | 1.66E-17 |
| IL2RB | 0.625171308 | 1.08E-20 |
| IL7R | 0.700105808 | 1.53E-27 |
| IL8 | 0.559605429 | 4.67E-16 |
| IL9R | 0.535349058 | 1.37E-14 |
| INHBA | 0.74396998 | 1.23E-32 |
| IRAK3 | 0.567903131 | 1.38E-16 |
| IRF4 | 0.581757799 | 1.66E-17 |
| ISLR | 0.628320267 | 6.09E-21 |
| ISM1 | 0.664496754 | 4.87E-24 |
| ITGA11 | 0.666476149 | 3.20E-24 |
| ITGA1 | 0.670675846 | 1.30E-24 |
| ITGA4 | 0.674800018 | 5.30E-25 |
| ITGA5 | 0.591139984 | 3.73E-18 |
| ITGA9 | 0.542458549 | 5.24E-15 |
| ITGAL | 0.56008343 | 4.35E-16 |
| ITGAM | 0.562497031 | 3.06E-16 |
| ITGAV | 0.545813944 | 3.30E-15 |
| ITGAX | 0.643069646 | 3.73E-22 |
| ITGB1 | 0.519804064 | 1.04E-13 |
| ITGB2 | 0.642508506 | 4.16E-22 |
| ITGB7 | 0.552128556 | 1.36E-15 |
| ITGBL1 | 0.537686181 | 1.00E-14 |
| ITK | 0.614698367 | 7.05E-20 |
| ITPRIP | 0.72875595 | 9.34E-31 |
| ITSN1 | 0.551246744 | 1.54E-15 |
| JAK1 | 0.536801549 | 1.13E-14 |
| JAM3 | 0.50820347 | 4.44E-13 |
| JMJD1C | 0.529948753 | 2.81E-14 |
| KAL1 | 0.588979918 | 5.28E-18 |
| KCNA3 | 0.509115969 | 3.97E-13 |
| KCND2 | 0.657073952 | 2.28E-23 |
| KCNE4 | 0.607284886 | 2.55E-19 |
| KCTD10 | 0.589960914 | 4.51E-18 |
| KCTD20 | 0.62018851 | 2.67E-20 |
| KDELC1 | 0.685048205 | 5.34E-26 |
| KDELC2 | 0.54884728 | 2.16E-15 |
| KERA | 0.670605078 | 1.32E-24 |
| KIAA0247 | 0.587310823 | 6.89E-18 |
| KIAA0355 | 0.530855421 | 2.49E-14 |
| KIAA0748 | 0.56607023 | 1.81E-16 |
| KIAA1033 | 0.576909768 | 3.51E-17 |
| KIAA1217 | 0.520596196 | 9.43E-14 |
| KIAA1432 | 0.676441238 | 3.69E-25 |
| KIAA1755 | 0.58101619 | 1.86E-17 |
| KIAA1949 | 0.675432385 | 4.61E-25 |
| KIF26B | 0.595976436 | 1.69E-18 |
| KIRREL | 0.650379216 | 8.83E-23 |
| KLF12 | 0.603662543 | 4.71E-19 |
| KLF6 | 0.561191351 | 3.70E-16 |
| KLF7 | 0.597982342 | 1.22E-18 |
| KLF8 | 0.628131722 | 6.31E-21 |
| KLHL20 | 0.545376879 | 3.50E-15 |
| KLHL4 | 0.53259772 | 1.98E-14 |
| KLHL5 | 0.512982495 | 2.46E-13 |
| KLHL6 | 0.621529954 | 2.10E-20 |
| KLRD1 | 0.586731146 | 7.56E-18 |
| KMO | 0.54659498 | 2.96E-15 |
| L3MBTL3 | 0.629030411 | 5.34E-21 |
| LAIR1 | 0.581037466 | 1.85E-17 |
| LAMA2 | 0.63369817 | 2.24E-21 |
| LAMA4 | 0.718584625 | 1.44E-29 |
| LAMB1 | 0.552286972 | 1.33E-15 |
| LAMC1 | 0.610336448 | 1.51E-19 |
| LAMP3 | 0.545478471 | 3.46E-15 |
| LAPTM5 | 0.590893605 | 3.88E-18 |
| LATS2 | 0.6880806 | 2.66E-26 |
| LAX1 | 0.533518017 | 1.75E-14 |
| LAYN | 0.654721989 | 3.68E-23 |
| LBH | 0.548311129 | 2.33E-15 |
| LCP1 | 0.530833493 | 2.50E-14 |
| LCP2 | 0.677848624 | 2.70E-25 |
| LDB2 | 0.504726052 | 6.78E-13 |
| LEF1 | 0.738590403 | 5.89E-32 |
| LGALS1 | 0.531638567 | 2.25E-14 |
| LHFPL2 | 0.579481494 | 2.36E-17 |
| LHFP | 0.611688509 | 1.19E-19 |
| LILRA5 | 0.507785337 | 4.67E-13 |
| LILRA6 | 0.596404297 | 1.58E-18 |
| LILRB1 | 0.567427077 | 1.48E-16 |
| LILRB2 | 0.586311787 | 8.08E-18 |
| LILRB3 | 0.521854384 | 8.03E-14 |
| LILRB4 | 0.572428682 | 6.97E-17 |
| LILRB5 | 0.50702484 | 5.13E-13 |
| LIMS1 | 0.619841197 | 2.84E-20 |
| LIN7A | 0.527129492 | 4.06E-14 |
| LMCD1 | 0.554826238 | 9.28E-16 |
| LOC100192378 | 0.520083675 | 1.01E-13 |
| LOC257358 | 0.550939147 | 1.61E-15 |
| LOC285548 | 0.573159916 | 6.23E-17 |
| LOC339524 | 0.503040074 | 8.31E-13 |
| LOC374443 | 0.511495066 | 2.96E-13 |
| LOC399959 | 0.536161904 | 1.23E-14 |
| LOC400759 | 0.523531896 | 6.48E-14 |
| LOC90586 | 0.508278213 | 4.40E-13 |
| LOH3CR2A | 0.536114528 | 1.24E-14 |
| LOXL2 | 0.564939246 | 2.14E-16 |
| LOXL3 | 0.703027837 | 7.51E-28 |
| LOX | 0.705283624 | 4.30E-28 |
| LPAR1 | 0.566199866 | 1.77E-16 |
| LPAR4 | 0.648424274 | 1.30E-22 |
| LPHN2 | 0.512112487 | 2.74E-13 |
| LPXN | 0.568215161 | 1.31E-16 |
| LRP12 | 0.514044129 | 2.16E-13 |
| LRP1 | 0.680505099 | 1.49E-25 |
| LRRC15 | 0.664521657 | 4.84E-24 |
| LRRC17 | 0.509279135 | 3.89E-13 |
| LRRC25 | 0.540410701 | 6.93E-15 |
| LRRC32 | 0.612946306 | 9.59E-20 |
| LRRC40 | 0.500786953 | 1.09E-12 |
| LRRC58 | 0.537941435 | 9.68E-15 |
| LRRC8C | 0.636771343 | 1.25E-21 |
| LRRK1 | 0.556426062 | 7.38E-16 |
| LRRK2 | 0.577330227 | 3.29E-17 |
| LRRN4CL | 0.558206372 | 5.71E-16 |
| LSAMP | 0.516213945 | 1.64E-13 |
| LSP1 | 0.648737291 | 1.23E-22 |
| LST1 | 0.503542482 | 7.82E-13 |
| LTBP1 | 0.684061278 | 6.69E-26 |
| LTBP2 | 0.697408689 | 2.95E-27 |
| LUM | 0.769555729 | 4.06E-36 |
| LY86 | 0.509743993 | 3.67E-13 |
| LY96 | 0.594836108 | 2.04E-18 |
| LY9 | 0.500278838 | 1.16E-12 |
| LZTS1 | 0.52807778 | 3.59E-14 |
| MACF1 | 0.508513576 | 4.27E-13 |
| MAF | 0.643960057 | 3.14E-22 |
| MALT1 | 0.605610141 | 3.39E-19 |
| MAML2 | 0.563294339 | 2.72E-16 |
| MAP3K2 | 0.503778834 | 7.60E-13 |
| MAP3K3 | 0.509440115 | 3.81E-13 |
| MAP3K8 | 0.702175717 | 9.26E-28 |
| MAP4K5 | 0.589147141 | 5.14E-18 |
| 1-Mar | 0.605048132 | 3.73E-19 |
| MARVELD1 | 0.512140831 | 2.73E-13 |
| MATN3 | 0.510376495 | 3.40E-13 |
| MBNL1 | 0.657867108 | 1.94E-23 |
| MCC | 0.512716775 | 2.54E-13 |
| MDFIC | 0.63092686 | 3.76E-21 |
| MEF2A | 0.510598738 | 3.31E-13 |
| MEF2C | 0.618648244 | 3.51E-20 |
| MEFV | 0.527526358 | 3.86E-14 |
| MEOX2 | 0.543197665 | 4.73E-15 |
| MEX3B | 0.650669462 | 8.33E-23 |
| MFAP2 | 0.580558611 | 2.00E-17 |
| MFAP3 | 0.503576851 | 7.79E-13 |
| MFAP4 | 0.611545052 | 1.22E-19 |
| MFAP5 | 0.668361003 | 2.14E-24 |
| MFGE8 | 0.524297013 | 5.87E-14 |
| MGC45800 | 0.547129497 | 2.75E-15 |
| MICAL2 | 0.50655008 | 5.43E-13 |
| MICB | 0.52682995 | 4.23E-14 |
| MIER1 | 0.503721885 | 7.66E-13 |
| MIR155HG | 0.534790932 | 1.48E-14 |
| MITF | 0.617792338 | 4.09E-20 |
| MMD | 0.544957699 | 3.71E-15 |
| MMP11 | 0.507320986 | 4.95E-13 |
| MMP14 | 0.605623593 | 3.38E-19 |
| MMP16 | 0.529041301 | 3.17E-14 |
| MMP19 | 0.538500621 | 8.98E-15 |
| MMP2 | 0.702165101 | 9.28E-28 |
| MN1 | 0.51327622 | 2.37E-13 |
| MNDA | 0.61417216 | 7.74E-20 |
| MOBKL1B | 0.556446433 | 7.36E-16 |
| MORC3 | 0.509014198 | 4.02E-13 |
| MOXD1 | 0.658329089 | 1.76E-23 |
| MPDZ | 0.506656761 | 5.36E-13 |
| MPEG1 | 0.567433786 | 1.48E-16 |
| MRAS | 0.577538038 | 3.19E-17 |
| MRC2 | 0.692898877 | 8.62E-27 |
| MRVI1 | 0.666982575 | 2.87E-24 |
| MS4A2 | 0.51967003 | 1.06E-13 |
| MS4A4A | 0.531386808 | 2.32E-14 |
| MS4A6A | 0.542512827 | 5.20E-15 |
| MS4A7 | 0.524209536 | 5.93E-14 |
| MSC | 0.603121494 | 5.16E-19 |
| MSN | 0.658444423 | 1.72E-23 |
| MSR1 | 0.617053605 | 4.66E-20 |
| MSRB3 | 0.688807186 | 2.25E-26 |
| MTMR2 | 0.517444349 | 1.41E-13 |
| MXRA5 | 0.65248491 | 5.79E-23 |
| MXRA8 | 0.596130185 | 1.65E-18 |
| MYH9 | 0.632104732 | 3.02E-21 |
| MYL3 | 0.504044668 | 7.36E-13 |
| MYLK | 0.617474169 | 4.32E-20 |
| MYO1F | 0.524811369 | 5.49E-14 |
| MYO1G | 0.635997717 | 1.45E-21 |
| MYO5A | 0.513547149 | 2.29E-13 |
| MYO9B | 0.588208117 | 5.97E-18 |
| MYOF | 0.516090421 | 1.67E-13 |
| NAALADL1 | 0.541255264 | 6.18E-15 |
| NAV3 | 0.576748836 | 3.60E-17 |
| NCAM2 | 0.555003577 | 9.05E-16 |
| NCF1B | 0.526073422 | 4.66E-14 |
| NCF1C | 0.512212476 | 2.71E-13 |
| NCF1 | 0.559725785 | 4.58E-16 |
| NCF2 | 0.616316795 | 5.31E-20 |
| NCF4 | 0.507816836 | 4.66E-13 |
| NCKAP1L | 0.619604188 | 2.96E-20 |
| NEDD1 | 0.536569983 | 1.16E-14 |
| NEDD4 | 0.620063833 | 2.73E-20 |
| NETO1 | 0.535814078 | 1.29E-14 |
| NEXN | 0.602354866 | 5.87E-19 |
| NID1 | 0.728513968 | 9.98E-31 |
| NID2 | 0.751696244 | 1.21E-33 |
| NIN | 0.532500036 | 2.01E-14 |
| NKX3-2 | 0.613549883 | 8.63E-20 |
| NLRC5 | 0.527222661 | 4.01E-14 |
| NLRP1 | 0.635304597 | 1.65E-21 |
| NLRP3 | 0.596165635 | 1.64E-18 |
| NOD1 | 0.534027867 | 1.64E-14 |
| NOD2 | 0.574895731 | 4.79E-17 |
| NOTCH2 | 0.566514764 | 1.69E-16 |
| NOTCH3 | 0.597025053 | 1.43E-18 |
| NOX4 | 0.634224626 | 2.03E-21 |
| NPL | 0.514386523 | 2.07E-13 |
| NPR2 | 0.58128002 | 1.78E-17 |
| NPR3 | 0.564278136 | 2.36E-16 |
| NR2F1 | 0.524308371 | 5.86E-14 |
| NR3C1 | 0.549511182 | 1.97E-15 |
| NRBF2 | 0.513901888 | 2.20E-13 |
| NRIP3 | 0.533943265 | 1.66E-14 |
| NRK | 0.626969605 | 7.81E-21 |
| NRP1 | 0.549776965 | 1.90E-15 |
| NRP2 | 0.553622674 | 1.10E-15 |
| NTF3 | 0.51016108 | 3.49E-13 |
| NTM | 0.653331092 | 4.88E-23 |
| NUAK1 | 0.572209359 | 7.20E-17 |
| OBFC2A | 0.58309338 | 1.34E-17 |
| ODZ3 | 0.633651015 | 2.26E-21 |
| ODZ4 | 0.512201089 | 2.71E-13 |
| OGFRL1 | 0.514133626 | 2.13E-13 |
| OLFML1 | 0.762860815 | 3.65E-35 |
| OLFML2B | 0.64267632 | 4.03E-22 |
| OLFML3 | 0.611059015 | 1.33E-19 |
| OLR1 | 0.592555625 | 2.96E-18 |
| OMD | 0.645836942 | 2.17E-22 |
| OSBPL11 | 0.591126127 | 3.74E-18 |
| OSBPL8 | 0.636997935 | 1.20E-21 |
| OSMR | 0.659136338 | 1.49E-23 |
| OSM | 0.538447346 | 9.04E-15 |
| OSTM1 | 0.583048185 | 1.35E-17 |
| P2RX7 | 0.501452913 | 1.01E-12 |
| P2RY10 | 0.584843334 | 1.02E-17 |
| P2RY14 | 0.52322497 | 6.74E-14 |
| P4HA3 | 0.655236838 | 3.32E-23 |
| PABPC4L | 0.713333602 | 5.63E-29 |
| PAG1 | 0.525880225 | 4.78E-14 |
| PALLD | 0.599217288 | 9.92E-19 |
| PALM2-AKAP2 | 0.634639759 | 1.88E-21 |
| PAMR1 | 0.527672492 | 3.79E-14 |
| PANX1 | 0.525226429 | 5.20E-14 |
| PAPPA | 0.693007563 | 8.40E-27 |
| PARD6G | 0.68812453 | 2.63E-26 |
| PARP14 | 0.550232075 | 1.78E-15 |
| PARP15 | 0.534028162 | 1.64E-14 |
| PARP8 | 0.520549177 | 9.49E-14 |
| PARVG | 0.563282301 | 2.73E-16 |
| PCDH18 | 0.615303393 | 6.34E-20 |
| PCDH19 | 0.51162973 | 2.91E-13 |
| PCDH7 | 0.593101291 | 2.71E-18 |
| PCDHGA12 | 0.581628024 | 1.69E-17 |
| PCOLCE | 0.654066135 | 4.21E-23 |
| PCSK5 | 0.651529488 | 7.01E-23 |
| PDCD1LG2 | 0.696032705 | 4.10E-27 |
| PDE1A | 0.529597301 | 2.94E-14 |
| PDE4B | 0.540469204 | 6.87E-15 |
| PDGFC | 0.623274462 | 1.53E-20 |
| PDGFRA | 0.686167902 | 4.13E-26 |
| PDGFRB | 0.769544353 | 4.08E-36 |
| PDGFRL | 0.559701221 | 4.60E-16 |
| PDLIM3 | 0.657350954 | 2.15E-23 |
| PDLIM5 | 0.555388162 | 8.57E-16 |
| PDPN | 0.66531543 | 4.09E-24 |
| PEA15 | 0.506225867 | 5.65E-13 |
| PHACTR2 | 0.54617393 | 3.14E-15 |
| PHF20 | 0.522510424 | 7.38E-14 |
| PHLDB1 | 0.570919717 | 8.75E-17 |
| PICALM | 0.651202135 | 7.49E-23 |
| PIK3CA | 0.575523435 | 4.35E-17 |
| PIK3CD | 0.582793953 | 1.41E-17 |
| PIK3CG | 0.639760951 | 7.08E-22 |
| PIK3R5 | 0.586669733 | 7.63E-18 |
| PIK3R6 | 0.62198435 | 1.93E-20 |
| PILRA | 0.594537948 | 2.15E-18 |
| PIM1 | 0.563079333 | 2.81E-16 |
| PIP4K2A | 0.615022806 | 6.66E-20 |
| PKD2 | 0.590514984 | 4.12E-18 |
| PLA2G7 | 0.570583086 | 9.21E-17 |
| PLA2R1 | 0.519634083 | 1.07E-13 |
| PLAU | 0.586566813 | 7.76E-18 |
| PLEKHG1 | 0.629391915 | 5.00E-21 |
| PLEKHG2 | 0.517063894 | 1.48E-13 |
| PLEKHO1 | 0.532159793 | 2.10E-14 |
| PLEKHO2 | 0.600278983 | 8.31E-19 |
| PLEK | 0.598600211 | 1.10E-18 |
| PLIN2 | 0.533015425 | 1.87E-14 |
| PLN | 0.526405524 | 4.47E-14 |
| PLOD2 | 0.611206626 | 1.30E-19 |
| PLS3 | 0.589892101 | 4.56E-18 |
| PLXDC1 | 0.587221108 | 6.99E-18 |
| PLXDC2 | 0.690166401 | 1.64E-26 |
| PLXNC1 | 0.53979071 | 7.54E-15 |
| PMP22 | 0.621188388 | 2.23E-20 |
| PNOC | 0.539028767 | 8.36E-15 |
| PODNL1 | 0.536063319 | 1.25E-14 |
| PODN | 0.598116224 | 1.19E-18 |
| POM121L9P | 0.52447608 | 5.73E-14 |
| POSTN | 0.65217472 | 6.16E-23 |
| PPAP2B | 0.551257228 | 1.54E-15 |
| PPAPDC1A | 0.606772919 | 2.78E-19 |
| PPFIA2 | 0.578481749 | 2.76E-17 |
| PPFIBP1 | 0.605557979 | 3.42E-19 |
| PPIL4 | 0.504273382 | 7.16E-13 |
| PPP1R12A | 0.503327668 | 8.03E-13 |
| PPP1R3B | 0.532304636 | 2.06E-14 |
| PPPDE1 | 0.547963047 | 2.45E-15 |
| PRDM1 | 1 | 1.00E-40 |
| PRDM2 | 0.563692624 | 2.57E-16 |
| PRDM6 | 0.66755676 | 2.54E-24 |
| PRELP | 0.577270868 | 3.32E-17 |
| PREX1 | 0.506418175 | 5.52E-13 |
| PRF1 | 0.538587627 | 8.87E-15 |
| PRICKLE1 | 0.605716228 | 3.33E-19 |
| PRICKLE2 | 0.506533746 | 5.44E-13 |
| PRKCH | 0.509936835 | 3.59E-13 |
| PRKD3 | 0.630263396 | 4.25E-21 |
| PRKG1 | 0.618841706 | 3.39E-20 |
| PRR16 | 0.753579986 | 6.80E-34 |
| PRRX1 | 0.747669523 | 4.10E-33 |
| PRSS23 | 0.65254782 | 5.72E-23 |
| PRTG | 0.546238635 | 3.11E-15 |
| PTAFR | 0.563242158 | 2.74E-16 |
| PTGDR | 0.581254465 | 1.79E-17 |
| PTGER3 | 0.587578164 | 6.60E-18 |
| PTGFRN | 0.509463092 | 3.80E-13 |
| PTGIR | 0.594984371 | 1.99E-18 |
| PTGS1 | 0.511733486 | 2.87E-13 |
| PTHLH | 0.510348013 | 3.41E-13 |
| PTPLAD2 | 0.708886742 | 1.75E-28 |
| PTPN22 | 0.652296703 | 6.01E-23 |
| PTPN7 | 0.51546329 | 1.81E-13 |
| PTPRC | 0.673721653 | 6.72E-25 |
| PTPRG | 0.563378713 | 2.69E-16 |
| PTPRO | 0.59602906 | 1.68E-18 |
| PTRF | 0.621135876 | 2.25E-20 |
| PXDN | 0.704931082 | 4.69E-28 |
| PXK | 0.502536331 | 8.83E-13 |
| PYGO1 | 0.584309312 | 1.11E-17 |
| QKI | 0.704561796 | 5.14E-28 |
| RAB23 | 0.60642055 | 2.95E-19 |
| RAB31 | 0.738423182 | 6.18E-32 |
| RAB3IL1 | 0.571974673 | 7.46E-17 |
| RAB42 | 0.522014608 | 7.87E-14 |
| RAB8B | 0.643001451 | 3.78E-22 |
| RAI14 | 0.677104352 | 3.19E-25 |
| RAP1A | 0.539915659 | 7.41E-15 |
| RAP2C | 0.598116293 | 1.19E-18 |
| RARB | 0.514446201 | 2.05E-13 |
| RASA2 | 0.555788465 | 8.09E-16 |
| RASAL2 | 0.51428333 | 2.09E-13 |
| RASGRF2 | 0.691970395 | 1.07E-26 |
| RASGRP3 | 0.571349873 | 8.20E-17 |
| RASGRP4 | 0.564880264 | 2.16E-16 |
| RASSF2 | 0.598260763 | 1.16E-18 |
| RASSF8 | 0.624095719 | 1.32E-20 |
| RBMS1 | 0.652499831 | 5.77E-23 |
| RBMS3 | 0.661622291 | 8.89E-24 |
| RCN3 | 0.526298508 | 4.53E-14 |
| RCSD1 | 0.563409115 | 2.68E-16 |
| RECK | 0.644788119 | 2.67E-22 |
| RECQL | 0.660186285 | 1.20E-23 |
| RELT | 0.587468626 | 6.72E-18 |
| REL | 0.520751247 | 9.25E-14 |
| RFTN1 | 0.715714218 | 3.04E-29 |
| RFTN2 | 0.57038955 | 9.48E-17 |
| RGS16 | 0.640730583 | 5.87E-22 |
| RGS1 | 0.652415238 | 5.87E-23 |
| RHOJ | 0.515966249 | 1.70E-13 |
| RHOQ | 0.527500148 | 3.87E-14 |
| RIN3 | 0.513148793 | 2.41E-13 |
| RND3 | 0.515370451 | 1.83E-13 |
| RNF122 | 0.525847922 | 4.80E-14 |
| RNF144A | 0.601575666 | 6.69E-19 |
| RNF169 | 0.51706793 | 1.48E-13 |
| RNF217 | 0.596098338 | 1.66E-18 |
| ROBO1 | 0.702142501 | 9.33E-28 |
| ROR2 | 0.521217667 | 8.71E-14 |
| RORA | 0.546060934 | 3.19E-15 |
| RP2 | 0.509173744 | 3.94E-13 |
| RRAGC | 0.539480579 | 7.86E-15 |
| RSAD2 | 0.546934741 | 2.82E-15 |
| RSPO3 | 0.5217026 | 8.19E-14 |
| RUNX1T1 | 0.504996226 | 6.56E-13 |
| RUNX1 | 0.598643155 | 1.09E-18 |
| RUNX2 | 0.627794335 | 6.71E-21 |
| RUNX3 | 0.551033686 | 1.59E-15 |
| S100PBP | 0.513615617 | 2.27E-13 |
| S1PR3 | 0.65298925 | 5.23E-23 |
| SACS | 0.584902511 | 1.01E-17 |
| SAMD4A | 0.540051575 | 7.28E-15 |
| SAMD9L | 0.568603682 | 1.24E-16 |
| SAMHD1 | 0.53786172 | 9.79E-15 |
| SAMSN1 | 0.705840034 | 3.74E-28 |
| SASH3 | 0.519375674 | 1.10E-13 |
| SAV1 | 0.529269749 | 3.07E-14 |
| SCARA3 | 0.502340203 | 9.04E-13 |
| SCUBE2 | 0.630191174 | 4.31E-21 |
| SEC23A | 0.658926018 | 1.56E-23 |
| SELL | 0.514031178 | 2.16E-13 |
| SELPLG | 0.501428902 | 1.01E-12 |
| SEMA3A | 0.505784502 | 5.96E-13 |
| SEMA3C | 0.555036891 | 9.01E-16 |
| SEMA3D | 0.643383272 | 3.51E-22 |
| SEPT11 | 0.648289835 | 1.34E-22 |
| SEPT7 | 0.556180939 | 7.65E-16 |
| SEPT8 | 0.513783211 | 2.23E-13 |
| SERPINB2 | 0.506913978 | 5.20E-13 |
| SERPINB9 | 0.605540245 | 3.43E-19 |
| SERPINE1 | 0.554539219 | 9.67E-16 |
| SERPINF1 | 0.670195451 | 1.44E-24 |
| SERPINH1 | 0.547434146 | 2.63E-15 |
| SERTAD2 | 0.60801909 | 2.25E-19 |
| SFMBT2 | 0.526937236 | 4.17E-14 |
| SFRP2 | 0.657163872 | 2.24E-23 |
| SFRP4 | 0.57389515 | 5.57E-17 |
| SGCD | 0.592410698 | 3.03E-18 |
| SGIP1 | 0.592105383 | 3.19E-18 |
| SGK269 | 0.641904026 | 4.68E-22 |
| SH2B3 | 0.594127528 | 2.29E-18 |
| SH2D1A | 0.563388419 | 2.68E-16 |
| SH3BP5 | 0.607652333 | 2.39E-19 |
| SH3GLB1 | 0.531158092 | 2.40E-14 |
| SH3PXD2A | 0.570811179 | 8.89E-17 |
| SH3PXD2B | 0.64226412 | 4.36E-22 |
| SHROOM4 | 0.594223409 | 2.26E-18 |
| SIGLEC10 | 0.51973135 | 1.05E-13 |
| SIGLEC1 | 0.505870593 | 5.90E-13 |
| SIGLEC5 | 0.60601685 | 3.16E-19 |
| SIGLEC7 | 0.542154279 | 5.46E-15 |
| SIGLEC9 | 0.541986538 | 5.59E-15 |
| SIGLECP3 | 0.523658774 | 6.37E-14 |
| SIRPA | 0.564469342 | 2.29E-16 |
| SIRPB1 | 0.576264916 | 3.88E-17 |
| SIRPB2 | 0.617902158 | 4.01E-20 |
| SIRPG | 0.546073741 | 3.18E-15 |
| SKIL | 0.512780344 | 2.52E-13 |
| SKI | 0.538745651 | 8.69E-15 |
| SLAMF1 | 0.549351585 | 2.01E-15 |
| SLAMF6 | 0.510792466 | 3.23E-13 |
| SLAMF7 | 0.551348564 | 1.52E-15 |
| SLAMF8 | 0.65646305 | 2.58E-23 |
| SLA | 0.613837189 | 8.20E-20 |
| SLC12A6 | 0.561236303 | 3.68E-16 |
| SLC15A3 | 0.512411067 | 2.64E-13 |
| SLC16A6 | 0.536582122 | 1.16E-14 |
| SLC1A3 | 0.535726502 | 1.30E-14 |
| SLC2A14 | 0.686327711 | 3.98E-26 |
| SLC2A3 | 0.722584957 | 4.98E-30 |
| SLC2A9 | 0.538301934 | 9.22E-15 |
| SLC30A7 | 0.500572068 | 1.12E-12 |
| SLC31A2 | 0.503546782 | 7.82E-13 |
| SLC36A1 | 0.592405182 | 3.04E-18 |
| SLC37A2 | 0.533744952 | 1.70E-14 |
| SLC4A7 | 0.565362497 | 2.01E-16 |
| SLC6A6 | 0.634888649 | 1.79E-21 |
| SLC9A9 | 0.522379126 | 7.51E-14 |
| SLCO2B1 | 0.551808814 | 1.43E-15 |
| SLFN11 | 0.692057327 | 1.05E-26 |
| SLFN12L | 0.512441823 | 2.63E-13 |
| SLFN12 | 0.610508636 | 1.46E-19 |
| SLFN5 | 0.520149951 | 9.99E-14 |
| SLIT2 | 0.615337732 | 6.31E-20 |
| SMO | 0.57260795 | 6.78E-17 |
| SNAI1 | 0.550751817 | 1.65E-15 |
| SNAI2 | 0.568962581 | 1.17E-16 |
| SNED1 | 0.500135846 | 1.18E-12 |
| SNTB2 | 0.71044708 | 1.18E-28 |
| SNX10 | 0.511357536 | 3.01E-13 |
| SNX20 | 0.620910556 | 2.34E-20 |
| SNX9 | 0.558854771 | 5.20E-16 |
| SOCS3 | 0.605743749 | 3.31E-19 |
| SOCS5 | 0.590940602 | 3.85E-18 |
| SORCS2 | 0.513497902 | 2.31E-13 |
| SP100 | 0.512137936 | 2.73E-13 |
| SP140 | 0.573067856 | 6.32E-17 |
| SPARC | 0.702298433 | 8.98E-28 |
| SPI1 | 0.512038685 | 2.77E-13 |
| SPN | 0.579174796 | 2.48E-17 |
| SPOCK1 | 0.616574141 | 5.07E-20 |
| SPON1 | 0.659029082 | 1.52E-23 |
| SPRED1 | 0.577123776 | 3.40E-17 |
| SPRY1 | 0.524085303 | 6.03E-14 |
| SPSB1 | 0.617832432 | 4.06E-20 |
| SRGAP2 | 0.501282274 | 1.03E-12 |
| SRGN | 0.613609622 | 8.54E-20 |
| SRPX2 | 0.655755181 | 2.98E-23 |
| SSC5D | 0.584846638 | 1.02E-17 |
| SSH1 | 0.679312082 | 1.95E-25 |
| SSPN | 0.675403442 | 4.64E-25 |
| ST6GAL2 | 0.706501453 | 3.18E-28 |
| ST8SIA4 | 0.620591309 | 2.48E-20 |
| STAG1 | 0.510087055 | 3.52E-13 |
| STARD8 | 0.542986946 | 4.87E-15 |
| STAT2 | 0.626940887 | 7.85E-21 |
| STK17B | 0.70063561 | 1.35E-27 |
| STK4 | 0.541410658 | 6.05E-15 |
| STON1 | 0.722353614 | 5.29E-30 |
| STX11 | 0.663885639 | 5.54E-24 |
| STX7 | 0.504465479 | 7.00E-13 |
| SULF1 | 0.758141069 | 1.64E-34 |
| SULF2 | 0.520166806 | 9.96E-14 |
| SVEP1 | 0.640951748 | 5.63E-22 |
| SWAP70 | 0.631379174 | 3.46E-21 |
| SYDE1 | 0.627167098 | 7.53E-21 |
| TAGAP | 0.679950833 | 1.69E-25 |
| TAGLN | 0.563273584 | 2.73E-16 |
| TBC1D2B | 0.513150975 | 2.41E-13 |
| TCF12 | 0.58107954 | 1.84E-17 |
| TCF21 | 0.565433884 | 1.99E-16 |
| TCF4 | 0.740226943 | 3.67E-32 |
| TDO2 | 0.636159687 | 1.41E-21 |
| TFEC | 0.672592968 | 8.59E-25 |
| TGFB3 | 0.595391955 | 1.87E-18 |
| TGFBR2 | 0.527042582 | 4.11E-14 |
| THBS1 | 0.71742451 | 1.95E-29 |
| THBS2 | 0.690581454 | 1.49E-26 |
| THY1 | 0.684689103 | 5.80E-26 |
| TIGIT | 0.635977318 | 1.46E-21 |
| TIMP2 | 0.621901268 | 1.96E-20 |
| TIMP3 | 0.639915765 | 6.87E-22 |
| TLR1 | 0.600267524 | 8.33E-19 |
| TLR2 | 0.51018631 | 3.48E-13 |
| TLR4 | 0.596337676 | 1.60E-18 |
| TLR6 | 0.631148505 | 3.61E-21 |
| TLR7 | 0.504763573 | 6.75E-13 |
| TLR8 | 0.546400272 | 3.04E-15 |
| TMEFF1 | 0.518953929 | 1.16E-13 |
| TMEM106A | 0.52952496 | 2.97E-14 |
| TMEM119 | 0.610581991 | 1.45E-19 |
| TMEM133 | 0.526210322 | 4.58E-14 |
| TMEM200A | 0.636042542 | 1.44E-21 |
| TMEM200B | 0.520433814 | 9.63E-14 |
| TMEM200C | 0.512741839 | 2.54E-13 |
| TMEM26 | 0.659439436 | 1.40E-23 |
| TMEM43 | 0.520488357 | 9.56E-14 |
| TMEM45A | 0.506500936 | 5.47E-13 |
| TMEM47 | 0.515854344 | 1.72E-13 |
| TMTC3 | 0.521781321 | 8.11E-14 |
| TNFAIP3 | 0.720727757 | 8.16E-30 |
| TNFAIP6 | 0.730436477 | 5.87E-31 |
| TNFRSF9 | 0.649431015 | 1.07E-22 |
| TNFSF13B | 0.58748572 | 6.70E-18 |
| TNFSF4 | 0.662127801 | 8.00E-24 |
| TNFSF8 | 0.611170642 | 1.31E-19 |
| TNIP3 | 0.576405161 | 3.80E-17 |
| TNS1 | 0.528832253 | 3.25E-14 |
| TPM4 | 0.571715591 | 7.76E-17 |
| TPP1 | 0.507224716 | 5.00E-13 |
| TRAF3IP3 | 0.502375521 | 9.00E-13 |
| TRAM2 | 0.511952101 | 2.80E-13 |
| TRANK1 | 0.594457797 | 2.17E-18 |
| TRAT1 | 0.513868264 | 2.20E-13 |
| TREM1 | 0.553217165 | 1.17E-15 |
| TRIB2 | 0.500468464 | 1.13E-12 |
| TRIL | 0.564678771 | 2.22E-16 |
| TRIM22 | 0.690189017 | 1.63E-26 |
| TRIM6 | 0.522999891 | 6.93E-14 |
| TRPC3 | 0.508166934 | 4.46E-13 |
| TRPS1 | 0.602544294 | 5.69E-19 |
| TRPV2 | 0.519989246 | 1.02E-13 |
| TSC22D2 | 0.546276144 | 3.09E-15 |
| TSHZ2 | 0.514211938 | 2.11E-13 |
| TSHZ3 | 0.58126028 | 1.79E-17 |
| TSPAN18 | 0.612128462 | 1.11E-19 |
| TSPAN9 | 0.523392717 | 6.59E-14 |
| TWIST1 | 0.671127039 | 1.18E-24 |
| TXLNB | 0.673159832 | 7.59E-25 |
| UACA | 0.571598975 | 7.90E-17 |
| UBASH3B | 0.508158847 | 4.46E-13 |
| UBE2E2 | 0.565043972 | 2.10E-16 |
| UBTD2 | 0.507484061 | 4.85E-13 |
| UGCG | 0.558238088 | 5.69E-16 |
| UHRF2 | 0.506777264 | 5.29E-13 |
| UNC5B | 0.520573834 | 9.46E-14 |
| UNC5C | 0.719780369 | 1.05E-29 |
| UVRAG | 0.549240194 | 2.05E-15 |
| VAMP7 | 0.548702268 | 2.21E-15 |
| VASH2 | 0.646447706 | 1.93E-22 |
| VCAM1 | 0.606282419 | 3.02E-19 |
| VCAN | 0.702601635 | 8.34E-28 |
| VCL | 0.556709756 | 7.09E-16 |
| VDR | 0.574242295 | 5.29E-17 |
| VEGFC | 0.517278839 | 1.44E-13 |
| VGLL3 | 0.728214897 | 1.08E-30 |
| VIM | 0.562143235 | 3.22E-16 |
| WAS | 0.534670123 | 1.50E-14 |
| WDFY4 | 0.601650145 | 6.61E-19 |
| WIPF1 | 0.79669239 | 2.44E-40 |
| WISP1 | 0.780951636 | 8.12E-38 |
| WIT1 | 0.566281605 | 1.75E-16 |
| WNT2 | 0.602966245 | 5.30E-19 |
| WNT5A | 0.629519208 | 4.88E-21 |
| WT1 | 0.648719849 | 1.23E-22 |
| WWC2 | 0.540001777 | 7.33E-15 |
| WWC3 | 0.604497644 | 4.09E-19 |
| WWTR1 | 0.53903416 | 8.35E-15 |
| XAF1 | 0.519557893 | 1.08E-13 |
| XIRP1 | 0.56702295 | 1.57E-16 |
| XRN1 | 0.50068018 | 1.10E-12 |
| YAP1 | 0.526359352 | 4.49E-14 |
| ZBTB38 | 0.560691926 | 3.98E-16 |
| ZCCHC24 | 0.572766744 | 6.62E-17 |
| ZCCHC5 | 0.542546298 | 5.18E-15 |
| ZEB1 | 0.66266393 | 7.15E-24 |
| ZEB2 | 0.71350858 | 5.38E-29 |
| ZFHX4 | 0.725506605 | 2.27E-30 |
| ZFP36L1 | 0.629586345 | 4.82E-21 |
| ZFPM2 | 0.752544517 | 9.35E-34 |
| ZNF217 | 0.505655887 | 6.06E-13 |
| ZNF267 | 0.547152376 | 2.74E-15 |
| ZNF281 | 0.567495311 | 1.46E-16 |
| ZNF423 | 0.569041531 | 1.16E-16 |
| ZNF469 | 0.578924491 | 2.57E-17 |
| ZNF518B | 0.571553661 | 7.95E-17 |
| ZNF521 | 0.735718222 | 1.34E-31 |
| ZNF532 | 0.712639205 | 6.72E-29 |
| ZNF804A | 0.505762022 | 5.98E-13 |
