# Supplementary material for: A Potential Prognostic Marker PRDM1 in Pancreatic Adenocarcinoma
Source: J Oncol. 2022 May 13;2022:1934381. doi: 10.1155/2022/1934381 (PMC9123419; doi:10.1155/2022/1934381)
Supplement: Supplementary 2 — Table S2: negative gene associated with PRDM1 in LinkedOmics database. [file 1934381.f2.docx]

**Table S2.** Negative gene associated with PRDM1 in LinkedOmics database

| Gene | Pearson-correlation | P-value |
| --- | --- | --- |
| ACOT8 | -0.525117176 | 5.28E-14 |
| AES | -0.540584333 | 6.77E-15 |
| AGFG2 | -0.504465357 | 7.00E-13 |
| ALG5 | -0.532466034 | 2.01E-14 |
| ALKBH4 | -0.544187699 | 4.13E-15 |
| ALKBH7 | -0.530175763 | 2.73E-14 |
| ARFIP2 | -0.54491024 | 3.74E-15 |
| ATG4D | -0.541780563 | 5.75E-15 |
| ATP5G2 | -0.523659261 | 6.37E-14 |
| C17orf28 | -0.503415868 | 7.94E-13 |
| C19orf70 | -0.503672707 | 7.70E-13 |
| C19orf73 | -0.552912974 | 1.22E-15 |
| C1orf56 | -0.552698488 | 1.26E-15 |
| C1orf66 | -0.511234875 | 3.06E-13 |
| C6orf130 | -0.531815387 | 2.20E-14 |
| C6orf136 | -0.5052448 | 6.37E-13 |
| C7orf55 | -0.517595865 | 1.38E-13 |
| C9orf163 | -0.544882621 | 3.75E-15 |
| C9orf7 | -0.568479816 | 1.26E-16 |
| C9orf86 | -0.567795228 | 1.40E-16 |
| CARKD | -0.530234175 | 2.71E-14 |
| CCDC56 | -0.537045006 | 1.09E-14 |
| CDC34 | -0.53187596 | 2.18E-14 |
| CHCHD2 | -0.514569624 | 2.02E-13 |
| CHCHD5 | -0.507155024 | 5.05E-13 |
| CISD3 | -0.530601464 | 2.58E-14 |
| COMTD1 | -0.521195967 | 8.74E-14 |
| COQ4 | -0.589581949 | 4.79E-18 |
| CYHR1 | -0.551601439 | 1.47E-15 |
| DCI | -0.59773323 | 1.27E-18 |
| DDRGK1 | -0.533917617 | 1.66E-14 |
| DHPS | -0.514547129 | 2.03E-13 |
| DHRS13 | -0.543459296 | 4.57E-15 |
| DNAJB2 | -0.505221963 | 6.39E-13 |
| DPM3 | -0.522169833 | 7.71E-14 |
| ECSIT | -0.536972242 | 1.10E-14 |
| ENDOG | -0.553695492 | 1.09E-15 |
| FAM195A | -0.541056893 | 6.35E-15 |
| FIS1 | -0.534380732 | 1.56E-14 |
| FIZ1 | -0.540350654 | 6.99E-15 |
| FN3K | -0.524029983 | 6.07E-14 |
| FOXA2 | -0.523513413 | 6.49E-14 |
| FOXP4 | -0.512465976 | 2.62E-13 |
| GET4 | -0.507781032 | 4.68E-13 |
| GFER | -0.560899885 | 3.86E-16 |
| GPAA1 | -0.525377935 | 5.10E-14 |
| HAGH | -0.507219231 | 5.01E-13 |
| HDAC11 | -0.51549088 | 1.80E-13 |
| ICA1 | -0.572797147 | 6.59E-17 |
| ILVBL | -0.566314317 | 1.74E-16 |
| INPP5J | -0.534785125 | 1.48E-14 |
| IRF2BP1 | -0.531002777 | 2.45E-14 |
| JAGN1 | -0.537311159 | 1.05E-14 |
| JTB | -0.524976211 | 5.37E-14 |
| KIAA0114 | -0.505433898 | 6.22E-13 |
| KIAA1543 | -0.562693364 | 2.97E-16 |
| KIF9 | -0.54632297 | 3.07E-15 |
| KLHDC9 | -0.529507697 | 2.98E-14 |
| LCMT1 | -0.532127509 | 2.11E-14 |
| LIAS | -0.511360674 | 3.01E-13 |
| LOC100128822 | -0.523303896 | 6.67E-14 |
| LOC113230 | -0.509744388 | 3.67E-13 |
| LSM4 | -0.508677162 | 4.19E-13 |
| MAGIX | -0.538700647 | 8.74E-15 |
| MANBAL | -0.568411697 | 1.28E-16 |
| MANEAL | -0.501963888 | 9.46E-13 |
| MCAT | -0.520695044 | 9.32E-14 |
| MDH2 | -0.511661175 | 2.90E-13 |
| MFSD3 | -0.576619609 | 3.67E-17 |
| MGC70857 | -0.511158388 | 3.09E-13 |
| MLST8 | -0.538473885 | 9.01E-15 |
| MMAB | -0.556878617 | 6.92E-16 |
| MNX1 | -0.507356814 | 4.92E-13 |
| MRP63 | -0.559437758 | 4.78E-16 |
| MRPL38 | -0.507630723 | 4.76E-13 |
| MRPS26 | -0.550596964 | 1.69E-15 |
| MRPS34 | -0.501046183 | 1.06E-12 |
| NDUFA7 | -0.557379195 | 6.44E-16 |
| NDUFB10 | -0.515052981 | 1.90E-13 |
| NDUFB11 | -0.525454699 | 5.05E-14 |
| NDUFB7 | -0.511023984 | 3.14E-13 |
| NDUFC1 | -0.541687002 | 5.82E-15 |
| NDUFS6 | -0.523301452 | 6.67E-14 |
| NECAB3 | -0.507752821 | 4.69E-13 |
| NHP2 | -0.539064111 | 8.32E-15 |
| NPRL2 | -0.524482739 | 5.73E-14 |
| NR2F6 | -0.533462099 | 1.77E-14 |
| NTHL1 | -0.525373415 | 5.11E-14 |
| NUDT16L1 | -0.609876822 | 1.63E-19 |
| P4HTM | -0.524656685 | 5.60E-14 |
| PCBD1 | -0.591225946 | 3.68E-18 |
| PDRG1 | -0.512392473 | 2.65E-13 |
| PICK1 | -0.533726847 | 1.70E-14 |
| PMVK | -0.539267033 | 8.09E-15 |
| POLD2 | -0.540095262 | 7.23E-15 |
| POLR2I | -0.517398855 | 1.42E-13 |
| POP7 | -0.515339724 | 1.83E-13 |
| PPP1R16A | -0.540708673 | 6.65E-15 |
| PTGES2 | -0.546090082 | 3.17E-15 |
| PTOV1 | -0.510055033 | 3.54E-13 |
| PXMP2 | -0.513487129 | 2.31E-13 |
| PYCRL | -0.508839736 | 4.11E-13 |
| RAB17 | -0.509681455 | 3.70E-13 |
| RAB3D | -0.524088313 | 6.03E-14 |
| ROMO1 | -0.523581205 | 6.43E-14 |
| SDHAF1 | -0.547674045 | 2.55E-15 |
| SERGEF | -0.557144005 | 6.66E-16 |
| SNHG11 | -0.507665325 | 4.74E-13 |
| SNHG8 | -0.583056786 | 1.35E-17 |
| SPR | -0.50618805 | 5.68E-13 |
| STARD10 | -0.529296593 | 3.06E-14 |
| STK16 | -0.519934404 | 1.03E-13 |
| STUB1 | -0.508545882 | 4.26E-13 |
| SURF1 | -0.522854045 | 7.07E-14 |
| SURF2 | -0.561453096 | 3.57E-16 |
| SURF6 | -0.528004173 | 3.63E-14 |
| TIGD5 | -0.53812916 | 9.44E-15 |
| TIMM13 | -0.559388258 | 4.81E-16 |
| TIMM16 | -0.50259818 | 8.77E-13 |
| TMEM186 | -0.527663311 | 3.79E-14 |
| TMEM205 | -0.567303945 | 1.50E-16 |
| TOLLIP | -0.514225312 | 2.11E-13 |
| TP53TG1 | -0.507790402 | 4.67E-13 |
| TPRN | -0.528435735 | 3.43E-14 |
| TRAP1 | -0.547289681 | 2.69E-15 |
| TSEN54 | -0.504285344 | 7.15E-13 |
| TSTD1 | -0.599579872 | 9.34E-19 |
| TUSC2 | -0.500756052 | 1.09E-12 |
| UFSP1 | -0.504596649 | 6.89E-13 |
| WBSCR22 | -0.533892094 | 1.67E-14 |
| WDR13 | -0.500534635 | 1.12E-12 |
| WDR25 | -0.553296243 | 1.15E-15 |
| ZFAND2B | -0.541613942 | 5.88E-15 |
| ZNF511 | -0.51017781 | 3.48E-13 |
| ZNF696 | -0.522043481 | 7.84E-14 |
