# Supplementary material for: A Potential Prognostic Marker PRDM1 in Pancreatic Adenocarcinoma
Source: J Oncol. 2022 May 13;2022:1934381. doi: 10.1155/2022/1934381 (PMC9123419; doi:10.1155/2022/1934381)
Supplement: Supplementary 3 — Table S3: the coexpressed genes with positive correlation between GO enrichment pathway and PRDM1 via DAVID database. [file 1934381.f3.docx]

**Table S3.** The co-expressed genes with positive correlation between GO enrichment pathway and PRDM1 via David database.

| Category | Term | Count | Ratio | P-value | FDR |
| --- | --- | --- | --- | --- | --- |
| GOTERM_BP_DIRECT | GO:0030198~extracellular matrix organization | 70 | 6.469500924 | 1.01E-35 | 3.61E-32 |
| GOTERM_BP_DIRECT | GO:0007155~cell adhesion | 106 | 9.796672828 | 7.95E-35 | 1.43E-31 |
| GOTERM_BP_DIRECT | GO:0006954~inflammatory response | 78 | 7.208872458 | 2.60E-22 | 3.11E-19 |
| GOTERM_BP_DIRECT | GO:0006955~immune response | 81 | 7.486136784 | 3.12E-21 | 2.79E-18 |
| GOTERM_BP_DIRECT | GO:0030574~collagen catabolic process | 27 | 2.495378928 | 4.88E-16 | 3.50E-13 |
| GOTERM_BP_DIRECT | GO:0007165~signal transduction | 138 | 12.75415896 | 1.32E-15 | 7.87E-13 |
| GOTERM_BP_DIRECT | GO:0050900~leukocyte migration | 35 | 3.234750462 | 1.07E-14 | 5.48E-12 |
| GOTERM_BP_DIRECT | GO:0030199~collagen fibril organization | 19 | 1.756007394 | 1.20E-12 | 5.38E-10 |
| GOTERM_BP_DIRECT | GO:0050776~regulation of immune response | 39 | 3.604436229 | 2.84E-12 | 1.13E-09 |
| GOTERM_BP_DIRECT | GO:0030335~positive regulation of cell migration | 37 | 3.419593346 | 1.47E-10 | 5.28E-08 |
| GOTERM_BP_DIRECT | GO:0007160~cell-matrix adhesion | 25 | 2.310536044 | 2.42E-10 | 7.89E-08 |
| GOTERM_BP_DIRECT | GO:0007229~integrin-mediated signaling pathway | 26 | 2.402957486 | 3.65E-10 | 1.09E-07 |
| GOTERM_BP_DIRECT | GO:0035987~endodermal cell differentiation | 14 | 1.293900185 | 8.79E-10 | 2.42E-07 |
| GOTERM_BP_DIRECT | GO:0045087~innate immune response | 60 | 5.545286506 | 1.04E-09 | 2.66E-07 |
| GOTERM_BP_DIRECT | GO:0022617~extracellular matrix disassembly | 22 | 2.033271719 | 1.57E-09 | 3.76E-07 |
| GOTERM_BP_DIRECT | GO:0001525~angiogenesis | 39 | 3.604436229 | 2.87E-09 | 6.42E-07 |
| GOTERM_BP_DIRECT | GO:0002250~adaptive immune response | 30 | 2.772643253 | 8.67E-09 | 1.83E-06 |
| GOTERM_BP_DIRECT | GO:0043547~positive regulation of GTPase activity | 69 | 6.377079482 | 1.42E-08 | 2.82E-06 |
| GOTERM_BP_DIRECT | GO:0006968~cellular defense response | 18 | 1.663585952 | 6.42E-08 | 1.21E-05 |
| GOTERM_BP_DIRECT | GO:0001649~osteoblast differentiation | 23 | 2.125693161 | 1.31E-07 | 2.34E-05 |
| GOTERM_BP_DIRECT | GO:0006935~chemotaxis | 25 | 2.310536044 | 1.52E-07 | 2.55E-05 |
| GOTERM_BP_DIRECT | GO:0008360~regulation of cell shape | 27 | 2.495378928 | 1.56E-07 | 2.55E-05 |
| GOTERM_BP_DIRECT | GO:0030593~neutrophil chemotaxis | 18 | 1.663585952 | 1.75E-07 | 2.72E-05 |
| GOTERM_BP_DIRECT | GO:0042102~positive regulation of T cell proliferation | 17 | 1.57116451 | 2.39E-07 | 3.53E-05 |
| GOTERM_BP_DIRECT | GO:0007204~positive regulation of cytosolic calcium ion concentration | 26 | 2.402957486 | 2.46E-07 | 3.53E-05 |
| GOTERM_BP_DIRECT | GO:0001501~skeletal system development | 26 | 2.402957486 | 3.82E-07 | 5.27E-05 |
| GOTERM_BP_DIRECT | GO:0031295~T cell costimulation | 19 | 1.756007394 | 4.59E-07 | 6.09E-05 |
| GOTERM_BP_DIRECT | GO:0007169~transmembrane receptor protein tyrosine kinase signaling pathway | 21 | 1.940850277 | 6.26E-07 | 8.01E-05 |
| GOTERM_BP_DIRECT | GO:0010811~positive regulation of cell-substrate adhesion | 13 | 1.201478743 | 1.06E-06 | 1.31E-04 |
| GOTERM_BP_DIRECT | GO:0060021~palate development | 18 | 1.663585952 | 1.52E-06 | 1.82E-04 |
| GOTERM_BP_DIRECT | GO:0042110~T cell activation | 14 | 1.293900185 | 2.05E-06 | 2.37E-04 |
| GOTERM_BP_DIRECT | GO:0032331~negative regulation of chondrocyte differentiation | 9 | 0.831792976 | 2.12E-06 | 2.38E-04 |
| GOTERM_BP_DIRECT | GO:0071222~cellular response to lipopolysaccharide | 22 | 2.033271719 | 2.37E-06 | 2.57E-04 |
| GOTERM_BP_DIRECT | GO:0007507~heart development | 29 | 2.680221811 | 3.16E-06 | 3.16E-04 |
| GOTERM_BP_DIRECT | GO:0032733~positive regulation of interleukin-10 production | 10 | 0.924214418 | 3.17E-06 | 3.16E-04 |
| GOTERM_BP_DIRECT | GO:0045766~positive regulation of angiogenesis | 22 | 2.033271719 | 3.18E-06 | 3.16E-04 |
| GOTERM_BP_DIRECT | GO:0070374~positive regulation of ERK1 and ERK2 cascade | 28 | 2.58780037 | 4.01E-06 | 3.89E-04 |
| GOTERM_BP_DIRECT | GO:0007267~cell-cell signaling | 35 | 3.234750462 | 6.33E-06 | 5.97E-04 |
| GOTERM_BP_DIRECT | GO:0001816~cytokine production | 10 | 0.924214418 | 7.12E-06 | 6.38E-04 |
| GOTERM_BP_DIRECT | GO:0007159~leukocyte cell-cell adhesion | 10 | 0.924214418 | 7.12E-06 | 6.38E-04 |
| GOTERM_BP_DIRECT | GO:0030336~negative regulation of cell migration | 19 | 1.756007394 | 9.28E-06 | 8.01E-04 |
| GOTERM_BP_DIRECT | GO:0006801~superoxide metabolic process | 9 | 0.831792976 | 9.38E-06 | 8.01E-04 |
| GOTERM_BP_DIRECT | GO:0002063~chondrocyte development | 8 | 0.739371534 | 1.02E-05 | 8.50E-04 |
| GOTERM_BP_DIRECT | GO:0000902~cell morphogenesis | 15 | 1.386321627 | 1.12E-05 | 9.16E-04 |
| GOTERM_BP_DIRECT | GO:0050852~T cell receptor signaling pathway | 24 | 2.218114603 | 1.81E-05 | 0.001441644 |
| GOTERM_BP_DIRECT | GO:0014068~positive regulation of phosphatidylinositol 3-kinase signaling | 15 | 1.386321627 | 2.00E-05 | 0.001525449 |
| GOTERM_BP_DIRECT | GO:0060326~cell chemotaxis | 15 | 1.386321627 | 2.00E-05 | 0.001525449 |
| GOTERM_BP_DIRECT | GO:0034113~heterotypic cell-cell adhesion | 9 | 0.831792976 | 2.14E-05 | 0.001600541 |
| GOTERM_BP_DIRECT | GO:0045893~positive regulation of transcription, DNA-templated | 55 | 5.083179298 | 2.73E-05 | 0.001990713 |
| GOTERM_BP_DIRECT | GO:0002407~dendritic cell chemotaxis | 8 | 0.739371534 | 2.78E-05 | 0.001990713 |
| GOTERM_BP_DIRECT | GO:0010628~positive regulation of gene expression | 34 | 3.14232902 | 3.07E-05 | 0.002119762 |
| GOTERM_BP_DIRECT | GO:0030324~lung development | 16 | 1.478743068 | 3.07E-05 | 0.002119762 |
| GOTERM_BP_DIRECT | GO:0010977~negative regulation of neuron projection development | 12 | 1.109057301 | 3.56E-05 | 0.002405221 |
| GOTERM_BP_DIRECT | GO:0051056~regulation of small GTPase mediated signal transduction | 22 | 2.033271719 | 3.63E-05 | 0.002413212 |
| GOTERM_BP_DIRECT | GO:0001568~blood vessel development | 11 | 1.01663586 | 4.99E-05 | 0.003196523 |
| GOTERM_BP_DIRECT | GO:0034446~substrate adhesion-dependent cell spreading | 11 | 1.01663586 | 4.99E-05 | 0.003196523 |
| GOTERM_BP_DIRECT | GO:0060333~interferon-gamma-mediated signaling pathway | 15 | 1.386321627 | 5.67E-05 | 0.003560416 |
| GOTERM_BP_DIRECT | GO:0001503~ossification | 16 | 1.478743068 | 5.76E-05 | 0.003560416 |
| GOTERM_BP_DIRECT | GO:0030890~positive regulation of B cell proliferation | 11 | 1.01663586 | 6.36E-05 | 0.003866636 |
| GOTERM_BP_DIRECT | GO:0007166~cell surface receptor signaling pathway | 34 | 3.14232902 | 7.51E-05 | 0.004488318 |
| GOTERM_BP_DIRECT | GO:0045944~positive regulation of transcription from RNA polymerase II promoter | 88 | 8.133086876 | 7.71E-05 | 0.004532723 |
| GOTERM_BP_DIRECT | GO:0032743~positive regulation of interleukin-2 production | 7 | 0.646950092 | 8.20E-05 | 0.004739902 |
| GOTERM_BP_DIRECT | GO:0035025~positive regulation of Rho protein signal transduction | 9 | 0.831792976 | 8.48E-05 | 0.004823978 |
| GOTERM_BP_DIRECT | GO:0032496~response to lipopolysaccharide | 24 | 2.218114603 | 9.40E-05 | 0.00526686 |
| GOTERM_BP_DIRECT | GO:0071560~cellular response to transforming growth factor beta stimulus | 12 | 1.109057301 | 1.03E-04 | 0.005684165 |
| GOTERM_BP_DIRECT | GO:0001837~epithelial to mesenchymal transition | 10 | 0.924214418 | 1.13E-04 | 0.006115114 |
| GOTERM_BP_DIRECT | GO:0035329~hippo signaling | 9 | 0.831792976 | 1.14E-04 | 0.006115114 |
| GOTERM_BP_DIRECT | GO:0033627~cell adhesion mediated by integrin | 7 | 0.646950092 | 1.30E-04 | 0.006769842 |
| GOTERM_BP_DIRECT | GO:0030168~platelet activation | 19 | 1.756007394 | 1.30E-04 | 0.006769842 |
| GOTERM_BP_DIRECT | GO:0048048~embryonic eye morphogenesis | 6 | 0.554528651 | 1.38E-04 | 0.006958762 |
| GOTERM_BP_DIRECT | GO:0032060~bleb assembly | 6 | 0.554528651 | 1.38E-04 | 0.006958762 |
| GOTERM_BP_DIRECT | GO:0032689~negative regulation of interferon-gamma production | 9 | 0.831792976 | 1.52E-04 | 0.007454624 |
| GOTERM_BP_DIRECT | GO:0032715~negative regulation of interleukin-6 production | 9 | 0.831792976 | 1.52E-04 | 0.007454624 |
| GOTERM_BP_DIRECT | GO:0032964~collagen biosynthetic process | 5 | 0.462107209 | 1.63E-04 | 0.007877678 |
| GOTERM_BP_DIRECT | GO:0000187~activation of MAPK activity | 18 | 1.663585952 | 1.65E-04 | 0.007877678 |
| GOTERM_BP_DIRECT | GO:0071345~cellular response to cytokine stimulus | 8 | 0.739371534 | 1.88E-04 | 0.008855533 |
| GOTERM_BP_DIRECT | GO:0043065~positive regulation of apoptotic process | 35 | 3.234750462 | 1.92E-04 | 0.008918908 |
| GOTERM_BP_DIRECT | GO:0090026~positive regulation of monocyte chemotaxis | 7 | 0.646950092 | 1.97E-04 | 0.009072562 |
| GOTERM_BP_DIRECT | GO:0042060~wound healing | 15 | 1.386321627 | 2.17E-04 | 0.009847575 |
| GOTERM_BP_DIRECT | GO:0070098~chemokine-mediated signaling pathway | 14 | 1.293900185 | 2.27E-04 | 0.010147266 |
| GOTERM_BP_DIRECT | GO:0008015~blood circulation | 11 | 1.01663586 | 2.31E-04 | 0.010147266 |
| GOTERM_BP_DIRECT | GO:0090090~negative regulation of canonical Wnt signaling pathway | 23 | 2.125693161 | 2.32E-04 | 0.010147266 |
| GOTERM_BP_DIRECT | GO:0045060~negative thymic T cell selection | 6 | 0.554528651 | 2.40E-04 | 0.010260395 |
| GOTERM_BP_DIRECT | GO:0042535~positive regulation of tumor necrosis factor biosynthetic process | 6 | 0.554528651 | 2.40E-04 | 0.010260395 |
| GOTERM_BP_DIRECT | GO:0048010~vascular endothelial growth factor receptor signaling pathway | 14 | 1.293900185 | 2.62E-04 | 0.011059296 |
| GOTERM_BP_DIRECT | GO:0032729~positive regulation of interferon-gamma production | 11 | 1.01663586 | 2.80E-04 | 0.011684582 |
| GOTERM_BP_DIRECT | GO:0032720~negative regulation of tumor necrosis factor production | 10 | 0.924214418 | 2.84E-04 | 0.011711074 |
| GOTERM_BP_DIRECT | GO:0050729~positive regulation of inflammatory response | 14 | 1.293900185 | 3.02E-04 | 0.012307462 |
| GOTERM_BP_DIRECT | GO:0008285~negative regulation of cell proliferation | 42 | 3.881700555 | 3.20E-04 | 0.01290651 |
| GOTERM_BP_DIRECT | GO:0002576~platelet degranulation | 17 | 1.57116451 | 3.31E-04 | 0.013169438 |
| GOTERM_BP_DIRECT | GO:0061298~retina vasculature development in camera-type eye | 5 | 0.462107209 | 3.63E-04 | 0.014285671 |
| GOTERM_BP_DIRECT | GO:0043206~extracellular fibril organization | 6 | 0.554528651 | 3.92E-04 | 0.014956972 |
| GOTERM_BP_DIRECT | GO:0036066~protein O-linked fucosylation | 6 | 0.554528651 | 3.92E-04 | 0.014956972 |
| GOTERM_BP_DIRECT | GO:0045086~positive regulation of interleukin-2 biosynthetic process | 6 | 0.554528651 | 3.92E-04 | 0.014956972 |
| GOTERM_BP_DIRECT | GO:0030206~chondroitin sulfate biosynthetic process | 8 | 0.739371534 | 4.53E-04 | 0.01709438 |
| GOTERM_BP_DIRECT | GO:0006928~movement of cell or subcellular component | 15 | 1.386321627 | 4.70E-04 | 0.017562253 |
| GOTERM_BP_DIRECT | GO:0006952~defense response | 13 | 1.201478743 | 4.83E-04 | 0.017863537 |
| GOTERM_BP_DIRECT | GO:0002755~MyD88-dependent toll-like receptor signaling pathway | 9 | 0.831792976 | 5.22E-04 | 0.018885018 |
| GOTERM_BP_DIRECT | GO:0010718~positive regulation of epithelial to mesenchymal transition | 9 | 0.831792976 | 5.22E-04 | 0.018885018 |
| GOTERM_BP_DIRECT | GO:0045669~positive regulation of osteoblast differentiation | 12 | 1.109057301 | 6.70E-04 | 0.024018818 |
| GOTERM_BP_DIRECT | GO:0001763~morphogenesis of a branching structure | 5 | 0.462107209 | 6.91E-04 | 0.024064994 |
| GOTERM_BP_DIRECT | GO:0050702~interleukin-1 beta secretion | 5 | 0.462107209 | 6.91E-04 | 0.024064994 |
| GOTERM_BP_DIRECT | GO:0034138~toll-like receptor 3 signaling pathway | 5 | 0.462107209 | 6.91E-04 | 0.024064994 |
| GOTERM_BP_DIRECT | GO:0046718~viral entry into host cell | 14 | 1.293900185 | 7.54E-04 | 0.02599323 |
| GOTERM_BP_DIRECT | GO:0043372~positive regulation of CD4-positive, alpha-beta T cell differentiation | 4 | 0.369685767 | 7.79E-04 | 0.026368599 |
| GOTERM_BP_DIRECT | GO:0032753~positive regulation of interleukin-4 production | 7 | 0.646950092 | 7.80E-04 | 0.026368599 |
| GOTERM_BP_DIRECT | GO:0048565~digestive tract development | 9 | 0.831792976 | 7.96E-04 | 0.02667485 |
| GOTERM_BP_DIRECT | GO:0007275~multicellular organism development | 50 | 4.621072089 | 8.23E-04 | 0.027310996 |
| GOTERM_BP_DIRECT | GO:0097190~apoptotic signaling pathway | 13 | 1.201478743 | 8.31E-04 | 0.027336209 |
| GOTERM_BP_DIRECT | GO:0035556~intracellular signal transduction | 41 | 3.789279113 | 8.73E-04 | 0.028465979 |
| GOTERM_BP_DIRECT | GO:0070208~protein heterotrimerization | 6 | 0.554528651 | 8.98E-04 | 0.028739793 |
| GOTERM_BP_DIRECT | GO:0045579~positive regulation of B cell differentiation | 6 | 0.554528651 | 8.98E-04 | 0.028739793 |
| GOTERM_BP_DIRECT | GO:0001569~patterning of blood vessels | 8 | 0.739371534 | 9.56E-04 | 0.030113813 |
| GOTERM_BP_DIRECT | GO:0050731~positive regulation of peptidyl-tyrosine phosphorylation | 14 | 1.293900185 | 9.58E-04 | 0.030113813 |
| GOTERM_BP_DIRECT | GO:0006915~apoptotic process | 53 | 4.898336414 | 0.001009637 | 0.03147433 |
| GOTERM_BP_DIRECT | GO:0030514~negative regulation of BMP signaling pathway | 10 | 0.924214418 | 0.001066536 | 0.032679769 |
| GOTERM_BP_DIRECT | GO:0032755~positive regulation of interleukin-6 production | 10 | 0.924214418 | 0.001066536 | 0.032679769 |
| GOTERM_BP_DIRECT | GO:0010629~negative regulation of gene expression | 19 | 1.756007394 | 0.001139961 | 0.034633552 |
| GOTERM_BP_DIRECT | GO:0046854~phosphatidylinositol phosphorylation | 15 | 1.386321627 | 0.001167174 | 0.034868303 |
| GOTERM_BP_DIRECT | GO:0046641~positive regulation of alpha-beta T cell proliferation | 5 | 0.462107209 | 0.001186592 | 0.034868303 |
| GOTERM_BP_DIRECT | GO:0046007~negative regulation of activated T cell proliferation | 5 | 0.462107209 | 0.001186592 | 0.034868303 |
| GOTERM_BP_DIRECT | GO:0032835~glomerulus development | 5 | 0.462107209 | 0.001186592 | 0.034868303 |
| GOTERM_BP_DIRECT | GO:0030334~regulation of cell migration | 13 | 1.201478743 | 0.001211576 | 0.03531302 |
| GOTERM_BP_DIRECT | GO:0008284~positive regulation of cell proliferation | 45 | 4.15896488 | 0.001345973 | 0.038898422 |
| GOTERM_BP_DIRECT | GO:0050718~positive regulation of interleukin-1 beta secretion | 7 | 0.646950092 | 0.001356291 | 0.038898422 |
| GOTERM_BP_DIRECT | GO:0045860~positive regulation of protein kinase activity | 10 | 0.924214418 | 0.001475744 | 0.041192253 |
| GOTERM_BP_DIRECT | GO:0032760~positive regulation of tumor necrosis factor production | 10 | 0.924214418 | 0.001475744 | 0.041192253 |
| GOTERM_BP_DIRECT | GO:0071230~cellular response to amino acid stimulus | 10 | 0.924214418 | 0.001475744 | 0.041192253 |
| GOTERM_BP_DIRECT | GO:0060325~face morphogenesis | 8 | 0.739371534 | 0.001482232 | 0.041192253 |
| GOTERM_BP_DIRECT | GO:0040037~negative regulation of fibroblast growth factor receptor signaling pathway | 5 | 0.462107209 | 0.001885776 | 0.051606923 |
| GOTERM_BP_DIRECT | GO:0002430~complement receptor mediated signaling pathway | 5 | 0.462107209 | 0.001885776 | 0.051606923 |
| GOTERM_BP_DIRECT | GO:0071356~cellular response to tumor necrosis factor | 16 | 1.478743068 | 0.001967918 | 0.053045014 |
| GOTERM_BP_DIRECT | GO:0009615~response to virus | 16 | 1.478743068 | 0.001967918 | 0.053045014 |
| GOTERM_BP_DIRECT | GO:0051092~positive regulation of NF-kappaB transcription factor activity | 18 | 1.663585952 | 0.002086362 | 0.055817978 |
| GOTERM_BP_DIRECT | GO:0050728~negative regulation of inflammatory response | 13 | 1.201478743 | 0.002159687 | 0.057351685 |
| GOTERM_BP_DIRECT | GO:0001974~blood vessel remodeling | 8 | 0.739371534 | 0.002211189 | 0.058287583 |
| GOTERM_BP_DIRECT | GO:0032722~positive regulation of chemokine production | 6 | 0.554528651 | 0.00239356 | 0.0626344 |
| GOTERM_BP_DIRECT | GO:1902042~negative regulation of extrinsic apoptotic signaling pathway via death domain receptors | 8 | 0.739371534 | 0.002666102 | 0.06926069 |
| GOTERM_BP_DIRECT | GO:0042327~positive regulation of phosphorylation | 7 | 0.646950092 | 0.002766914 | 0.069870033 |
| GOTERM_BP_DIRECT | GO:0071347~cellular response to interleukin-1 | 12 | 1.109057301 | 0.002776551 | 0.069870033 |
| GOTERM_BP_DIRECT | GO:0001666~response to hypoxia | 21 | 1.940850277 | 0.002799585 | 0.069870033 |
| GOTERM_BP_DIRECT | GO:0016477~cell migration | 21 | 1.940850277 | 0.002799585 | 0.069870033 |
| GOTERM_BP_DIRECT | GO:0007264~small GTPase mediated signal transduction | 27 | 2.495378928 | 0.002816997 | 0.069870033 |
| GOTERM_BP_DIRECT | GO:0010759~positive regulation of macrophage chemotaxis | 5 | 0.462107209 | 0.002825985 | 0.069870033 |
| GOTERM_BP_DIRECT | GO:0050707~regulation of cytokine secretion | 5 | 0.462107209 | 0.002825985 | 0.069870033 |
| GOTERM_BP_DIRECT | GO:0042127~regulation of cell proliferation | 22 | 2.033271719 | 0.002980619 | 0.073052835 |
| GOTERM_BP_DIRECT | GO:0045892~negative regulation of transcription, DNA-templated | 46 | 4.251386322 | 0.002995472 | 0.073052835 |
| GOTERM_BP_DIRECT | GO:0016485~protein processing | 12 | 1.109057301 | 0.003106843 | 0.074909691 |
| GOTERM_BP_DIRECT | GO:0010596~negative regulation of endothelial cell migration | 6 | 0.554528651 | 0.003155192 | 0.074909691 |
| GOTERM_BP_DIRECT | GO:0034142~toll-like receptor 4 signaling pathway | 6 | 0.554528651 | 0.003155192 | 0.074909691 |
| GOTERM_BP_DIRECT | GO:0035904~aorta development | 6 | 0.554528651 | 0.003155192 | 0.074909691 |
| GOTERM_BP_DIRECT | GO:0001932~regulation of protein phosphorylation | 8 | 0.739371534 | 0.003189528 | 0.075226687 |
| GOTERM_BP_DIRECT | GO:0002053~positive regulation of mesenchymal cell proliferation | 7 | 0.646950092 | 0.003420394 | 0.079624117 |
| GOTERM_BP_DIRECT | GO:0001958~endochondral ossification | 7 | 0.646950092 | 0.003420394 | 0.079624117 |
| GOTERM_BP_DIRECT | GO:0009611~response to wounding | 11 | 1.01663586 | 0.003554572 | 0.081311586 |
| GOTERM_BP_DIRECT | GO:0001957~intramembranous ossification | 4 | 0.369685767 | 0.003560926 | 0.081311586 |
| GOTERM_BP_DIRECT | GO:0097094~craniofacial suture morphogenesis | 4 | 0.369685767 | 0.003560926 | 0.081311586 |
| GOTERM_BP_DIRECT | GO:0050918~positive chemotaxis | 8 | 0.739371534 | 0.003787871 | 0.085946319 |
| GOTERM_BP_DIRECT | GO:0051607~defense response to virus | 20 | 1.848428835 | 0.00392407 | 0.088376552 |
| GOTERM_BP_DIRECT | GO:0050853~B cell receptor signaling pathway | 10 | 0.924214418 | 0.00398564 | 0.088376552 |
| GOTERM_BP_DIRECT | GO:0050777~negative regulation of immune response | 5 | 0.462107209 | 0.004042888 | 0.088376552 |
| GOTERM_BP_DIRECT | GO:0050710~negative regulation of cytokine secretion | 5 | 0.462107209 | 0.004042888 | 0.088376552 |
| GOTERM_BP_DIRECT | GO:0032695~negative regulation of interleukin-12 production | 5 | 0.462107209 | 0.004042888 | 0.088376552 |
| GOTERM_BP_DIRECT | GO:0048846~axon extension involved in axon guidance | 5 | 0.462107209 | 0.004042888 | 0.088376552 |
| GOTERM_BP_DIRECT | GO:0048260~positive regulation of receptor-mediated endocytosis | 6 | 0.554528651 | 0.004076989 | 0.088581844 |
| GOTERM_BP_DIRECT | GO:0000122~negative regulation of transcription from RNA polymerase II promoter | 61 | 5.637707948 | 0.004153424 | 0.089241503 |
| GOTERM_BP_DIRECT | GO:0010575~positive regulation of vascular endothelial growth factor production | 7 | 0.646950092 | 0.004182029 | 0.089241503 |
| GOTERM_BP_DIRECT | GO:0002224~toll-like receptor signaling pathway | 7 | 0.646950092 | 0.004182029 | 0.089241503 |
| GOTERM_BP_DIRECT | GO:0019221~cytokine-mediated signaling pathway | 17 | 1.57116451 | 0.004388973 | 0.093103369 |
| GOTERM_BP_DIRECT | GO:0001570~vasculogenesis | 10 | 0.924214418 | 0.005113027 | 0.107824708 |
| GOTERM_BP_DIRECT | GO:0014911~positive regulation of smooth muscle cell migration | 6 | 0.554528651 | 0.005176099 | 0.108516463 |
| GOTERM_BP_DIRECT | GO:0042130~negative regulation of T cell proliferation | 8 | 0.739371534 | 0.005235665 | 0.109127089 |
| GOTERM_BP_DIRECT | GO:0045730~respiratory burst | 5 | 0.462107209 | 0.005570182 | 0.115428343 |
| GOTERM_BP_DIRECT | GO:0071346~cellular response to interferon-gamma | 10 | 0.924214418 | 0.005762117 | 0.118160887 |
| GOTERM_BP_DIRECT | GO:0014066~regulation of phosphatidylinositol 3-kinase signaling | 12 | 1.109057301 | 0.005808407 | 0.118160887 |
| GOTERM_BP_DIRECT | GO:0045666~positive regulation of neuron differentiation | 12 | 1.109057301 | 0.005808407 | 0.118160887 |
| GOTERM_BP_DIRECT | GO:0007517~muscle organ development | 13 | 1.201478743 | 0.005853648 | 0.118160887 |
| GOTERM_BP_DIRECT | GO:0007411~axon guidance | 19 | 1.756007394 | 0.005866845 | 0.118160887 |
| GOTERM_BP_DIRECT | GO:0043305~negative regulation of mast cell degranulation | 4 | 0.369685767 | 0.005959654 | 0.11935956 |
| GOTERM_BP_DIRECT | GO:0042113~B cell activation | 7 | 0.646950092 | 0.006070355 | 0.120901234 |
| GOTERM_BP_DIRECT | GO:0007224~smoothened signaling pathway | 11 | 1.01663586 | 0.006887842 | 0.136424945 |
| GOTERM_BP_DIRECT | GO:0048706~embryonic skeletal system development | 7 | 0.646950092 | 0.007217386 | 0.140478864 |
| GOTERM_BP_DIRECT | GO:0030217~T cell differentiation | 7 | 0.646950092 | 0.007217386 | 0.140478864 |
| GOTERM_BP_DIRECT | GO:0030097~hemopoiesis | 10 | 0.924214418 | 0.007249258 | 0.140478864 |
| GOTERM_BP_DIRECT | GO:0051216~cartilage development | 10 | 0.924214418 | 0.007249258 | 0.140478864 |
| GOTERM_BP_DIRECT | GO:0042981~regulation of apoptotic process | 23 | 2.125693161 | 0.007438973 | 0.142616053 |
| GOTERM_BP_DIRECT | GO:0010812~negative regulation of cell-substrate adhesion | 5 | 0.462107209 | 0.007439108 | 0.142616053 |
| GOTERM_BP_DIRECT | GO:0006468~protein phosphorylation | 41 | 3.789279113 | 0.007719658 | 0.147207303 |
| GOTERM_BP_DIRECT | GO:0045332~phospholipid translocation | 6 | 0.554528651 | 0.007973233 | 0.1512383 |
| GOTERM_BP_DIRECT | GO:0046426~negative regulation of JAK-STAT cascade | 8 | 0.739371534 | 0.008136369 | 0.153520439 |
| GOTERM_BP_DIRECT | GO:0006461~protein complex assembly | 15 | 1.386321627 | 0.00822516 | 0.154383238 |
| GOTERM_BP_DIRECT | GO:0007157~heterophilic cell-cell adhesion via plasma membrane cell adhesion molecules | 9 | 0.831792976 | 0.008329777 | 0.155532562 |
| GOTERM_BP_DIRECT | GO:0048701~embryonic cranial skeleton morphogenesis | 7 | 0.646950092 | 0.008513102 | 0.158131973 |
| GOTERM_BP_DIRECT | GO:0010818~T cell chemotaxis | 4 | 0.369685767 | 0.009120603 | 0.165138189 |
| GOTERM_BP_DIRECT | GO:0032731~positive regulation of interleukin-1 beta production | 4 | 0.369685767 | 0.009120603 | 0.165138189 |
| GOTERM_BP_DIRECT | GO:0072075~metanephric mesenchyme development | 4 | 0.369685767 | 0.009120603 | 0.165138189 |
| GOTERM_BP_DIRECT | GO:0002467~germinal center formation | 4 | 0.369685767 | 0.009120603 | 0.165138189 |
| GOTERM_BP_DIRECT | GO:0071711~basement membrane organization | 4 | 0.369685767 | 0.009120603 | 0.165138189 |
| GOTERM_BP_DIRECT | GO:0048015~phosphatidylinositol-mediated signaling | 14 | 1.293900185 | 0.009354231 | 0.167661436 |
| GOTERM_BP_DIRECT | GO:0030036~actin cytoskeleton organization | 16 | 1.478743068 | 0.009542185 | 0.167661436 |
| GOTERM_BP_DIRECT | GO:0002042~cell migration involved in sprouting angiogenesis | 5 | 0.462107209 | 0.009678096 | 0.167661436 |
| GOTERM_BP_DIRECT | GO:0046677~response to antibiotic | 7 | 0.646950092 | 0.00996739 | 0.167661436 |
| GOTERM_BP_DIRECT | GO:0031663~lipopolysaccharide-mediated signaling pathway | 7 | 0.646950092 | 0.00996739 | 0.167661436 |
| GOTERM_BP_DIRECT | GO:0045907~positive regulation of vasoconstriction | 7 | 0.646950092 | 0.00996739 | 0.167661436 |
| GOTERM_BP_DIRECT | GO:2001202~negative regulation of transforming growth factor-beta secretion | 3 | 0.277264325 | 0.009989253 | 0.167661436 |
| GOTERM_BP_DIRECT | GO:0002374~cytokine secretion involved in immune response | 3 | 0.277264325 | 0.009989253 | 0.167661436 |
| GOTERM_BP_DIRECT | GO:1903375~facioacoustic ganglion development | 3 | 0.277264325 | 0.009989253 | 0.167661436 |
| GOTERM_BP_DIRECT | GO:0072277~metanephric glomerular capillary formation | 3 | 0.277264325 | 0.009989253 | 0.167661436 |
| GOTERM_BP_DIRECT | GO:0035583~sequestering of TGFbeta in extracellular matrix | 3 | 0.277264325 | 0.009989253 | 0.167661436 |
| GOTERM_BP_DIRECT | GO:0021943~formation of radial glial scaffolds | 3 | 0.277264325 | 0.009989253 | 0.167661436 |
| GOTERM_BP_DIRECT | GO:0021828~gonadotrophin-releasing hormone neuronal migration to the hypothalamus | 3 | 0.277264325 | 0.009989253 | 0.167661436 |
| GOTERM_BP_DIRECT | GO:0002291~T cell activation via T cell receptor contact with antigen bound to MHC molecule on antigen presenting cell | 3 | 0.277264325 | 0.009989253 | 0.167661436 |
| GOTERM_BP_DIRECT | GO:0009595~detection of biotic stimulus | 3 | 0.277264325 | 0.009989253 | 0.167661436 |
| GOTERM_BP_DIRECT | GO:0016525~negative regulation of angiogenesis | 10 | 0.924214418 | 0.010008242 | 0.167661436 |
| GOTERM_BP_DIRECT | GO:0051897~positive regulation of protein kinase B signaling | 12 | 1.109057301 | 0.010087037 | 0.168195473 |
| GOTERM_BP_DIRECT | GO:0060348~bone development | 8 | 0.739371534 | 0.010635624 | 0.175708347 |
| GOTERM_BP_DIRECT | GO:0045599~negative regulation of fat cell differentiation | 8 | 0.739371534 | 0.010635624 | 0.175708347 |
| GOTERM_BP_DIRECT | GO:0048870~cell motility | 6 | 0.554528651 | 0.011674962 | 0.191117534 |
| GOTERM_BP_DIRECT | GO:0045671~negative regulation of osteoclast differentiation | 6 | 0.554528651 | 0.011674962 | 0.191117534 |
| GOTERM_BP_DIRECT | GO:0000165~MAPK cascade | 26 | 2.402957486 | 0.012048314 | 0.196332745 |
| GOTERM_BP_DIRECT | GO:0030512~negative regulation of transforming growth factor beta receptor signaling pathway | 10 | 0.924214418 | 0.012243743 | 0.197938848 |
| GOTERM_BP_DIRECT | GO:0048566~embryonic digestive tract development | 5 | 0.462107209 | 0.012312514 | 0.197938848 |
| GOTERM_BP_DIRECT | GO:0051895~negative regulation of focal adhesion assembly | 5 | 0.462107209 | 0.012312514 | 0.197938848 |
| GOTERM_BP_DIRECT | GO:0032836~glomerular basement membrane development | 4 | 0.369685767 | 0.013087608 | 0.202146331 |
| GOTERM_BP_DIRECT | GO:0010863~positive regulation of phospholipase C activity | 4 | 0.369685767 | 0.013087608 | 0.202146331 |
| GOTERM_BP_DIRECT | GO:0060539~diaphragm development | 4 | 0.369685767 | 0.013087608 | 0.202146331 |
| GOTERM_BP_DIRECT | GO:0032330~regulation of chondrocyte differentiation | 4 | 0.369685767 | 0.013087608 | 0.202146331 |
| GOTERM_BP_DIRECT | GO:0021612~facial nerve structural organization | 4 | 0.369685767 | 0.013087608 | 0.202146331 |
| GOTERM_BP_DIRECT | GO:0035313~wound healing, spreading of epidermal cells | 4 | 0.369685767 | 0.013087608 | 0.202146331 |
| GOTERM_BP_DIRECT | GO:0035988~chondrocyte proliferation | 4 | 0.369685767 | 0.013087608 | 0.202146331 |
| GOTERM_BP_DIRECT | GO:0002544~chronic inflammatory response | 4 | 0.369685767 | 0.013087608 | 0.202146331 |
| GOTERM_BP_DIRECT | GO:2000810~regulation of bicellular tight junction assembly | 4 | 0.369685767 | 0.013087608 | 0.202146331 |
| GOTERM_BP_DIRECT | GO:0048146~positive regulation of fibroblast proliferation | 9 | 0.831792976 | 0.013138102 | 0.202146331 |
| GOTERM_BP_DIRECT | GO:0006469~negative regulation of protein kinase activity | 13 | 1.201478743 | 0.013369686 | 0.204830445 |
| GOTERM_BP_DIRECT | GO:0008217~regulation of blood pressure | 10 | 0.924214418 | 0.013491671 | 0.205819748 |
| GOTERM_BP_DIRECT | GO:0002040~sprouting angiogenesis | 6 | 0.554528651 | 0.013901974 | 0.211180415 |
| GOTERM_BP_DIRECT | GO:0035567~non-canonical Wnt signaling pathway | 5 | 0.462107209 | 0.0153645 | 0.227790015 |
| GOTERM_BP_DIRECT | GO:0030048~actin filament-based movement | 5 | 0.462107209 | 0.0153645 | 0.227790015 |
| GOTERM_BP_DIRECT | GO:0007178~transmembrane receptor protein serine/threonine kinase signaling pathway | 5 | 0.462107209 | 0.0153645 | 0.227790015 |
| GOTERM_BP_DIRECT | GO:0031589~cell-substrate adhesion | 5 | 0.462107209 | 0.0153645 | 0.227790015 |
| GOTERM_BP_DIRECT | GO:0002504~antigen processing and presentation of peptide or polysaccharide antigen via MHC class II | 5 | 0.462107209 | 0.0153645 | 0.227790015 |
| GOTERM_BP_DIRECT | GO:0030501~positive regulation of bone mineralization | 7 | 0.646950092 | 0.01537662 | 0.227790015 |
| GOTERM_BP_DIRECT | GO:0016337~single organismal cell-cell adhesion | 13 | 1.201478743 | 0.015502401 | 0.228708265 |
| GOTERM_BP_DIRECT | GO:0016049~cell growth | 9 | 0.831792976 | 0.016186602 | 0.237823636 |
| GOTERM_BP_DIRECT | GO:0007202~activation of phospholipase C activity | 6 | 0.554528651 | 0.016397589 | 0.239940224 |
| GOTERM_BP_DIRECT | GO:0042552~myelination | 8 | 0.739371534 | 0.017231065 | 0.249086166 |
| GOTERM_BP_DIRECT | GO:0010595~positive regulation of endothelial cell migration | 8 | 0.739371534 | 0.017231065 | 0.249086166 |
| GOTERM_BP_DIRECT | GO:0003151~outflow tract morphogenesis | 8 | 0.739371534 | 0.017231065 | 0.249086166 |
| GOTERM_BP_DIRECT | GO:0030177~positive regulation of Wnt signaling pathway | 7 | 0.646950092 | 0.017558406 | 0.250291749 |
| GOTERM_BP_DIRECT | GO:0002726~positive regulation of T cell cytokine production | 4 | 0.369685767 | 0.017888394 | 0.250291749 |
| GOTERM_BP_DIRECT | GO:0010820~positive regulation of T cell chemotaxis | 4 | 0.369685767 | 0.017888394 | 0.250291749 |
| GOTERM_BP_DIRECT | GO:0042116~macrophage activation | 4 | 0.369685767 | 0.017888394 | 0.250291749 |
| GOTERM_BP_DIRECT | GO:0002675~positive regulation of acute inflammatory response | 4 | 0.369685767 | 0.017888394 | 0.250291749 |
| GOTERM_BP_DIRECT | GO:0035414~negative regulation of catenin import into nucleus | 4 | 0.369685767 | 0.017888394 | 0.250291749 |
| GOTERM_BP_DIRECT | GO:0051272~positive regulation of cellular component movement | 4 | 0.369685767 | 0.017888394 | 0.250291749 |
| GOTERM_BP_DIRECT | GO:0048557~embryonic digestive tract morphogenesis | 5 | 0.462107209 | 0.018852874 | 0.250291749 |
| GOTERM_BP_DIRECT | GO:0030500~regulation of bone mineralization | 5 | 0.462107209 | 0.018852874 | 0.250291749 |
| GOTERM_BP_DIRECT | GO:0050870~positive regulation of T cell activation | 5 | 0.462107209 | 0.018852874 | 0.250291749 |
| GOTERM_BP_DIRECT | GO:0030225~macrophage differentiation | 5 | 0.462107209 | 0.018852874 | 0.250291749 |
| GOTERM_BP_DIRECT | GO:0042476~odontogenesis | 6 | 0.554528651 | 0.019173852 | 0.250291749 |
| GOTERM_BP_DIRECT | GO:0045629~negative regulation of T-helper 2 cell differentiation | 3 | 0.277264325 | 0.019199507 | 0.250291749 |
| GOTERM_BP_DIRECT | GO:0030260~entry into host cell | 3 | 0.277264325 | 0.019199507 | 0.250291749 |
| GOTERM_BP_DIRECT | GO:0030323~respiratory tube development | 3 | 0.277264325 | 0.019199507 | 0.250291749 |
| GOTERM_BP_DIRECT | GO:0060033~anatomical structure regression | 3 | 0.277264325 | 0.019199507 | 0.250291749 |
| GOTERM_BP_DIRECT | GO:1902285~semaphorin-plexin signaling pathway involved in neuron projection guidance | 3 | 0.277264325 | 0.019199507 | 0.250291749 |
| GOTERM_BP_DIRECT | GO:0048050~post-embryonic eye morphogenesis | 3 | 0.277264325 | 0.019199507 | 0.250291749 |
| GOTERM_BP_DIRECT | GO:0007442~hindgut morphogenesis | 3 | 0.277264325 | 0.019199507 | 0.250291749 |
| GOTERM_BP_DIRECT | GO:0036486~ventral trunk neural crest cell migration | 3 | 0.277264325 | 0.019199507 | 0.250291749 |
| GOTERM_BP_DIRECT | GO:0097491~sympathetic neuron projection guidance | 3 | 0.277264325 | 0.019199507 | 0.250291749 |
| GOTERM_BP_DIRECT | GO:0072672~neutrophil extravasation | 3 | 0.277264325 | 0.019199507 | 0.250291749 |
| GOTERM_BP_DIRECT | GO:0061333~renal tubule morphogenesis | 3 | 0.277264325 | 0.019199507 | 0.250291749 |
| GOTERM_BP_DIRECT | GO:0060841~venous blood vessel development | 3 | 0.277264325 | 0.019199507 | 0.250291749 |
| GOTERM_BP_DIRECT | GO:0021938~smoothened signaling pathway involved in regulation of cerebellar granule cell precursor cell proliferation | 3 | 0.277264325 | 0.019199507 | 0.250291749 |
| GOTERM_BP_DIRECT | GO:0097490~sympathetic neuron projection extension | 3 | 0.277264325 | 0.019199507 | 0.250291749 |
| GOTERM_BP_DIRECT | GO:0038110~interleukin-2-mediated signaling pathway | 3 | 0.277264325 | 0.019199507 | 0.250291749 |
| GOTERM_BP_DIRECT | GO:0001938~positive regulation of endothelial cell proliferation | 10 | 0.924214418 | 0.019437752 | 0.252479491 |
| GOTERM_BP_DIRECT | GO:0051259~protein oligomerization | 9 | 0.831792976 | 0.019715053 | 0.255156919 |
| GOTERM_BP_DIRECT | GO:0007162~negative regulation of cell adhesion | 7 | 0.646950092 | 0.019943453 | 0.257184454 |
| GOTERM_BP_DIRECT | GO:0035023~regulation of Rho protein signal transduction | 11 | 1.01663586 | 0.020270283 | 0.260462237 |
| GOTERM_BP_DIRECT | GO:0071300~cellular response to retinoic acid | 10 | 0.924214418 | 0.021180869 | 0.271190766 |
| GOTERM_BP_DIRECT | GO:0010862~positive regulation of pathway-restricted SMAD protein phosphorylation | 8 | 0.739371534 | 0.021427695 | 0.273374687 |
| GOTERM_BP_DIRECT | GO:0043406~positive regulation of MAP kinase activity | 9 | 0.831792976 | 0.021670472 | 0.274518174 |
| GOTERM_BP_DIRECT | GO:0009612~response to mechanical stimulus | 9 | 0.831792976 | 0.021670472 | 0.274518174 |
| GOTERM_BP_DIRECT | GO:0050690~regulation of defense response to virus by virus | 6 | 0.554528651 | 0.022241656 | 0.28076175 |
| GOTERM_BP_DIRECT | GO:0032332~positive regulation of chondrocyte differentiation | 5 | 0.462107209 | 0.02279311 | 0.286713328 |
| GOTERM_BP_DIRECT | GO:0050732~negative regulation of peptidyl-tyrosine phosphorylation | 4 | 0.369685767 | 0.023536797 | 0.289963632 |
| GOTERM_BP_DIRECT | GO:0033690~positive regulation of osteoblast proliferation | 4 | 0.369685767 | 0.023536797 | 0.289963632 |
| GOTERM_BP_DIRECT | GO:0032700~negative regulation of interleukin-17 production | 4 | 0.369685767 | 0.023536797 | 0.289963632 |
| GOTERM_BP_DIRECT | GO:0051764~actin crosslink formation | 4 | 0.369685767 | 0.023536797 | 0.289963632 |
| GOTERM_BP_DIRECT | GO:0046622~positive regulation of organ growth | 4 | 0.369685767 | 0.023536797 | 0.289963632 |
| GOTERM_BP_DIRECT | GO:0050868~negative regulation of T cell activation | 4 | 0.369685767 | 0.023536797 | 0.289963632 |
| GOTERM_BP_DIRECT | GO:0043433~negative regulation of sequence-specific DNA binding transcription factor activity | 9 | 0.831792976 | 0.023759049 | 0.290703719 |
| GOTERM_BP_DIRECT | GO:0050679~positive regulation of epithelial cell proliferation | 9 | 0.831792976 | 0.023759049 | 0.290703719 |
| GOTERM_BP_DIRECT | GO:0007596~blood coagulation | 19 | 1.756007394 | 0.023904709 | 0.291491093 |
| GOTERM_BP_DIRECT | GO:0001947~heart looping | 9 | 0.831792976 | 0.025984848 | 0.315781968 |
| GOTERM_BP_DIRECT | GO:0010506~regulation of autophagy | 8 | 0.739371534 | 0.02628472 | 0.318347027 |
| GOTERM_BP_DIRECT | GO:0043113~receptor clustering | 5 | 0.462107209 | 0.027197362 | 0.327189746 |
| GOTERM_BP_DIRECT | GO:0030101~natural killer cell activation | 5 | 0.462107209 | 0.027197362 | 0.327189746 |
| GOTERM_BP_DIRECT | GO:0001701~in utero embryonic development | 19 | 1.756007394 | 0.027567012 | 0.330527546 |
| GOTERM_BP_DIRECT | GO:0043123~positive regulation of I-kappaB kinase/NF-kappaB signaling | 17 | 1.57116451 | 0.028097656 | 0.334142193 |
| GOTERM_BP_DIRECT | GO:0010508~positive regulation of autophagy | 7 | 0.646950092 | 0.028391492 | 0.334142193 |
| GOTERM_BP_DIRECT | GO:0038083~peptidyl-tyrosine autophosphorylation | 7 | 0.646950092 | 0.028391492 | 0.334142193 |
| GOTERM_BP_DIRECT | GO:0048468~cell development | 7 | 0.646950092 | 0.028391492 | 0.334142193 |
| GOTERM_BP_DIRECT | GO:0043066~negative regulation of apoptotic process | 38 | 3.512014787 | 0.029418243 | 0.334142193 |
| GOTERM_BP_DIRECT | GO:0001822~kidney development | 11 | 1.01663586 | 0.029445309 | 0.334142193 |
| GOTERM_BP_DIRECT | GO:0042340~keratan sulfate catabolic process | 4 | 0.369685767 | 0.030034766 | 0.334142193 |
| GOTERM_BP_DIRECT | GO:0034329~cell junction assembly | 4 | 0.369685767 | 0.030034766 | 0.334142193 |
| GOTERM_BP_DIRECT | GO:2000279~negative regulation of DNA biosynthetic process | 4 | 0.369685767 | 0.030034766 | 0.334142193 |
| GOTERM_BP_DIRECT | GO:0001774~microglial cell activation | 4 | 0.369685767 | 0.030034766 | 0.334142193 |
| GOTERM_BP_DIRECT | GO:0045779~negative regulation of bone resorption | 4 | 0.369685767 | 0.030034766 | 0.334142193 |
| GOTERM_BP_DIRECT | GO:0035385~Roundabout signaling pathway | 4 | 0.369685767 | 0.030034766 | 0.334142193 |
| GOTERM_BP_DIRECT | GO:0030208~dermatan sulfate biosynthetic process | 4 | 0.369685767 | 0.030034766 | 0.334142193 |
| GOTERM_BP_DIRECT | GO:0001779~natural killer cell differentiation | 4 | 0.369685767 | 0.030034766 | 0.334142193 |
| GOTERM_BP_DIRECT | GO:0021696~cerebellar cortex morphogenesis | 3 | 0.277264325 | 0.030757859 | 0.334142193 |
| GOTERM_BP_DIRECT | GO:0010715~regulation of extracellular matrix disassembly | 3 | 0.277264325 | 0.030757859 | 0.334142193 |
| GOTERM_BP_DIRECT | GO:0010632~regulation of epithelial cell migration | 3 | 0.277264325 | 0.030757859 | 0.334142193 |
| GOTERM_BP_DIRECT | GO:0061551~trigeminal ganglion development | 3 | 0.277264325 | 0.030757859 | 0.334142193 |
| GOTERM_BP_DIRECT | GO:0032713~negative regulation of interleukin-4 production | 3 | 0.277264325 | 0.030757859 | 0.334142193 |
| GOTERM_BP_DIRECT | GO:0001915~negative regulation of T cell mediated cytotoxicity | 3 | 0.277264325 | 0.030757859 | 0.334142193 |
| GOTERM_BP_DIRECT | GO:0002634~regulation of germinal center formation | 3 | 0.277264325 | 0.030757859 | 0.334142193 |
| GOTERM_BP_DIRECT | GO:0045077~negative regulation of interferon-gamma biosynthetic process | 3 | 0.277264325 | 0.030757859 | 0.334142193 |
| GOTERM_BP_DIRECT | GO:1900227~positive regulation of NLRP3 inflammasome complex assembly | 3 | 0.277264325 | 0.030757859 | 0.334142193 |
| GOTERM_BP_DIRECT | GO:0070100~negative regulation of chemokine-mediated signaling pathway | 3 | 0.277264325 | 0.030757859 | 0.334142193 |
| GOTERM_BP_DIRECT | GO:1901166~neural crest cell migration involved in autonomic nervous system development | 3 | 0.277264325 | 0.030757859 | 0.334142193 |
| GOTERM_BP_DIRECT | GO:0071225~cellular response to muramyl dipeptide | 3 | 0.277264325 | 0.030757859 | 0.334142193 |
| GOTERM_BP_DIRECT | GO:0072540~T-helper 17 cell lineage commitment | 3 | 0.277264325 | 0.030757859 | 0.334142193 |
| GOTERM_BP_DIRECT | GO:0060666~dichotomous subdivision of terminal units involved in salivary gland branching | 3 | 0.277264325 | 0.030757859 | 0.334142193 |
| GOTERM_BP_DIRECT | GO:0072284~metanephric S-shaped body morphogenesis | 3 | 0.277264325 | 0.030757859 | 0.334142193 |
| GOTERM_BP_DIRECT | GO:0071800~podosome assembly | 3 | 0.277264325 | 0.030757859 | 0.334142193 |
| GOTERM_BP_DIRECT | GO:0022614~membrane to membrane docking | 3 | 0.277264325 | 0.030757859 | 0.334142193 |
| GOTERM_BP_DIRECT | GO:0071320~cellular response to cAMP | 8 | 0.739371534 | 0.031842329 | 0.343839607 |
| GOTERM_BP_DIRECT | GO:0034097~response to cytokine | 8 | 0.739371534 | 0.031842329 | 0.343839607 |
| GOTERM_BP_DIRECT | GO:0031954~positive regulation of protein autophosphorylation | 5 | 0.462107209 | 0.032074534 | 0.345306923 |
| GOTERM_BP_DIRECT | GO:0030509~BMP signaling pathway | 10 | 0.924214418 | 0.03405254 | 0.365504059 |
| GOTERM_BP_DIRECT | GO:0002548~monocyte chemotaxis | 7 | 0.646950092 | 0.035164115 | 0.374075231 |
| GOTERM_BP_DIRECT | GO:0001658~branching involved in ureteric bud morphogenesis | 7 | 0.646950092 | 0.035164115 | 0.374075231 |
| GOTERM_BP_DIRECT | GO:0022008~neurogenesis | 7 | 0.646950092 | 0.035164115 | 0.374075231 |
| GOTERM_BP_DIRECT | GO:0018108~peptidyl-tyrosine phosphorylation | 16 | 1.478743068 | 0.036308514 | 0.385106577 |
| GOTERM_BP_DIRECT | GO:0051926~negative regulation of calcium ion transport | 4 | 0.369685767 | 0.037374142 | 0.388950461 |
| GOTERM_BP_DIRECT | GO:1900025~negative regulation of substrate adhesion-dependent cell spreading | 4 | 0.369685767 | 0.037374142 | 0.388950461 |
| GOTERM_BP_DIRECT | GO:0010952~positive regulation of peptidase activity | 4 | 0.369685767 | 0.037374142 | 0.388950461 |
| GOTERM_BP_DIRECT | GO:0006518~peptide metabolic process | 4 | 0.369685767 | 0.037374142 | 0.388950461 |
| GOTERM_BP_DIRECT | GO:0050901~leukocyte tethering or rolling | 4 | 0.369685767 | 0.037374142 | 0.388950461 |
| GOTERM_BP_DIRECT | GO:0060413~atrial septum morphogenesis | 4 | 0.369685767 | 0.037374142 | 0.388950461 |
| GOTERM_BP_DIRECT | GO:0008037~cell recognition | 5 | 0.462107209 | 0.037430379 | 0.388950461 |
| GOTERM_BP_DIRECT | GO:0030155~regulation of cell adhesion | 7 | 0.646950092 | 0.038908277 | 0.401977445 |
| GOTERM_BP_DIRECT | GO:0031623~receptor internalization | 7 | 0.646950092 | 0.038908277 | 0.401977445 |
| GOTERM_BP_DIRECT | GO:0007200~phospholipase C-activating G-protein coupled receptor signaling pathway | 9 | 0.831792976 | 0.039300877 | 0.404866792 |
| GOTERM_BP_DIRECT | GO:0030316~osteoclast differentiation | 5 | 0.462107209 | 0.043267633 | 0.440491485 |
| GOTERM_BP_DIRECT | GO:0007179~transforming growth factor beta receptor signaling pathway | 11 | 1.01663586 | 0.043926308 | 0.440491485 |
| GOTERM_BP_DIRECT | GO:0050856~regulation of T cell receptor signaling pathway | 3 | 0.277264325 | 0.044356325 | 0.440491485 |
| GOTERM_BP_DIRECT | GO:0072307~regulation of metanephric nephron tubule epithelial cell differentiation | 3 | 0.277264325 | 0.044356325 | 0.440491485 |
| GOTERM_BP_DIRECT | GO:0038063~collagen-activated tyrosine kinase receptor signaling pathway | 3 | 0.277264325 | 0.044356325 | 0.440491485 |
| GOTERM_BP_DIRECT | GO:0045627~positive regulation of T-helper 1 cell differentiation | 3 | 0.277264325 | 0.044356325 | 0.440491485 |
| GOTERM_BP_DIRECT | GO:0032914~positive regulation of transforming growth factor beta1 production | 3 | 0.277264325 | 0.044356325 | 0.440491485 |
| GOTERM_BP_DIRECT | GO:0007044~cell-substrate junction assembly | 3 | 0.277264325 | 0.044356325 | 0.440491485 |
| GOTERM_BP_DIRECT | GO:0002830~positive regulation of type 2 immune response | 3 | 0.277264325 | 0.044356325 | 0.440491485 |
| GOTERM_BP_DIRECT | GO:0006929~substrate-dependent cell migration | 3 | 0.277264325 | 0.044356325 | 0.440491485 |
| GOTERM_BP_DIRECT | GO:0003416~endochondral bone growth | 3 | 0.277264325 | 0.044356325 | 0.440491485 |
| GOTERM_BP_DIRECT | GO:0060536~cartilage morphogenesis | 3 | 0.277264325 | 0.044356325 | 0.440491485 |
| GOTERM_BP_DIRECT | GO:0061299~retina vasculature morphogenesis in camera-type eye | 3 | 0.277264325 | 0.044356325 | 0.440491485 |
| GOTERM_BP_DIRECT | GO:0042733~embryonic digit morphogenesis | 8 | 0.739371534 | 0.045192658 | 0.44122864 |
| GOTERM_BP_DIRECT | GO:2000353~positive regulation of endothelial cell apoptotic process | 4 | 0.369685767 | 0.045538242 | 0.44122864 |
| GOTERM_BP_DIRECT | GO:0035589~G-protein coupled purinergic nucleotide receptor signaling pathway | 4 | 0.369685767 | 0.045538242 | 0.44122864 |
| GOTERM_BP_DIRECT | GO:0042554~superoxide anion generation | 4 | 0.369685767 | 0.045538242 | 0.44122864 |
| GOTERM_BP_DIRECT | GO:0030207~chondroitin sulfate catabolic process | 4 | 0.369685767 | 0.045538242 | 0.44122864 |
| GOTERM_BP_DIRECT | GO:0030833~regulation of actin filament polymerization | 4 | 0.369685767 | 0.045538242 | 0.44122864 |
| GOTERM_BP_DIRECT | GO:0030889~negative regulation of B cell proliferation | 4 | 0.369685767 | 0.045538242 | 0.44122864 |
| GOTERM_BP_DIRECT | GO:0014912~negative regulation of smooth muscle cell migration | 4 | 0.369685767 | 0.045538242 | 0.44122864 |
| GOTERM_BP_DIRECT | GO:1902043~positive regulation of extrinsic apoptotic signaling pathway via death domain receptors | 4 | 0.369685767 | 0.045538242 | 0.44122864 |
| GOTERM_BP_DIRECT | GO:0006508~proteolysis | 40 | 3.696857671 | 0.045675084 | 0.441361657 |
| GOTERM_BP_DIRECT | GO:0001755~neural crest cell migration | 7 | 0.646950092 | 0.047131802 | 0.45145639 |
| GOTERM_BP_DIRECT | GO:0050919~negative chemotaxis | 6 | 0.554528651 | 0.047223472 | 0.45145639 |
| GOTERM_BP_DIRECT | GO:0043588~skin development | 6 | 0.554528651 | 0.047223472 | 0.45145639 |
| GOTERM_BP_DIRECT | GO:0048286~lung alveolus development | 6 | 0.554528651 | 0.047223472 | 0.45145639 |
| GOTERM_BP_DIRECT | GO:0043524~negative regulation of neuron apoptotic process | 14 | 1.293900185 | 0.047456502 | 0.452477554 |
| GOTERM_BP_DIRECT | GO:0006959~humoral immune response | 8 | 0.739371534 | 0.049015232 | 0.466099751 |
| GOTERM_BP_DIRECT | GO:0030099~myeloid cell differentiation | 5 | 0.462107209 | 0.049586167 | 0.466578502 |
| GOTERM_BP_DIRECT | GO:0045880~positive regulation of smoothened signaling pathway | 5 | 0.462107209 | 0.049586167 | 0.466578502 |
| GOTERM_BP_DIRECT | GO:0009954~proximal/distal pattern formation | 5 | 0.462107209 | 0.049586167 | 0.466578502 |
| GOTERM_BP_DIRECT | GO:0030866~cortical actin cytoskeleton organization | 5 | 0.462107209 | 0.049586167 | 0.466578502 |
| GOTERM_BP_DIRECT | GO:0072593~reactive oxygen species metabolic process | 6 | 0.554528651 | 0.05253128 | 0.491709243 |
| GOTERM_BP_DIRECT | GO:0006911~phagocytosis, engulfment | 6 | 0.554528651 | 0.05253128 | 0.491709243 |
| GOTERM_BP_DIRECT | GO:0033138~positive regulation of peptidyl-serine phosphorylation | 9 | 0.831792976 | 0.052761719 | 0.492580109 |
| GOTERM_BP_DIRECT | GO:0030308~negative regulation of cell growth | 13 | 1.201478743 | 0.052984826 | 0.493378186 |
| GOTERM_BP_DIRECT | GO:0035024~negative regulation of Rho protein signal transduction | 4 | 0.369685767 | 0.054503278 | 0.503593434 |
| GOTERM_BP_DIRECT | GO:0010633~negative regulation of epithelial cell migration | 4 | 0.369685767 | 0.054503278 | 0.503593434 |
| GOTERM_BP_DIRECT | GO:0030502~negative regulation of bone mineralization | 4 | 0.369685767 | 0.054503278 | 0.503593434 |
| GOTERM_BP_DIRECT | GO:0031532~actin cytoskeleton reorganization | 7 | 0.646950092 | 0.056353601 | 0.516965768 |
| GOTERM_BP_DIRECT | GO:0060976~coronary vasculature development | 5 | 0.462107209 | 0.056383156 | 0.516965768 |
| GOTERM_BP_DIRECT | GO:0007569~cell aging | 5 | 0.462107209 | 0.056383156 | 0.516965768 |
| GOTERM_BP_DIRECT | GO:0071407~cellular response to organic cyclic compound | 8 | 0.739371534 | 0.057259219 | 0.523658926 |
| GOTERM_BP_DIRECT | GO:0007040~lysosome organization | 6 | 0.554528651 | 0.058172067 | 0.529896663 |
| GOTERM_BP_DIRECT | GO:0060840~artery development | 3 | 0.277264325 | 0.059714994 | 0.529896663 |
| GOTERM_BP_DIRECT | GO:1901222~regulation of NIK/NF-kappaB signaling | 3 | 0.277264325 | 0.059714994 | 0.529896663 |
| GOTERM_BP_DIRECT | GO:0030854~positive regulation of granulocyte differentiation | 3 | 0.277264325 | 0.059714994 | 0.529896663 |
| GOTERM_BP_DIRECT | GO:0060710~chorio-allantoic fusion | 3 | 0.277264325 | 0.059714994 | 0.529896663 |
| GOTERM_BP_DIRECT | GO:0050798~activated T cell proliferation | 3 | 0.277264325 | 0.059714994 | 0.529896663 |
| GOTERM_BP_DIRECT | GO:0032609~interferon-gamma production | 3 | 0.277264325 | 0.059714994 | 0.529896663 |
| GOTERM_BP_DIRECT | GO:0045359~positive regulation of interferon-beta biosynthetic process | 3 | 0.277264325 | 0.059714994 | 0.529896663 |
| GOTERM_BP_DIRECT | GO:0032736~positive regulation of interleukin-13 production | 3 | 0.277264325 | 0.059714994 | 0.529896663 |
| GOTERM_BP_DIRECT | GO:0050869~negative regulation of B cell activation | 3 | 0.277264325 | 0.059714994 | 0.529896663 |
| GOTERM_BP_DIRECT | GO:0072112~glomerular visceral epithelial cell differentiation | 3 | 0.277264325 | 0.059714994 | 0.529896663 |
| GOTERM_BP_DIRECT | GO:0034616~response to laminar fluid shear stress | 3 | 0.277264325 | 0.059714994 | 0.529896663 |
| GOTERM_BP_DIRECT | GO:0050830~defense response to Gram-positive bacterium | 10 | 0.924214418 | 0.062089974 | 0.549611248 |
| GOTERM_BP_DIRECT | GO:0007568~aging | 16 | 1.478743068 | 0.062688545 | 0.553542943 |
| GOTERM_BP_DIRECT | GO:0048843~negative regulation of axon extension involved in axon guidance | 5 | 0.462107209 | 0.06365326 | 0.557624688 |
| GOTERM_BP_DIRECT | GO:0032757~positive regulation of interleukin-8 production | 5 | 0.462107209 | 0.06365326 | 0.557624688 |
| GOTERM_BP_DIRECT | GO:0051928~positive regulation of calcium ion transport | 5 | 0.462107209 | 0.06365326 | 0.557624688 |
| GOTERM_BP_DIRECT | GO:0048813~dendrite morphogenesis | 6 | 0.554528651 | 0.064145287 | 0.557624688 |
| GOTERM_BP_DIRECT | GO:0048593~camera-type eye morphogenesis | 4 | 0.369685767 | 0.064239608 | 0.557624688 |
| GOTERM_BP_DIRECT | GO:0032924~activin receptor signaling pathway | 4 | 0.369685767 | 0.064239608 | 0.557624688 |
| GOTERM_BP_DIRECT | GO:0061036~positive regulation of cartilage development | 4 | 0.369685767 | 0.064239608 | 0.557624688 |
| GOTERM_BP_DIRECT | GO:0008283~cell proliferation | 30 | 2.772643253 | 0.064535017 | 0.557889025 |
| GOTERM_BP_DIRECT | GO:0006367~transcription initiation from RNA polymerase II promoter | 15 | 1.386321627 | 0.064581296 | 0.557889025 |
| GOTERM_BP_DIRECT | GO:0001933~negative regulation of protein phosphorylation | 8 | 0.739371534 | 0.066311407 | 0.57028388 |
| GOTERM_BP_DIRECT | GO:0006897~endocytosis | 14 | 1.293900185 | 0.066334276 | 0.57028388 |
| GOTERM_BP_DIRECT | GO:0007584~response to nutrient | 9 | 0.831792976 | 0.068869182 | 0.590660329 |
| GOTERM_BP_DIRECT | GO:0060395~SMAD protein signal transduction | 8 | 0.739371534 | 0.071142636 | 0.607907076 |
| GOTERM_BP_DIRECT | GO:0014065~phosphatidylinositol 3-kinase signaling | 5 | 0.462107209 | 0.071388809 | 0.607907076 |
| GOTERM_BP_DIRECT | GO:0033077~T cell differentiation in thymus | 5 | 0.462107209 | 0.071388809 | 0.607907076 |
| GOTERM_BP_DIRECT | GO:0002076~osteoblast development | 4 | 0.369685767 | 0.074712846 | 0.616940559 |
| GOTERM_BP_DIRECT | GO:0030224~monocyte differentiation | 4 | 0.369685767 | 0.074712846 | 0.616940559 |
| GOTERM_BP_DIRECT | GO:0048514~blood vessel morphogenesis | 4 | 0.369685767 | 0.074712846 | 0.616940559 |
| GOTERM_BP_DIRECT | GO:2000811~negative regulation of anoikis | 4 | 0.369685767 | 0.074712846 | 0.616940559 |
| GOTERM_BP_DIRECT | GO:0051893~regulation of focal adhesion assembly | 4 | 0.369685767 | 0.074712846 | 0.616940559 |
| GOTERM_BP_DIRECT | GO:0050727~regulation of inflammatory response | 8 | 0.739371534 | 0.076177535 | 0.616940559 |
| GOTERM_BP_DIRECT | GO:0031659~positive regulation of cyclin-dependent protein serine/threonine kinase activity involved in G1/S transition of mitotic cell cycle | 3 | 0.277264325 | 0.07657979 | 0.616940559 |
| GOTERM_BP_DIRECT | GO:0050863~regulation of T cell activation | 3 | 0.277264325 | 0.07657979 | 0.616940559 |
| GOTERM_BP_DIRECT | GO:0001766~membrane raft polarization | 3 | 0.277264325 | 0.07657979 | 0.616940559 |
| GOTERM_BP_DIRECT | GO:0045416~positive regulation of interleukin-8 biosynthetic process | 3 | 0.277264325 | 0.07657979 | 0.616940559 |
| GOTERM_BP_DIRECT | GO:1902715~positive regulation of interferon-gamma secretion | 3 | 0.277264325 | 0.07657979 | 0.616940559 |
| GOTERM_BP_DIRECT | GO:0031666~positive regulation of lipopolysaccharide-mediated signaling pathway | 3 | 0.277264325 | 0.07657979 | 0.616940559 |
| GOTERM_BP_DIRECT | GO:0045630~positive regulation of T-helper 2 cell differentiation | 3 | 0.277264325 | 0.07657979 | 0.616940559 |
| GOTERM_BP_DIRECT | GO:0060346~bone trabecula formation | 3 | 0.277264325 | 0.07657979 | 0.616940559 |
| GOTERM_BP_DIRECT | GO:0019064~fusion of virus membrane with host plasma membrane | 3 | 0.277264325 | 0.07657979 | 0.616940559 |
| GOTERM_BP_DIRECT | GO:0014902~myotube differentiation | 3 | 0.277264325 | 0.07657979 | 0.616940559 |
| GOTERM_BP_DIRECT | GO:0033629~negative regulation of cell adhesion mediated by integrin | 3 | 0.277264325 | 0.07657979 | 0.616940559 |
| GOTERM_BP_DIRECT | GO:0002456~T cell mediated immunity | 3 | 0.277264325 | 0.07657979 | 0.616940559 |
| GOTERM_BP_DIRECT | GO:2000643~positive regulation of early endosome to late endosome transport | 3 | 0.277264325 | 0.07657979 | 0.616940559 |
| GOTERM_BP_DIRECT | GO:0001913~T cell mediated cytotoxicity | 3 | 0.277264325 | 0.07657979 | 0.616940559 |
| GOTERM_BP_DIRECT | GO:2000020~positive regulation of male gonad development | 3 | 0.277264325 | 0.07657979 | 0.616940559 |
| GOTERM_BP_DIRECT | GO:0060231~mesenchymal to epithelial transition | 3 | 0.277264325 | 0.07657979 | 0.616940559 |
| GOTERM_BP_DIRECT | GO:0001955~blood vessel maturation | 3 | 0.277264325 | 0.07657979 | 0.616940559 |
| GOTERM_BP_DIRECT | GO:0045410~positive regulation of interleukin-6 biosynthetic process | 3 | 0.277264325 | 0.07657979 | 0.616940559 |
| GOTERM_BP_DIRECT | GO:0007519~skeletal muscle tissue development | 7 | 0.646950092 | 0.077828871 | 0.625075024 |
| GOTERM_BP_DIRECT | GO:0006816~calcium ion transport | 9 | 0.831792976 | 0.077938225 | 0.625075024 |
| GOTERM_BP_DIRECT | GO:0043392~negative regulation of DNA binding | 5 | 0.462107209 | 0.079580001 | 0.633987339 |
| GOTERM_BP_DIRECT | GO:0051482~positive regulation of cytosolic calcium ion concentration involved in phospholipase C-activating G-protein coupled signaling pathway | 5 | 0.462107209 | 0.079580001 | 0.633987339 |
| GOTERM_BP_DIRECT | GO:0040007~growth | 5 | 0.462107209 | 0.079580001 | 0.633987339 |
| GOTERM_BP_DIRECT | GO:0006898~receptor-mediated endocytosis | 17 | 1.57116451 | 0.082625709 | 0.656791944 |
| GOTERM_BP_DIRECT | GO:0071333~cellular response to glucose stimulus | 7 | 0.646950092 | 0.083827115 | 0.663593121 |
| GOTERM_BP_DIRECT | GO:0030326~embryonic limb morphogenesis | 6 | 0.554528651 | 0.084036618 | 0.663593121 |
| GOTERM_BP_DIRECT | GO:0030819~positive regulation of cAMP biosynthetic process | 6 | 0.554528651 | 0.084036618 | 0.663593121 |
| GOTERM_BP_DIRECT | GO:0016055~Wnt signaling pathway | 17 | 1.57116451 | 0.085683743 | 0.666444053 |
| GOTERM_BP_DIRECT | GO:0001935~endothelial cell proliferation | 4 | 0.369685767 | 0.08588484 | 0.666444053 |
| GOTERM_BP_DIRECT | GO:2000249~regulation of actin cytoskeleton reorganization | 4 | 0.369685767 | 0.08588484 | 0.666444053 |
| GOTERM_BP_DIRECT | GO:0001782~B cell homeostasis | 4 | 0.369685767 | 0.08588484 | 0.666444053 |
| GOTERM_BP_DIRECT | GO:0036120~cellular response to platelet-derived growth factor stimulus | 4 | 0.369685767 | 0.08588484 | 0.666444053 |
| GOTERM_BP_DIRECT | GO:0036342~post-anal tail morphogenesis | 4 | 0.369685767 | 0.08588484 | 0.666444053 |
| GOTERM_BP_DIRECT | GO:0045954~positive regulation of natural killer cell mediated cytotoxicity | 4 | 0.369685767 | 0.08588484 | 0.666444053 |
| GOTERM_BP_DIRECT | GO:0045089~positive regulation of innate immune response | 4 | 0.369685767 | 0.08588484 | 0.666444053 |
| GOTERM_BP_DIRECT | GO:0046330~positive regulation of JNK cascade | 8 | 0.739371534 | 0.086856558 | 0.672528637 |
| GOTERM_BP_DIRECT | GO:0001937~negative regulation of endothelial cell proliferation | 5 | 0.462107209 | 0.088215092 | 0.675750228 |
| GOTERM_BP_DIRECT | GO:0048873~homeostasis of number of cells within a tissue | 5 | 0.462107209 | 0.088215092 | 0.675750228 |
| GOTERM_BP_DIRECT | GO:0048008~platelet-derived growth factor receptor signaling pathway | 5 | 0.462107209 | 0.088215092 | 0.675750228 |
| GOTERM_BP_DIRECT | GO:0043542~endothelial cell migration | 5 | 0.462107209 | 0.088215092 | 0.675750228 |
| GOTERM_BP_DIRECT | GO:0048662~negative regulation of smooth muscle cell proliferation | 5 | 0.462107209 | 0.088215092 | 0.675750228 |
| GOTERM_BP_DIRECT | GO:0001764~neuron migration | 11 | 1.01663586 | 0.090084406 | 0.688598286 |
| GOTERM_BP_DIRECT | GO:0070527~platelet aggregation | 6 | 0.554528651 | 0.091311143 | 0.695011563 |
| GOTERM_BP_DIRECT | GO:0032526~response to retinoic acid | 6 | 0.554528651 | 0.091311143 | 0.695011563 |
| GOTERM_BP_DIRECT | GO:0001960~negative regulation of cytokine-mediated signaling pathway | 3 | 0.277264325 | 0.094720414 | 0.701596454 |
| GOTERM_BP_DIRECT | GO:0070206~protein trimerization | 3 | 0.277264325 | 0.094720414 | 0.701596454 |
| GOTERM_BP_DIRECT | GO:0071404~cellular response to low-density lipoprotein particle stimulus | 3 | 0.277264325 | 0.094720414 | 0.701596454 |
| GOTERM_BP_DIRECT | GO:0061549~sympathetic ganglion development | 3 | 0.277264325 | 0.094720414 | 0.701596454 |
| GOTERM_BP_DIRECT | GO:0007494~midgut development | 3 | 0.277264325 | 0.094720414 | 0.701596454 |
| GOTERM_BP_DIRECT | GO:0048245~eosinophil chemotaxis | 3 | 0.277264325 | 0.094720414 | 0.701596454 |
| GOTERM_BP_DIRECT | GO:0045198~establishment of epithelial cell apical/basal polarity | 3 | 0.277264325 | 0.094720414 | 0.701596454 |
| GOTERM_BP_DIRECT | GO:0021785~branchiomotor neuron axon guidance | 3 | 0.277264325 | 0.094720414 | 0.701596454 |
| GOTERM_BP_DIRECT | GO:0002237~response to molecule of bacterial origin | 3 | 0.277264325 | 0.094720414 | 0.701596454 |
| GOTERM_BP_DIRECT | GO:0034405~response to fluid shear stress | 3 | 0.277264325 | 0.094720414 | 0.701596454 |
| GOTERM_BP_DIRECT | GO:0060340~positive regulation of type I interferon-mediated signaling pathway | 3 | 0.277264325 | 0.094720414 | 0.701596454 |
| GOTERM_BP_DIRECT | GO:0021675~nerve development | 3 | 0.277264325 | 0.094720414 | 0.701596454 |
| GOTERM_BP_DIRECT | GO:0050764~regulation of phagocytosis | 3 | 0.277264325 | 0.094720414 | 0.701596454 |
| GOTERM_BP_DIRECT | GO:0021915~neural tube development | 5 | 0.462107209 | 0.097280594 | 0.709122723 |
| GOTERM_BP_DIRECT | GO:0044344~cellular response to fibroblast growth factor stimulus | 5 | 0.462107209 | 0.097280594 | 0.709122723 |
| GOTERM_BP_DIRECT | GO:0071773~cellular response to BMP stimulus | 5 | 0.462107209 | 0.097280594 | 0.709122723 |
| GOTERM_BP_DIRECT | GO:0042098~T cell proliferation | 4 | 0.369685767 | 0.09771454 | 0.709122723 |
| GOTERM_BP_DIRECT | GO:0021772~olfactory bulb development | 4 | 0.369685767 | 0.09771454 | 0.709122723 |
| GOTERM_BP_DIRECT | GO:0045879~negative regulation of smoothened signaling pathway | 4 | 0.369685767 | 0.09771454 | 0.709122723 |
| GOTERM_BP_DIRECT | GO:0043536~positive regulation of blood vessel endothelial cell migration | 4 | 0.369685767 | 0.09771454 | 0.709122723 |
| GOTERM_BP_DIRECT | GO:0046579~positive regulation of Ras protein signal transduction | 4 | 0.369685767 | 0.09771454 | 0.709122723 |
| GOTERM_BP_DIRECT | GO:0008589~regulation of smoothened signaling pathway | 4 | 0.369685767 | 0.09771454 | 0.709122723 |
| GOTERM_BP_DIRECT | GO:0043011~myeloid dendritic cell differentiation | 4 | 0.369685767 | 0.09771454 | 0.709122723 |
| GOTERM_BP_DIRECT | GO:0035264~multicellular organism growth | 9 | 0.831792976 | 0.098112476 | 0.710572177 |
| GOTERM_BP_DIRECT | GO:0007566~embryo implantation | 6 | 0.554528651 | 0.098898579 | 0.71482138 |
| GOTERM_MF_DIRECT | GO:0005201~extracellular matrix structural constituent | 30 | 2.772643253 | 5.29E-19 | 4.66E-16 |
| GOTERM_MF_DIRECT | GO:0004872~receptor activity | 45 | 4.15896488 | 1.25E-13 | 5.50E-11 |
| GOTERM_MF_DIRECT | GO:0005178~integrin binding | 28 | 2.58780037 | 2.28E-11 | 6.70E-09 |
| GOTERM_MF_DIRECT | GO:0005518~collagen binding | 21 | 1.940850277 | 5.30E-11 | 1.17E-08 |
| GOTERM_MF_DIRECT | GO:0008201~heparin binding | 32 | 2.957486137 | 1.71E-09 | 3.00E-07 |
| GOTERM_MF_DIRECT | GO:0003779~actin binding | 44 | 4.066543438 | 2.23E-09 | 3.27E-07 |
| GOTERM_MF_DIRECT | GO:0050840~extracellular matrix binding | 13 | 1.201478743 | 5.15E-09 | 6.49E-07 |
| GOTERM_MF_DIRECT | GO:0015026~coreceptor activity | 13 | 1.201478743 | 3.73E-08 | 4.11E-06 |
| GOTERM_MF_DIRECT | GO:0005509~calcium ion binding | 78 | 7.208872458 | 5.77E-08 | 5.65E-06 |
| GOTERM_MF_DIRECT | GO:0001968~fibronectin binding | 12 | 1.109057301 | 6.96E-08 | 6.13E-06 |
| GOTERM_MF_DIRECT | GO:0005515~protein binding | 581 | 53.69685767 | 1.06E-07 | 8.51E-06 |
| GOTERM_MF_DIRECT | GO:0048407~platelet-derived growth factor binding | 8 | 0.739371534 | 5.22E-07 | 3.84E-05 |
| GOTERM_MF_DIRECT | GO:0004222~metalloendopeptidase activity | 21 | 1.940850277 | 5.60E-06 | 3.79E-04 |
| GOTERM_MF_DIRECT | GO:0017124~SH3 domain binding | 21 | 1.940850277 | 1.25E-05 | 7.88E-04 |
| GOTERM_MF_DIRECT | GO:0002020~protease binding | 19 | 1.756007394 | 1.47E-05 | 8.66E-04 |
| GOTERM_MF_DIRECT | GO:0001618~virus receptor activity | 15 | 1.386321627 | 3.43E-05 | 0.001890743 |
| GOTERM_MF_DIRECT | GO:0005096~GTPase activator activity | 34 | 3.14232902 | 5.90E-05 | 0.003057928 |
| GOTERM_MF_DIRECT | GO:0004888~transmembrane signaling receptor activity | 28 | 2.58780037 | 9.16E-05 | 0.00448114 |
| GOTERM_MF_DIRECT | GO:0016176~superoxide-generating NADPH oxidase activator activity | 6 | 0.554528651 | 1.19E-04 | 0.005288366 |
| GOTERM_MF_DIRECT | GO:0046332~SMAD binding | 11 | 1.01663586 | 1.20E-04 | 0.005288366 |
| GOTERM_MF_DIRECT | GO:0050431~transforming growth factor beta binding | 7 | 0.646950092 | 1.67E-04 | 0.006563545 |
| GOTERM_MF_DIRECT | GO:0046934~phosphatidylinositol-4,5-bisphosphate 3-kinase activity | 13 | 1.201478743 | 1.71E-04 | 0.006563545 |
| GOTERM_MF_DIRECT | GO:0050839~cell adhesion molecule binding | 13 | 1.201478743 | 1.71E-04 | 0.006563545 |
| GOTERM_MF_DIRECT | GO:0008237~metallopeptidase activity | 15 | 1.386321627 | 1.81E-04 | 0.006626816 |
| GOTERM_MF_DIRECT | GO:0016175~superoxide-generating NADPH oxidase activity | 6 | 0.554528651 | 2.08E-04 | 0.007044006 |
| GOTERM_MF_DIRECT | GO:0043394~proteoglycan binding | 6 | 0.554528651 | 2.08E-04 | 0.007044006 |
| GOTERM_MF_DIRECT | GO:0043325~phosphatidylinositol-3,4-bisphosphate binding | 8 | 0.739371534 | 3.76E-04 | 0.012253052 |
| GOTERM_MF_DIRECT | GO:0019955~cytokine binding | 7 | 0.646950092 | 4.87E-04 | 0.01531704 |
| GOTERM_MF_DIRECT | GO:0051015~actin filament binding | 19 | 1.756007394 | 5.12E-04 | 0.015560669 |
| GOTERM_MF_DIRECT | GO:0005102~receptor binding | 37 | 3.419593346 | 5.44E-04 | 0.015966601 |
| GOTERM_MF_DIRECT | GO:0017147~Wnt-protein binding | 8 | 0.739371534 | 0.001521943 | 0.043252646 |
| GOTERM_MF_DIRECT | GO:0001078~transcriptional repressor activity, RNA polymerase II core promoter proximal region sequence-specific binding | 16 | 1.478743068 | 0.001595035 | 0.0439133 |
| GOTERM_MF_DIRECT | GO:0001875~lipopolysaccharide receptor activity | 4 | 0.369685767 | 0.001701809 | 0.044096862 |
| GOTERM_MF_DIRECT | GO:0030023~extracellular matrix constituent conferring elasticity | 4 | 0.369685767 | 0.001701809 | 0.044096862 |
| GOTERM_MF_DIRECT | GO:0043236~laminin binding | 7 | 0.646950092 | 0.002367904 | 0.059603535 |
| GOTERM_MF_DIRECT | GO:0004860~protein kinase inhibitor activity | 10 | 0.924214418 | 0.002487562 | 0.060078924 |
| GOTERM_MF_DIRECT | GO:0019864~IgG binding | 5 | 0.462107209 | 0.002523178 | 0.060078924 |
| GOTERM_MF_DIRECT | GO:0005520~insulin-like growth factor binding | 6 | 0.554528651 | 0.002757516 | 0.063930842 |
| GOTERM_MF_DIRECT | GO:0003714~transcription corepressor activity | 23 | 2.125693161 | 0.002936536 | 0.066335605 |
| GOTERM_MF_DIRECT | GO:0042608~T cell receptor binding | 4 | 0.369685767 | 0.003259126 | 0.071782247 |
| GOTERM_MF_DIRECT | GO:0019838~growth factor binding | 7 | 0.646950092 | 0.003589948 | 0.074064435 |
| GOTERM_MF_DIRECT | GO:0001948~glycoprotein binding | 11 | 1.01663586 | 0.003599461 | 0.074064435 |
| GOTERM_MF_DIRECT | GO:0016493~C-C chemokine receptor activity | 5 | 0.462107209 | 0.00361495 | 0.074064435 |
| GOTERM_MF_DIRECT | GO:0030246~carbohydrate binding | 22 | 2.033271719 | 0.004126988 | 0.082633547 |
| GOTERM_MF_DIRECT | GO:0017048~Rho GTPase binding | 7 | 0.646950092 | 0.004351937 | 0.085201259 |
| GOTERM_MF_DIRECT | GO:0005547~phosphatidylinositol-3,4,5-trisphosphate binding | 8 | 0.739371534 | 0.005159783 | 0.098821064 |
| GOTERM_MF_DIRECT | GO:0004871~signal transducer activity | 22 | 2.033271719 | 0.006519367 | 0.122203461 |
| GOTERM_MF_DIRECT | GO:0005161~platelet-derived growth factor receptor binding | 5 | 0.462107209 | 0.008691189 | 0.158889741 |
| GOTERM_MF_DIRECT | GO:0035091~phosphatidylinositol binding | 12 | 1.109057301 | 0.008837227 | 0.158889741 |
| GOTERM_MF_DIRECT | GO:0004992~platelet activating factor receptor activity | 3 | 0.277264325 | 0.00940223 | 0.159295474 |
| GOTERM_MF_DIRECT | GO:0005019~platelet-derived growth factor beta-receptor activity | 3 | 0.277264325 | 0.00940223 | 0.159295474 |
| GOTERM_MF_DIRECT | GO:0004720~protein-lysine 6-oxidase activity | 3 | 0.277264325 | 0.00940223 | 0.159295474 |
| GOTERM_MF_DIRECT | GO:0042802~identical protein binding | 59 | 5.452865065 | 0.011315545 | 0.188094248 |
| GOTERM_MF_DIRECT | GO:0001206~transcriptional repressor activity, RNA polymerase II distal enhancer sequence-specific binding | 4 | 0.369685767 | 0.012027655 | 0.196228959 |
| GOTERM_MF_DIRECT | GO:0003705~transcription factor activity, RNA polymerase II distal enhancer sequence-specific binding | 10 | 0.924214418 | 0.012314665 | 0.197258548 |
| GOTERM_MF_DIRECT | GO:0008233~peptidase activity | 12 | 1.109057301 | 0.013336375 | 0.209809751 |
| GOTERM_MF_DIRECT | GO:0004950~chemokine receptor activity | 5 | 0.462107209 | 0.01383708 | 0.213867845 |
| GOTERM_MF_DIRECT | GO:0005080~protein kinase C binding | 8 | 0.739371534 | 0.014757637 | 0.224163416 |
| GOTERM_MF_DIRECT | GO:0005109~frizzled binding | 7 | 0.646950092 | 0.015278394 | 0.228140093 |
| GOTERM_MF_DIRECT | GO:0000977~RNA polymerase II regulatory region sequence-specific DNA binding | 21 | 1.940850277 | 0.015785771 | 0.231787738 |
| GOTERM_MF_DIRECT | GO:0016504~peptidase activator activity | 4 | 0.369685767 | 0.016461827 | 0.237751961 |
| GOTERM_MF_DIRECT | GO:0042056~chemoattractant activity | 6 | 0.554528651 | 0.016981574 | 0.237767485 |
| GOTERM_MF_DIRECT | GO:0005539~glycosaminoglycan binding | 5 | 0.462107209 | 0.017002669 | 0.237767485 |
| GOTERM_MF_DIRECT | GO:0004982~N-formyl peptide receptor activity | 3 | 0.277264325 | 0.018093463 | 0.249067833 |
| GOTERM_MF_DIRECT | GO:0005044~scavenger receptor activity | 8 | 0.739371534 | 0.018407096 | 0.249486942 |
| GOTERM_MF_DIRECT | GO:0051018~protein kinase A binding | 5 | 0.462107209 | 0.020585119 | 0.274780143 |
| GOTERM_MF_DIRECT | GO:0005543~phospholipid binding | 11 | 1.01663586 | 0.024453469 | 0.321544872 |
| GOTERM_MF_DIRECT | GO:0042834~peptidoglycan binding | 4 | 0.369685767 | 0.027713444 | 0.345509549 |
| GOTERM_MF_DIRECT | GO:0098639~collagen binding involved in cell-matrix adhesion | 3 | 0.277264325 | 0.029021233 | 0.345509549 |
| GOTERM_MF_DIRECT | GO:0016641~oxidoreductase activity, acting on the CH-NH2 group of donors, oxygen as acceptor | 3 | 0.277264325 | 0.029021233 | 0.345509549 |
| GOTERM_MF_DIRECT | GO:0035004~phosphatidylinositol 3-kinase activity | 3 | 0.277264325 | 0.029021233 | 0.345509549 |
| GOTERM_MF_DIRECT | GO:0047238~glucuronosyl-N-acetylgalactosaminyl-proteoglycan 4-beta-N-acetylgalactosaminyltransferase activity | 3 | 0.277264325 | 0.029021233 | 0.345509549 |
| GOTERM_MF_DIRECT | GO:0038085~vascular endothelial growth factor binding | 3 | 0.277264325 | 0.029021233 | 0.345509549 |
| GOTERM_MF_DIRECT | GO:0019960~C-X3-C chemokine binding | 3 | 0.277264325 | 0.029021233 | 0.345509549 |
| GOTERM_MF_DIRECT | GO:0001077~transcriptional activator activity, RNA polymerase II core promoter proximal region sequence-specific binding | 22 | 2.033271719 | 0.029413434 | 0.345509806 |
| GOTERM_MF_DIRECT | GO:0004702~receptor signaling protein serine/threonine kinase activity | 8 | 0.739371534 | 0.030199385 | 0.350074452 |
| GOTERM_MF_DIRECT | GO:0000980~RNA polymerase II distal enhancer sequence-specific DNA binding | 9 | 0.831792976 | 0.031056222 | 0.353140609 |
| GOTERM_MF_DIRECT | GO:0005088~Ras guanyl-nucleotide exchange factor activity | 13 | 1.201478743 | 0.03126557 | 0.353140609 |
| GOTERM_MF_DIRECT | GO:0005244~voltage-gated ion channel activity | 6 | 0.554528651 | 0.03354313 | 0.369211686 |
| GOTERM_MF_DIRECT | GO:0042813~Wnt-activated receptor activity | 5 | 0.462107209 | 0.033945683 | 0.369211686 |
| GOTERM_MF_DIRECT | GO:0001530~lipopolysaccharide binding | 5 | 0.462107209 | 0.033945683 | 0.369211686 |
| GOTERM_MF_DIRECT | GO:0001784~phosphotyrosine binding | 4 | 0.369685767 | 0.034531136 | 0.370999159 |
| GOTERM_MF_DIRECT | GO:0044212~transcription regulatory region DNA binding | 20 | 1.848428835 | 0.036163777 | 0.383858887 |
| GOTERM_MF_DIRECT | GO:0005085~guanyl-nucleotide exchange factor activity | 13 | 1.201478743 | 0.037117295 | 0.389289726 |
| GOTERM_MF_DIRECT | GO:0043425~bHLH transcription factor binding | 5 | 0.462107209 | 0.039293443 | 0.407264983 |
| GOTERM_MF_DIRECT | GO:1990782~protein tyrosine kinase binding | 3 | 0.277264325 | 0.041902287 | 0.412399741 |
| GOTERM_MF_DIRECT | GO:0004875~complement receptor activity | 3 | 0.277264325 | 0.041902287 | 0.412399741 |
| GOTERM_MF_DIRECT | GO:0004185~serine-type carboxypeptidase activity | 4 | 0.369685767 | 0.042129372 | 0.412399741 |
| GOTERM_MF_DIRECT | GO:0004012~phospholipid-translocating ATPase activity | 4 | 0.369685767 | 0.042129372 | 0.412399741 |
| GOTERM_MF_DIRECT | GO:0045028~G-protein coupled purinergic nucleotide receptor activity | 4 | 0.369685767 | 0.042129372 | 0.412399741 |
| GOTERM_MF_DIRECT | GO:0005125~cytokine activity | 17 | 1.57116451 | 0.044547862 | 0.427126088 |
| GOTERM_MF_DIRECT | GO:0005031~tumor necrosis factor-activated receptor activity | 5 | 0.462107209 | 0.045093153 | 0.427126088 |
| GOTERM_MF_DIRECT | GO:0043621~protein self-association | 7 | 0.646950092 | 0.045573045 | 0.427126088 |
| GOTERM_MF_DIRECT | GO:0004715~non-membrane spanning protein tyrosine kinase activity | 7 | 0.646950092 | 0.045573045 | 0.427126088 |
| GOTERM_MF_DIRECT | GO:0001085~RNA polymerase II transcription factor binding | 7 | 0.646950092 | 0.049825998 | 0.454510092 |
| GOTERM_MF_DIRECT | GO:0001227~transcriptional repressor activity, RNA polymerase II transcription regulatory region sequence-specific binding | 8 | 0.739371534 | 0.049989352 | 0.454510092 |
| GOTERM_MF_DIRECT | GO:0032395~MHC class II receptor activity | 4 | 0.369685767 | 0.050488918 | 0.454510092 |
| GOTERM_MF_DIRECT | GO:0005525~GTP binding | 31 | 2.865064695 | 0.050558444 | 0.454510092 |
| GOTERM_MF_DIRECT | GO:0001205~transcriptional activator activity, RNA polymerase II distal enhancer sequence-specific binding | 5 | 0.462107209 | 0.051343916 | 0.456908991 |
| GOTERM_MF_DIRECT | GO:0004896~cytokine receptor activity | 6 | 0.554528651 | 0.052184005 | 0.459741086 |
| GOTERM_MF_DIRECT | GO:0005024~transforming growth factor beta-activated receptor activity | 3 | 0.277264325 | 0.056478373 | 0.473880445 |
| GOTERM_MF_DIRECT | GO:0005021~vascular endothelial growth factor-activated receptor activity | 3 | 0.277264325 | 0.056478373 | 0.473880445 |
| GOTERM_MF_DIRECT | GO:0008329~signaling pattern recognition receptor activity | 3 | 0.277264325 | 0.056478373 | 0.473880445 |
| GOTERM_MF_DIRECT | GO:0035005~1-phosphatidylinositol-4-phosphate 3-kinase activity | 3 | 0.277264325 | 0.056478373 | 0.473880445 |
| GOTERM_MF_DIRECT | GO:0019208~phosphatase regulator activity | 3 | 0.277264325 | 0.056478373 | 0.473880445 |
| GOTERM_MF_DIRECT | GO:0008134~transcription factor binding | 24 | 2.218114603 | 0.058828152 | 0.481599488 |
| GOTERM_MF_DIRECT | GO:0008009~chemokine activity | 7 | 0.646950092 | 0.059039588 | 0.481599488 |
| GOTERM_MF_DIRECT | GO:0008191~metalloendopeptidase inhibitor activity | 4 | 0.369685767 | 0.059584954 | 0.481599488 |
| GOTERM_MF_DIRECT | GO:0048018~receptor agonist activity | 4 | 0.369685767 | 0.059584954 | 0.481599488 |
| GOTERM_MF_DIRECT | GO:0019900~kinase binding | 9 | 0.831792976 | 0.063591003 | 0.509306124 |
| GOTERM_MF_DIRECT | GO:0004181~metallocarboxypeptidase activity | 5 | 0.462107209 | 0.065183544 | 0.517357677 |
| GOTERM_MF_DIRECT | GO:0043565~sequence-specific DNA binding | 39 | 3.604436229 | 0.067549873 | 0.531352124 |
| GOTERM_MF_DIRECT | GO:0005089~Rho guanyl-nucleotide exchange factor activity | 9 | 0.831792976 | 0.071957709 | 0.552596693 |
| GOTERM_MF_DIRECT | GO:0071723~lipopeptide binding | 3 | 0.277264325 | 0.072514314 | 0.552596693 |
| GOTERM_MF_DIRECT | GO:0031730~CCR5 chemokine receptor binding | 3 | 0.277264325 | 0.072514314 | 0.552596693 |
| GOTERM_MF_DIRECT | GO:0035035~histone acetyltransferase binding | 5 | 0.462107209 | 0.07275961 | 0.552596693 |
| GOTERM_MF_DIRECT | GO:0048365~Rac GTPase binding | 6 | 0.554528651 | 0.075802192 | 0.570784024 |
| GOTERM_MF_DIRECT | GO:0042803~protein homodimerization activity | 52 | 4.805914972 | 0.078825739 | 0.58852098 |
| GOTERM_MF_DIRECT | GO:0071837~HMG box domain binding | 4 | 0.369685767 | 0.07986515 | 0.591270561 |
| GOTERM_MF_DIRECT | GO:0042169~SH2 domain binding | 5 | 0.462107209 | 0.080761461 | 0.592923723 |
| GOTERM_MF_DIRECT | GO:0048495~Roundabout binding | 3 | 0.277264325 | 0.089796216 | 0.648446447 |
| GOTERM_MF_DIRECT | GO:0005522~profilin binding | 3 | 0.277264325 | 0.089796216 | 0.648446447 |
| GOTERM_MF_DIRECT | GO:0001228~transcriptional activator activity, RNA polymerase II transcription regulatory region sequence-specific binding | 10 | 0.924214418 | 0.09256301 | 0.662991967 |
| GOTERM_CC_DIRECT | GO:0031012~extracellular matrix | 78 | 7.208872458 | 2.29E-30 | 1.03E-27 |
| GOTERM_CC_DIRECT | GO:0005886~plasma membrane | 385 | 35.58225508 | 4.65E-27 | 1.04E-24 |
| GOTERM_CC_DIRECT | GO:0005578~proteinaceous extracellular matrix | 68 | 6.284658041 | 1.77E-25 | 2.65E-23 |
| GOTERM_CC_DIRECT | GO:0009986~cell surface | 92 | 8.502772643 | 6.26E-21 | 7.03E-19 |
| GOTERM_CC_DIRECT | GO:0009897~external side of plasma membrane | 53 | 4.898336414 | 1.46E-19 | 1.31E-17 |
| GOTERM_CC_DIRECT | GO:0005887~integral component of plasma membrane | 160 | 14.78743068 | 2.44E-17 | 1.81E-15 |
| GOTERM_CC_DIRECT | GO:0005576~extracellular region | 175 | 16.17375231 | 2.83E-17 | 1.81E-15 |
| GOTERM_CC_DIRECT | GO:0005788~endoplasmic reticulum lumen | 42 | 3.881700555 | 1.62E-13 | 9.10E-12 |
| GOTERM_CC_DIRECT | GO:0005615~extracellular space | 144 | 13.30868762 | 2.11E-13 | 1.06E-11 |
| GOTERM_CC_DIRECT | GO:0005925~focal adhesion | 62 | 5.73012939 | 7.68E-13 | 3.45E-11 |
| GOTERM_CC_DIRECT | GO:0005581~collagen trimer | 26 | 2.402957486 | 3.69E-11 | 1.51E-09 |
| GOTERM_CC_DIRECT | GO:0005604~basement membrane | 22 | 2.033271719 | 2.10E-09 | 7.86E-08 |
| GOTERM_CC_DIRECT | GO:0008305~integrin complex | 13 | 1.201478743 | 9.15E-09 | 3.16E-07 |
| GOTERM_CC_DIRECT | GO:0045121~membrane raft | 34 | 3.14232902 | 7.68E-08 | 2.46E-06 |
| GOTERM_CC_DIRECT | GO:0070062~extracellular exosome | 220 | 20.33271719 | 5.26E-07 | 1.57E-05 |
| GOTERM_CC_DIRECT | GO:0030027~lamellipodium | 27 | 2.495378928 | 1.37E-06 | 3.84E-05 |
| GOTERM_CC_DIRECT | GO:0005856~cytoskeleton | 43 | 3.974121996 | 1.95E-05 | 5.07E-04 |
| GOTERM_CC_DIRECT | GO:0015629~actin cytoskeleton | 30 | 2.772643253 | 2.03E-05 | 5.07E-04 |
| GOTERM_CC_DIRECT | GO:0043020~NADPH oxidase complex | 7 | 0.646950092 | 2.39E-05 | 5.64E-04 |
| GOTERM_CC_DIRECT | GO:0045335~phagocytic vesicle | 11 | 1.01663586 | 5.05E-05 | 0.001132911 |
| GOTERM_CC_DIRECT | GO:0016021~integral component of membrane | 353 | 32.62476895 | 5.32E-05 | 0.001137037 |
| GOTERM_CC_DIRECT | GO:0001527~microfibril | 6 | 0.554528651 | 1.21E-04 | 0.002466911 |
| GOTERM_CC_DIRECT | GO:0016020~membrane | 165 | 15.24953789 | 1.93E-04 | 0.003765777 |
| GOTERM_CC_DIRECT | GO:0005884~actin filament | 13 | 1.201478743 | 2.82E-04 | 0.005211807 |
| GOTERM_CC_DIRECT | GO:0002102~podosome | 8 | 0.739371534 | 2.90E-04 | 0.005211807 |
| GOTERM_CC_DIRECT | GO:0031410~cytoplasmic vesicle | 28 | 2.58780037 | 4.53E-04 | 0.007642684 |
| GOTERM_CC_DIRECT | GO:1903561~extracellular vesicle | 11 | 1.01663586 | 4.60E-04 | 0.007642684 |
| GOTERM_CC_DIRECT | GO:0005911~cell-cell junction | 22 | 2.033271719 | 8.58E-04 | 0.013763453 |
| GOTERM_CC_DIRECT | GO:0031093~platelet alpha granule lumen | 11 | 1.01663586 | 0.001009595 | 0.015631313 |
| GOTERM_CC_DIRECT | GO:0001726~ruffle | 14 | 1.293900185 | 0.001795781 | 0.026876854 |
| GOTERM_CC_DIRECT | GO:0032587~ruffle membrane | 13 | 1.201478743 | 0.002372955 | 0.034369576 |
| GOTERM_CC_DIRECT | GO:0043235~receptor complex | 17 | 1.57116451 | 0.002457028 | 0.034475172 |
| GOTERM_CC_DIRECT | GO:0001772~immunological synapse | 8 | 0.739371534 | 0.002732647 | 0.037180558 |
| GOTERM_CC_DIRECT | GO:0031234~extrinsic component of cytoplasmic side of plasma membrane | 11 | 1.01663586 | 0.005132585 | 0.067780313 |
| GOTERM_CC_DIRECT | GO:0030670~phagocytic vesicle membrane | 10 | 0.924214418 | 0.0060832 | 0.078038769 |
| GOTERM_CC_DIRECT | GO:0005942~phosphatidylinositol 3-kinase complex | 5 | 0.462107209 | 0.006748253 | 0.083266443 |
| GOTERM_CC_DIRECT | GO:0005794~Golgi apparatus | 68 | 6.284658041 | 0.0068616 | 0.083266443 |
| GOTERM_CC_DIRECT | GO:0005796~Golgi lumen | 13 | 1.201478743 | 0.00862817 | 0.098796184 |
| GOTERM_CC_DIRECT | GO:0043202~lysosomal lumen | 12 | 1.109057301 | 0.009051737 | 0.098796184 |
| GOTERM_CC_DIRECT | GO:0005588~collagen type V trimer | 3 | 0.277264325 | 0.00946155 | 0.098796184 |
| GOTERM_CC_DIRECT | GO:0005589~collagen type VI trimer | 3 | 0.277264325 | 0.00946155 | 0.098796184 |
| GOTERM_CC_DIRECT | GO:0005944~phosphatidylinositol 3-kinase complex, class IB | 3 | 0.277264325 | 0.00946155 | 0.098796184 |
| GOTERM_CC_DIRECT | GO:0098636~protein complex involved in cell adhesion | 3 | 0.277264325 | 0.00946155 | 0.098796184 |
| GOTERM_CC_DIRECT | GO:0001725~stress fiber | 9 | 0.831792976 | 0.01125378 | 0.113902469 |
| GOTERM_CC_DIRECT | GO:0005901~caveola | 10 | 0.924214418 | 0.011415615 | 0.113902469 |
| GOTERM_CC_DIRECT | GO:0005577~fibrinogen complex | 4 | 0.369685767 | 0.012134565 | 0.118443906 |
| GOTERM_CC_DIRECT | GO:0030666~endocytic vesicle membrane | 10 | 0.924214418 | 0.012566358 | 0.120048829 |
| GOTERM_CC_DIRECT | GO:0005667~transcription factor complex | 20 | 1.848428835 | 0.015087916 | 0.14113488 |
| GOTERM_CC_DIRECT | GO:0042101~T cell receptor complex | 5 | 0.462107209 | 0.017189025 | 0.157507593 |
| GOTERM_CC_DIRECT | GO:0071953~elastic fiber | 3 | 0.277264325 | 0.01820525 | 0.160277596 |
| GOTERM_CC_DIRECT | GO:0032010~phagolysosome | 3 | 0.277264325 | 0.01820525 | 0.160277596 |
| GOTERM_CC_DIRECT | GO:0030175~filopodium | 10 | 0.924214418 | 0.019649029 | 0.169265313 |
| GOTERM_CC_DIRECT | GO:0031226~intrinsic component of plasma membrane | 6 | 0.554528651 | 0.019980093 | 0.169265313 |
| GOTERM_CC_DIRECT | GO:0031258~lamellipodium membrane | 5 | 0.462107209 | 0.020807551 | 0.173010937 |
| GOTERM_CC_DIRECT | GO:0001931~uropod | 4 | 0.369685767 | 0.021875279 | 0.178581823 |
| GOTERM_CC_DIRECT | GO:0005769~early endosome | 22 | 2.033271719 | 0.022747794 | 0.182388564 |
| GOTERM_CC_DIRECT | GO:0042383~sarcolemma | 11 | 1.01663586 | 0.023197796 | 0.182733517 |
| GOTERM_CC_DIRECT | GO:0005938~cell cortex | 14 | 1.293900185 | 0.023967003 | 0.185537662 |
| GOTERM_CC_DIRECT | GO:0016023~cytoplasmic, membrane-bounded vesicle | 15 | 1.386321627 | 0.026897074 | 0.202843528 |
| GOTERM_CC_DIRECT | GO:0045177~apical part of cell | 10 | 0.924214418 | 0.027106039 | 0.202843528 |
| GOTERM_CC_DIRECT | GO:0043197~dendritic spine | 12 | 1.109057301 | 0.027847291 | 0.204974321 |
| GOTERM_CC_DIRECT | GO:0031941~filamentous actin | 6 | 0.554528651 | 0.030015041 | 0.217366989 |
| GOTERM_CC_DIRECT | GO:0030426~growth cone | 13 | 1.201478743 | 0.033871354 | 0.240512673 |
| GOTERM_CC_DIRECT | GO:0042613~MHC class II protein complex | 5 | 0.462107209 | 0.034296922 | 0.240512673 |
| GOTERM_CC_DIRECT | GO:0005614~interstitial matrix | 4 | 0.369685767 | 0.034818093 | 0.240512673 |
| GOTERM_CC_DIRECT | GO:0046696~lipopolysaccharide receptor complex | 3 | 0.277264325 | 0.042150399 | 0.284635869 |
| GOTERM_CC_DIRECT | GO:0031091~platelet alpha granule | 4 | 0.369685767 | 0.042473504 | 0.284635869 |
| GOTERM_CC_DIRECT | GO:0016324~apical plasma membrane | 25 | 2.310536044 | 0.04778076 | 0.315493551 |
| GOTERM_CC_DIRECT | GO:0005605~basal lamina | 4 | 0.369685767 | 0.050894246 | 0.331181395 |
| GOTERM_CC_DIRECT | GO:0032059~bleb | 3 | 0.277264325 | 0.056805661 | 0.3592358 |
| GOTERM_CC_DIRECT | GO:0032045~guanyl-nucleotide exchange factor complex | 3 | 0.277264325 | 0.056805661 | 0.3592358 |
| GOTERM_CC_DIRECT | GO:0005764~lysosome | 20 | 1.848428835 | 0.060824082 | 0.377397225 |
| GOTERM_CC_DIRECT | GO:0048471~perinuclear region of cytoplasm | 46 | 4.251386322 | 0.061996434 | 0.377397225 |
| GOTERM_CC_DIRECT | GO:0043025~neuronal cell body | 26 | 2.402957486 | 0.062199097 | 0.377397225 |
| GOTERM_CC_DIRECT | GO:0005737~cytoplasm | 322 | 29.75970425 | 0.067349349 | 0.403198102 |
| GOTERM_CC_DIRECT | GO:0097060~synaptic membrane | 4 | 0.369685767 | 0.08047327 | 0.475427606 |
| GOTERM_CC_DIRECT | GO:0009898~cytoplasmic side of plasma membrane | 6 | 0.554528651 | 0.090402699 | 0.5271534 |
| GOTERM_CC_DIRECT | GO:0031594~neuromuscular junction | 7 | 0.646950092 | 0.093462798 | 0.531199953 |
| GOTERM_CC_DIRECT | GO:0005905~clathrin-coated pit | 7 | 0.646950092 | 0.093462798 | 0.531199953 |
