# Supplementary material for: A Potential Prognostic Marker PRDM1 in Pancreatic Adenocarcinoma
Source: J Oncol. 2022 May 13;2022:1934381. doi: 10.1155/2022/1934381 (PMC9123419; doi:10.1155/2022/1934381)
Supplement: Supplementary 4 — Table S4: the coexpressed genes with negative correlation between GO enrichment pathway and PRDM1 via DAVID database. [file 1934381.f4.docx]

**Table S4.** The co-expressed genes with negative correlation between GO enrichment pathway and PRDM1 via David database.

| Category | Term | Count | Ratio | P-value | Genes | FDR |
| --- | --- | --- | --- | --- | --- | --- |
| BP | GO:0055114~oxidation-reduction process | 11 | 9.322033898 | 0.00203846 | DHRS13, SURF1, ALKBH4, SPR, PYCRL, PTGES2, PCBD1, ALKBH7, ECSIT, P4HTM, GFER | 0.298974183 |
| BP | GO:0032981~mitochondrial respiratory chain complex I assembly | 7 | 5.93220339 | 1.56E-06 | NDUFA7, NDUFB7, NDUFB10, NDUFS6, NDUFB11, NDUFC1, ECSIT | 6.85E-04 |
| BP | GO:0006120~mitochondrial electron transport, NADH to ubiquinone | 6 | 5.084745763 | 8.59E-06 | NDUFA7, NDUFB7, NDUFB10, NDUFS6, NDUFB11, NDUFC1 | 0.001890251 |
| CC | GO:0005739~mitochondrion | 30 | 25.42372881 | 6.18E-10 | NDUFB7, NDUFB11, TUSC2, RAB3D, MRPS34, TIMM13, LIAS, ECSIT, CISD3, TMEM186, CHCHD2, COMTD1, NTHL1, ROMO1, FIS1, TRAP1, ACOT8, NDUFA7, MRPS26, ENDOG, TSTD1, PTGES2, MDH2, NDUFC1, MCAT, GFER, COQ4, C6ORF136, SDHAF1, JTB | 7.60E-08 |
| CC | GO:0005743~mitochondrial inner membrane | 17 | 14.40677966 | 8.01E-09 | TRAP1, SURF1, MRPS26, NDUFA7, NDUFB7, NDUFB10, NDUFB11, MDH2, MRPS34, MRPL38, TIMM13, NDUFC1, ECSIT, COQ4, C19ORF70, NDUFS6, ROMO1 | 4.93E-07 |
| CC | GO:0005747~mitochondrial respiratory chain complex I | 6 | 5.084745763 | 1.08E-05 | NDUFA7, NDUFB7, NDUFB10, NDUFS6, NDUFB11, NDUFC1 | 4.43E-04 |
| CC | GO:0005758~mitochondrial intermembrane space | 5 | 4.237288136 | 0.001009958 | TRAP1, CHCHD5, CHCHD2, NDUFB7, GFER | 0.031056198 |
| MF | GO:0008137~NADH dehydrogenase (ubiquinone) activity | 5 | 4.237288136 | 1.71E-04 | NDUFA7, NDUFB7, NDUFB10, NDUFS6, NDUFC1 | 0.031433517 |
