# Supplementary material for: A Potential Prognostic Marker PRDM1 in Pancreatic Adenocarcinoma
Source: J Oncol. 2022 May 13;2022:1934381. doi: 10.1155/2022/1934381 (PMC9123419; doi:10.1155/2022/1934381)
Supplement: Supplementary 5 — Table S5: the coexpressed genes with positive correlation between KEGG pathway and PRDM1 via DAVID database. [file 1934381.f5.docx]

**Table S5.** The co-expressed genes with positive correlation between KEGG pathway and PRDM1 via David database.

| Category | Term | Count | Ratio | P-value | FDR |
| --- | --- | --- | --- | --- | --- |
| KEGG_PATHWAY | hsa05200:Pathways in cancer | 58 | 5.360443623 | 4.82E-09 | 1.08E-07 |
| KEGG_PATHWAY | hsa04151:PI3K-Akt signaling pathway | 54 | 4.990757856 | 2.26E-09 | 5.82E-08 |
| KEGG_PATHWAY | hsa04510:Focal adhesion | 45 | 4.15896488 | 9.09E-13 | 4.09E-11 |
| KEGG_PATHWAY | hsa04060:Cytokine-cytokine receptor interaction | 42 | 3.881700555 | 1.12E-08 | 2.24E-07 |
| KEGG_PATHWAY | hsa04380:Osteoclast differentiation | 37 | 3.419593346 | 3.80E-14 | 4.67E-12 |
| KEGG_PATHWAY | hsa04514:Cell adhesion molecules (CAMs) | 36 | 3.327171904 | 2.88E-12 | 1.04E-10 |
| KEGG_PATHWAY | hsa05166:HTLV-I infection | 35 | 3.234750462 | 4.20E-05 | 3.43E-04 |
| KEGG_PATHWAY | hsa05205:Proteoglycans in cancer | 34 | 3.14232902 | 5.50E-07 | 7.07E-06 |
| KEGG_PATHWAY | hsa04810:Regulation of actin cytoskeleton | 33 | 3.049907579 | 4.85E-06 | 4.85E-05 |
| KEGG_PATHWAY | hsa04145:Phagosome | 31 | 2.865064695 | 2.26E-08 | 4.07E-07 |
| KEGG_PATHWAY | hsa05152:Tuberculosis | 30 | 2.772643253 | 3.15E-06 | 3.55E-05 |
| KEGG_PATHWAY | hsa04015:Rap1 signaling pathway | 30 | 2.772643253 | 8.81E-05 | 6.34E-04 |
| KEGG_PATHWAY | hsa04512:ECM-receptor interaction | 29 | 2.680221811 | 3.89E-13 | 2.34E-11 |
| KEGG_PATHWAY | hsa05146:Amoebiasis | 28 | 2.58780037 | 4.36E-10 | 1.31E-08 |
| KEGG_PATHWAY | hsa04611:Platelet activation | 28 | 2.58780037 | 5.08E-08 | 8.31E-07 |
| KEGG_PATHWAY | hsa04062:Chemokine signaling pathway | 28 | 2.58780037 | 6.48E-05 | 4.86E-04 |
| KEGG_PATHWAY | hsa04014:Ras signaling pathway | 25 | 2.310536044 | 0.011984251 | 0.043143305 |
| KEGG_PATHWAY | hsa05150:Staphylococcus aureus infection | 24 | 2.218114603 | 5.18E-14 | 4.67E-12 |
| KEGG_PATHWAY | hsa04010:MAPK signaling pathway | 24 | 2.218114603 | 0.065379535 | 0.186798671 |
| KEGG_PATHWAY | hsa04670:Leukocyte transendothelial migration | 23 | 2.125693161 | 3.83E-06 | 4.06E-05 |
| KEGG_PATHWAY | hsa04144:Endocytosis | 23 | 2.125693161 | 0.06779242 | 0.190666181 |
| KEGG_PATHWAY | hsa05162:Measles | 22 | 2.033271719 | 1.26E-04 | 8.75E-04 |
| KEGG_PATHWAY | hsa05202:Transcriptional misregulation in cancer | 22 | 2.033271719 | 0.00270361 | 0.012478199 |
| KEGG_PATHWAY | hsa04640:Hematopoietic cell lineage | 21 | 1.940850277 | 5.13E-07 | 7.07E-06 |
| KEGG_PATHWAY | hsa04630:Jak-STAT signaling pathway | 21 | 1.940850277 | 0.0010997 | 0.005998363 |
| KEGG_PATHWAY | hsa04390:Hippo signaling pathway | 21 | 1.940850277 | 0.001819494 | 0.009097469 |
| KEGG_PATHWAY | hsa05164:Influenza A | 21 | 1.940850277 | 0.009227022 | 0.035906049 |
| KEGG_PATHWAY | hsa05140:Leishmaniasis | 20 | 1.848428835 | 7.51E-08 | 1.13E-06 |
| KEGG_PATHWAY | hsa05323:Rheumatoid arthritis | 20 | 1.848428835 | 2.73E-06 | 3.27E-05 |
| KEGG_PATHWAY | hsa04974:Protein digestion and absorption | 19 | 1.756007394 | 1.12E-05 | 1.06E-04 |
| KEGG_PATHWAY | hsa04620:Toll-like receptor signaling pathway | 19 | 1.756007394 | 1.49E-04 | 9.92E-04 |
| KEGG_PATHWAY | hsa05145:Toxoplasmosis | 19 | 1.756007394 | 2.41E-04 | 0.001549136 |
| KEGG_PATHWAY | hsa04660:T cell receptor signaling pathway | 16 | 1.478743068 | 0.001947052 | 0.009472145 |
| KEGG_PATHWAY | hsa05142:Chagas disease (American trypanosomiasis) | 16 | 1.478743068 | 0.002884816 | 0.01298167 |
| KEGG_PATHWAY | hsa04650:Natural killer cell mediated cytotoxicity | 16 | 1.478743068 | 0.012710153 | 0.044859363 |
| KEGG_PATHWAY | hsa05416:Viral myocarditis | 15 | 1.386321627 | 1.19E-05 | 1.07E-04 |
| KEGG_PATHWAY | hsa05222:Small cell lung cancer | 15 | 1.386321627 | 0.001087964 | 0.005998363 |
| KEGG_PATHWAY | hsa04668:TNF signaling pathway | 15 | 1.386321627 | 0.009375468 | 0.035906049 |
| KEGG_PATHWAY | hsa05169:Epstein-Barr virus infection | 15 | 1.386321627 | 0.027150185 | 0.087268451 |
| KEGG_PATHWAY | hsa05100:Bacterial invasion of epithelial cells | 14 | 1.293900185 | 0.001451956 | 0.007686824 |
| KEGG_PATHWAY | hsa04064:NF-kappa B signaling pathway | 14 | 1.293900185 | 0.003944271 | 0.017316311 |
| KEGG_PATHWAY | hsa04360:Axon guidance | 14 | 1.293900185 | 0.070008146 | 0.193868711 |
| KEGG_PATHWAY | hsa04672:Intestinal immune network for IgA production | 13 | 1.201478743 | 3.30E-05 | 2.83E-04 |
| KEGG_PATHWAY | hsa05144:Malaria | 13 | 1.201478743 | 5.17E-05 | 4.05E-04 |
| KEGG_PATHWAY | hsa05321:Inflammatory bowel disease (IBD) | 13 | 1.201478743 | 7.52E-04 | 0.004512911 |
| KEGG_PATHWAY | hsa05414:Dilated cardiomyopathy | 13 | 1.201478743 | 0.007922349 | 0.031689397 |
| KEGG_PATHWAY | hsa04270:Vascular smooth muscle contraction | 13 | 1.201478743 | 0.078674346 | 0.208826047 |
| KEGG_PATHWAY | hsa04610:Complement and coagulation cascades | 12 | 1.109057301 | 0.004703943 | 0.020159757 |
| KEGG_PATHWAY | hsa05410:Hypertrophic cardiomyopathy (HCM) | 12 | 1.109057301 | 0.011847146 | 0.043143305 |
| KEGG_PATHWAY | hsa04666:Fc gamma R-mediated phagocytosis | 12 | 1.109057301 | 0.019947315 | 0.0677456 |
| KEGG_PATHWAY | hsa05320:Autoimmune thyroid disease | 11 | 1.01663586 | 0.001675041 | 0.008614497 |
| KEGG_PATHWAY | hsa05412:Arrhythmogenic right ventricular cardiomyopathy (ARVC) | 11 | 1.01663586 | 0.010952352 | 0.041071322 |
| KEGG_PATHWAY | hsa05218:Melanoma | 11 | 1.01663586 | 0.016177553 | 0.055999222 |
| KEGG_PATHWAY | hsa05133:Pertussis | 11 | 1.01663586 | 0.023057785 | 0.075461842 |
| KEGG_PATHWAY | hsa05215:Prostate cancer | 11 | 1.01663586 | 0.059646017 | 0.17600464 |
| KEGG_PATHWAY | hsa04066:HIF-1 signaling pathway | 11 | 1.01663586 | 0.094729161 | 0.242583378 |
| KEGG_PATHWAY | hsa05330:Allograft rejection | 10 | 0.924214418 | 4.76E-04 | 0.002957435 |
| KEGG_PATHWAY | hsa05220:Chronic myeloid leukemia | 10 | 0.924214418 | 0.043450058 | 0.132559498 |
| KEGG_PATHWAY | hsa04350:TGF-beta signaling pathway | 10 | 0.924214418 | 0.095685666 | 0.242583378 |
| KEGG_PATHWAY | hsa05332:Graft-versus-host disease | 9 | 0.831792976 | 0.001005666 | 0.005839354 |
| KEGG_PATHWAY | hsa04940:Type I diabetes mellitus | 9 | 0.831792976 | 0.00509848 | 0.021342476 |
| KEGG_PATHWAY | hsa05217:Basal cell carcinoma | 9 | 0.831792976 | 0.022723482 | 0.075461842 |
| KEGG_PATHWAY | hsa05221:Acute myeloid leukemia | 9 | 0.831792976 | 0.02771389 | 0.087517548 |
| KEGG_PATHWAY | hsa05212:Pancreatic cancer | 9 | 0.831792976 | 0.059474248 | 0.17600464 |
| KEGG_PATHWAY | hsa05211:Renal cell carcinoma | 9 | 0.831792976 | 0.064012637 | 0.18584314 |
| KEGG_PATHWAY | hsa04664:Fc epsilon RI signaling pathway | 9 | 0.831792976 | 0.073719785 | 0.201053958 |
| KEGG_PATHWAY | hsa04662:B cell receptor signaling pathway | 9 | 0.831792976 | 0.07888984 | 0.208826047 |
| KEGG_PATHWAY | hsa05310:Asthma | 8 | 0.739371534 | 0.002629995 | 0.012457871 |
| KEGG_PATHWAY | hsa05340:Primary immunodeficiency | 8 | 0.739371534 | 0.005543098 | 0.022676311 |
| KEGG_PATHWAY | hsa00532:Glycosaminoglycan biosynthesis - chondroitin sulfate / dermatan sulfate | 5 | 0.462107209 | 0.037650837 | 0.116847425 |
| KEGG_PATHWAY | hsa04340:Hedgehog signaling pathway | 5 | 0.462107209 | 0.095658499 | 0.242583378 |
