# Supplementary material for: A Potential Prognostic Marker PRDM1 in Pancreatic Adenocarcinoma
Source: J Oncol. 2022 May 13;2022:1934381. doi: 10.1155/2022/1934381 (PMC9123419; doi:10.1155/2022/1934381)
Supplement: Supplementary 6 — Table S6: the coexpressed genes with negative correlation between KEGG pathway and PRDM1 via DAVID database. [file 1934381.f6.docx]

**Table S6.** The co-expressed genes with negative correlation between KEGG pathway and PRDM1 via David database.

| Category | Term | Count | Ratio | P-value | Genes | FDR |
| --- | --- | --- | --- | --- | --- | --- |
| KEGG_PATHWAY | hsa01100:Metabolic pathways | 22 | 18.6440678 | 5.94E-07 | ACOT8, NDUFA7, NDUFB7, NDUFB10, PYCRL, MMAB, NDUFB11, PTGES2, GPAA1, MDH2, ALG5, NDUFC1, ATP5G2, LIAS, MCAT, DPM3, SPR, NDUFS6, POLD2, PMVK, INPP5J, POLR2I | 3.15E-05 |
| KEGG_PATHWAY | hsa05016:Huntington's disease | 8 | 6.779661017 | 1.01E-04 | NDUFA7, NDUFB7, NDUFB10, NDUFS6, NDUFB11, NDUFC1, ATP5G2, POLR2I | 0.00184359 |
| KEGG_PATHWAY | hsa00190:Oxidative phosphorylation | 7 | 5.93220339 | 1.04E-04 | NDUFA7, NDUFB7, NDUFB10, NDUFS6, NDUFB11, NDUFC1, ATP5G2 | 0.00184359 |
| KEGG_PATHWAY | hsa05012:Parkinson's disease | 7 | 5.93220339 | 1.50E-04 | NDUFA7, NDUFB7, NDUFB10, NDUFS6, NDUFB11, NDUFC1, ATP5G2 | 0.001985445 |
| KEGG_PATHWAY | hsa05010:Alzheimer's disease | 7 | 5.93220339 | 3.74E-04 | NDUFA7, NDUFB7, NDUFB10, NDUFS6, NDUFB11, NDUFC1, ATP5G2 | 0.003964125 |
| KEGG_PATHWAY | hsa04932:Non-alcoholic fatty liver disease (NAFLD) | 6 | 5.084745763 | 0.001688704 | NDUFA7, NDUFB7, NDUFB10, NDUFS6, NDUFB11, NDUFC1 | 0.014916886 |
| KEGG_PATHWAY | hsa04146:Peroxisome | 3 | 2.542372881 | 0.083576911 | ACOT8, PXMP2, PMVK | 0.632796613 |
